# Targeting Supramolecular Active Complexes of Na_v_1.7/Na_v_1.8 to Relieve Chronic Neuropathic Pain

**DOI:** 10.1002/advs.202522185

**Published:** 2026-05-20

**Authors:** Liting Sun, Hang Xian, Yunxin Shi, Taotan Yang, Hongyan Shuai, Wenchao Hu, Siying Fei, Miao Xu, Taoyuan Yang, Ruilong Xia, Ting Wen, Fengting Zhu, Yan Fu, Yang Li, Wei Xia, Ran Qian, Yuanying Liu, Zhicheng Tian, Lamei Li, Qian Zhou, Lize Xiong, Rui Cong, Ceng Luo, Shengxi Wu, Xiafeng Shen, Xin Yu, Rou‐Gang Xie, Changgeng Peng

**Affiliations:** ^1^ The First Rehabilitation Hospital of Shanghai Brain and Spinal Cord Innovation Research Center Translational Research Institute of Brain and Brain‐Like Intelligence，Shanghai Key Laboratory of Anesthesiology and Brain Functional Modulation Clinical Research Center For Anesthesiology and Perioperative Medicine School of Medicine Tongji University Shanghai China; ^2^ Department of Neurobiology School of Basic Medicine Fourth Military Medical University Xi'an China; ^3^ Department of Orthopedics Xijing Hospital Fourth Military Medical University Xi'an China; ^4^ Xiang‐Xing College Hunan University of Traditional Chinese Medicine Changsha China; ^5^ Pre‐clinical College Dali University Dali Yunnan China; ^6^ Department of Anesthesiology and Perioperative medicine Shanghai Fourth People's Hospital Shanghai China; ^7^ The Shaanxi Province Key Laboratory of Brain Function Analysis and Modulation Xi'an China

**Keywords:** chronic pain, Creb, Dsp, pathological hallmark, Sptan1, targeted therapy, TrkB

## Abstract

Neuropathic pain (NP) affects 7%–10% of population, with current treatments often proving inadequate. Here we show that Na_v_1.7 and Na_v_1.8 form supramolecular active complexes (SMACs) with polygonal lattice structure in dorsal root ganglion (DRG) neurons of mouse models and patients with severe chronic NP. TrkB signaling facilitates the formation of Na_v_1.7/Na_v_1.8 SMACs. Targeting these SMACs with combined Na_v_1.7 and Na_v_1.8 blockers inhibits action potentials of both human and mouse pathological DRG neurons and synergistically alleviates chronic NP in spared nerve injury (SNI) and diabetic mouse models. The SMAC formation is promoted by five cytoskeletal proteins (SPTAN1, DSP, AHNAK, MPZ and PRX). Functional study demonstrates that these SMACs create a Na^+^ potential difference to amplify sodium currents, promoting DRG neuron hyperexcitability. Moreover, knockdown of these five cytoskeletal proteins prevents action potential generation in DRG neurons and eliminates NP in SNI mice. Our findings support that SMACs can be a potential pathological hallmark and novel promising therapeutic target for severe chronic NP.

## Graphical abstract

In mice and patients with severe chronic neuropathic pain (NP), Na_v_1.7, Na_v_1.8, TrkB, and five cytoskeletal proteins form supramolecular active complexes (SMACs) with polygonal lattice structures as noxious signal amplifiers in dorsal root ganglion (DRG) neurons. Inhibiting or knocking down SMAC components reduces DRG hyperexcitability and alleviates NP in mice, suggesting their therapeutic potential.

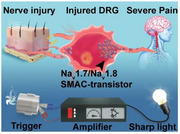

## 1 Introduction

NP affects 7%‐10% of the global population [1, 2], yet the effectiveness of available treatments remains limited due to undiscovered underlying mechanisms [3]. This type of pain is associated with the alteration of expression and posttranslational modifications of voltage‐gated sodium channels Na_v_1.7 (also known as SCN9A and PN1) [4, 5, 6] and Na_v_1.8 (also known as SCN10A, PN3 and SNS) [7, 8, 9], which are members of the voltage‐gated sodium channel family. Gain‐of‐function mutations in either Na_v_1.7 or Na_v_1.8 can lead to painful small‐fiber neuropathies [10, 11, 12, 13, 14, 15, 16]. Conversely, loss‐of‐function mutations in Na_v_1.7 result in congenital insensitivity to pain [17, 18, 19], while knockout of Na_v_1.8 raises thresholds of mechanical pain and inflammatory pain, but not NP [20, 21]. Despite the strong clinical relevance of Na_v_1.7 and Na_v_1.8 in NP, the second‐generation sodium channel blockers selectively targeting Na_v_1.7 have yet to demonstrate effectiveness in alleviating NP in patients [22, 23, 24]. While selective Na_v_1.8 blockers have shown promise in preclinical models and early clinical studies for NP, including positive Phase 2 signals with suzetrigine (VX‐548, approved for moderate‐to‐severe acute pain), they have yet to demonstrate definitive effectiveness in alleviating NP in late‐stage clinical trials in patients. This suggests that the development of NP may involve critical alterations in Na_v_1.7 and Na_v_1.8 functions.

Clustering of receptors in membranes can amplify the downstream signals induced by the same amount of ligand [25, 26, 27], and changes in the size and geometric structure of SMAC‐containing receptors can further boost signaling amplitude [26, 28, 29]. In the vertebrate nervous system, voltage‐gated sodium channels cluster at high density in specialized axonal domains to ensure rapid and reliable action potential initiation and propagation. At nodes of Ranvier — the unmyelinated gaps between myelin segments — sodium channels are physiologically clustered to enable saltatory conduction. The predominant isoforms at developing and mature nodes are Na_v_1.2 and Na_v_1.6, whose clustering depends on axonal cytoskeletal scaffolds such as ankyrin‐G and βIV‐Spectrin. In contrast, Na_v_1.7 and Na_v_1.8, two tetrodotoxin‐sensitive and ‐resistant isoforms highly expressed in DRG neurons, have been reported to show no enrichment at nodes of Ranvier under physiological conditions [30, 31]. Although upregulation and altered function of Na_v_1.7 and Na_v_1.8 have been extensively linked to neuronal hyperexcitability in NP—and recent studies have begun to explore the functional interactions between WT Na_v_1.8 and Na_v_1.7L848H (a gain‐of‐function Na_v_1.7 mutation that causes inherited erythromelalgia [32]) at subthreshold voltages in DRG neurons—supramolecular clustering of Na_v_1.7 and Na_v_1.8 into pathological assemblies has not been previously reported.

Here, we identify pathological Na_v_1.7/Na_v_1.8 channel SMACs that form in TrkB^+^ LTMR and small neurons of mouse and human DRG during the development of chronic NP [33]. Using high‐resolution imaging and functional analyses, we resolve the nanoscale organization, molecular composition, and functional significance of these injury‐induced SMACs. Our findings reveal that SMACs represent a previously unrecognized pathological reorganization of Na_v_1.7 and Na_v_1.8 that drives sustained hyperexcitability and contributes to the maintenance of refractory NP.

## 2 Results

### 2.1 Formation of SMACs Containing Na_v_1.7/Na_v_1.8/TrkB in DRG Neurons of Mice With NP

Given the co‐upregulation of Na_v_1.7 and Na_v_1.8 observed in *miR‐96* knockout mice exhibiting pain‐like behaviors [34], it's plausible that the proportion of neurons co‐expressing Na_v_1.7 and Na_v_1.8 may increase following nerve injury. We found that both Na_v_1.7 and Na_v_1.8 were upregulated in DRG of lumbar spinal segment 4–6 (L4‐6) in mice at 6 weeks post ‐ injury, compared to naïve mice (Figure S1A,B). Further detailed analysis through immunostaining of DRG sections unveiled a nuanced dynamic: when compared to sham‐operated mice, the proportion of neurons expressing Na_v_1.7 or both channels significantly increased in L4 DRG but showed a decline in L5 DRG by 2 weeks post‐injury. This trend of increased expression eventually diminished in both L4 and L5 DRG by 6 weeks post‐injury (Figure S1C–H). The observed reduction in neurons co‐expressing Na_v_1.7 and Na_v_1.8 in SNI mice was accompanied by an increased apoptosis rate compared to sham mice, with a notably higher incidence of cell death in L5 DRG than in L4 DRG at 2 weeks post‐injury (Figure S2A–D). Moreover, this alteration may also involve downregulation of sodium channel expression below detection thresholds.

**FIGURE 1:**
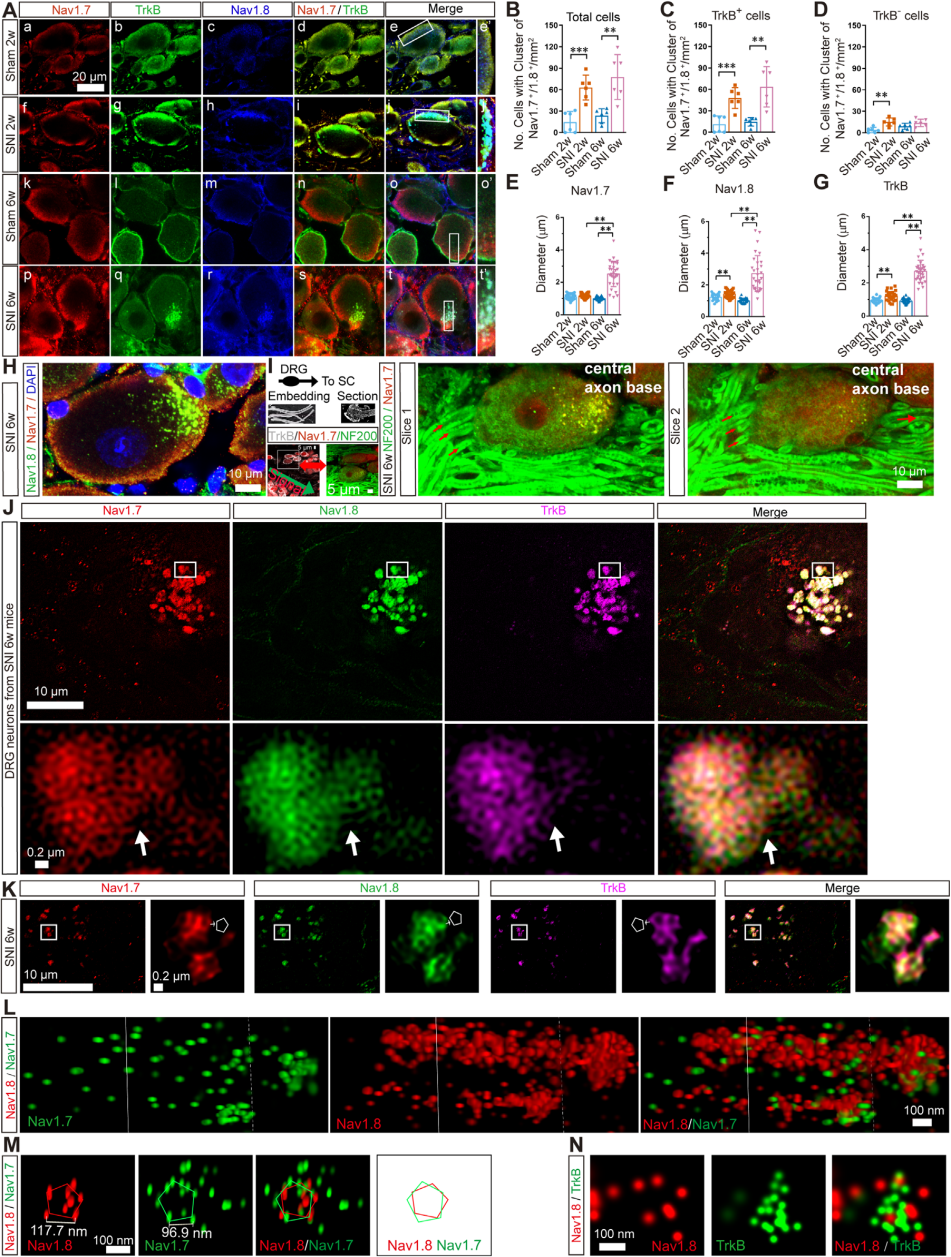
Nerve injury induces formation of SMACs of Na_v_1.7 and Na_v_1.8 in DRG neurons of mice. (A) Immunostaining against Na_v_1.7, Na_v_1.8, and TrkB in DRG sections from sham mice (a‐e', k‐o') and injured mice (f‐j', p‐t) at 2 weeks or 6 weeks post‐injury. The far‐right column displays magnified views of the boxed areas highlighted in the adjacent column. (B–D) Numbers of (B) total cells, (C) TrkB‐expressing cells, or (D) non‐TrkB‐expressing cells containing clusters of Na_v_1.7 and Na_v_1.8. *n* = 6 animals per condition. (E–G) Diameters of intraneuronal clusters containing Na_v_1.7 (E), Na_v_1.8 (F) or TrkB (G). *n* = 30 clusters in each case. (H) Immunostaining against Na_v_1.7 and Na_v_1.8 revealed portion of SMAC located on the plasma membrane. (I) Immunostaining against Na_v_1.7 (red), NF200 (green) and TrkB (grey) demonstrated the polar location of SMAC at the base of axon projecting toward the spinal cord. DRGs with longer axons projecting toward the spinal cord embedded in a consistent orientation. Right two images are high magnification view of the neuron boxed in left image (double head arrow connects), arrows point the direction of signal process direction. (J) Images of immunostained SMAC of Na_v_1.7 (red) and Na_v_1.8 (green) and TrkB (magenta) in DRG section from mice at 6 weeks post‐injury (60 nm resolution, Eryla). Lower panel is magnification view of the boxed area in upper panel. Arrows point to the linker region between two clusters in the SMAC. (K) Hessian structured illumination microscopy (HIS‐SIM) images showed that Na_v_1.7, Na_v_1.8, and TrkB formed polygonal lattice cluster. Adjacent right images are high magnification view of the boxed region in left images. (L–N) **S**TORM image showed 3D view (L), a gradient of Na_v_1.7 and Na_v_1.8 along long axis (from left to right), and one slice view (M, N) of Na_v_1.7, Na_v_1.8 and TrkB, some clusters appearing pentagons (M). * *p* < 0.05, ** *p* < 0.01, *** *p* < 0.001 by unpaired Student's *t* test (B–D) or by Kruskal‐Wallis test (E–G).

Under 60 × objective lens, observation revealed that neurons co‐expressing Na_v_1.7 and Na_v_1.8 in DRG neurons from SNI mice at 2 weeks post‐injury exhibited small clusters of Na_v_1.7, Na_v_1.8, and TrkB localized both on and along plasma membrane (Figure 1A). By 6 weeks post‐injury, these clusters aggregated and formed large SMACs, comprising dozens of tightly packed clusters in injured animals, a phenomenon not observed in sham controls (Figure 1A). Quantitative analysis showed that the number of cells containing Na_v_1.7/Na_v_1.8 clusters was dramatically higher in DRG of SNI mice than in sham mice at both 2 and 6 weeks post‐injury (Figure 1B). These clusters predominantly appeared in TrkB‐expressing neurons (Figure 1C,D). In DRG neurons of SNI mice, the average diameter of Na_v_1.7 clusters in SMACs was significantly larger at 6 weeks after nerve injury (Figure 1E), and the diameters of Na_v_1.8 clusters and TrkB clusters in SMACs were remarkably larger from 2 to 6 weeks after nerve injury than in sham mice (Figure 1F,G). Portion of Na_v_1.7/Na_v_1.8 SMACs localized on the plasma membrane and specifically at the base of the axon projecting toward the spinal cord (Figure 1H,I). This localization was further confirmed by co‐staining with Dil, a lipophilic membrane dye (Figure S3A–H). The presence of small Na_v_1.7/Na_v_1.8 clusters in a few DRG neurons in sham‐operated mice suggests that postoperative pain, especially during the wound healing phase, may also be associated with qualitative alterations in distribution or organization of Na_v_1.7 and Na_v_1.8.

### 2.2 Structure of SMAC

In our structural analysis of SMAC, super‐resolution imaging (60 nm) of cultured neurons from SNI mice at 6 weeks post‐injury unveiled an intriguing architectural organization. Na_v_1.7, Na_v_1.8, and TrkB were observed forming polygonal lattice columnar structure within SMAC, with side length ranging approximately from 160 to 250 nm, The clusters in the SMAC were linked together by proteins of Na_v_1.7, Na_v_1.8, and TrkB (Figure 1J,K). Further refinement utilizing Stochastic Optical Reconstruction Microscopy (STORM), with a resolution of 20 nm, unveiled a gradient density distribution of Na_v_1.7, Na_v_1.8, and TrkB along the long axis of the columnar lattice, the average densities of Na_v_1.7 and Na_v_1.8 were estimated proximately 340 molecules/µm^3^ (range: 90–1272 molecules/µm^3^) and 902 molecule/µm^3^ (range: 271–2900 molecules/µm^3^), respectively (Figure 1L–N; Figure S4A–L, Videos S1–S3, and Table S1). Notably, within the most densely packed core regions of these clusters, intermolecular distances frequently reached ∼30 nm between molecules of different types and ∼70 nm between molecules of the same type, with many instances approaching near‐contact configurations in injured DRG neurons of mice.

**FIGURE 2:**
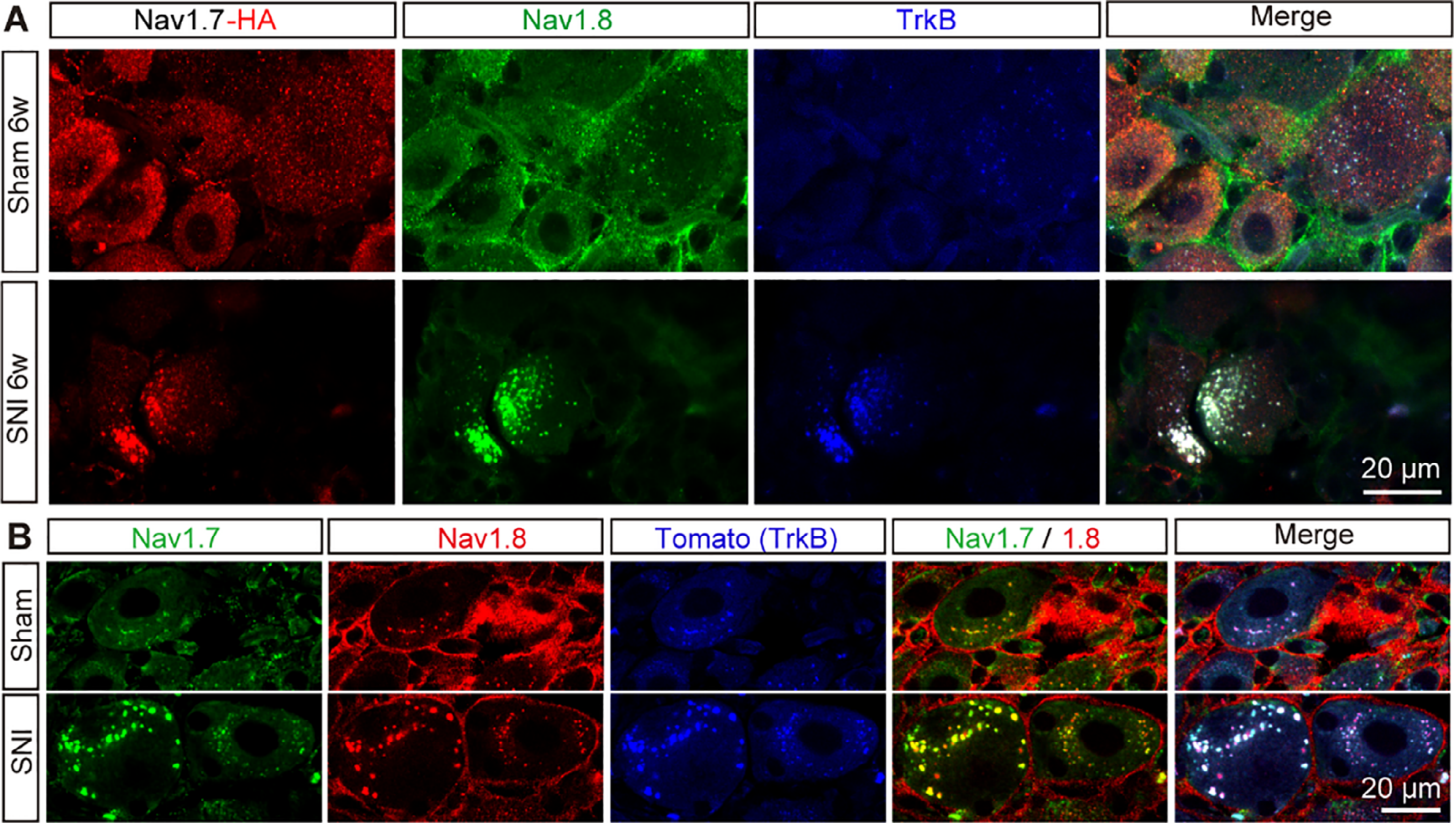
Presence of Na_v_1.7 and TrkB in SMACs of DRG neurons in SNI mice. (A) Immunostaining against HA, Na_v_1.8, and TrkB in sections from sham or SNI *Scn9a^HA/+^* SNI mice at 6 weeks post‐injury. (B) Immunostaining against Na_v_1.7, Na_v_1.8 and Tomato in sections from sham or SNI *TrkB^2A‐Tomato/+^* mice at 6 weeks post‐injury.

To confirm the presence of Na_v_1.7 within SMACs, we utilized *Scn9a^HA/+^* tag mice, which exhibited mechanical pain thresholds comparable to their *wild‐type* (*WT*) littermates (Figure S5A–C). In these mice, HA tagging effectively marked nearly all Na_v_1.7‐expressing neurons in sham and SNI *Scn9a^HA/+^* mice at both 2 weeks (97.6% ± 2.69%, 99.4% ± 0.41%) and 6 weeks after nerve injury (97.3% ± 2.77%, 99.1% ± 0.93%) (Figure S5D–F), indicating all Na_v_1.7‐expressing neurons expressed the isoform of NM_001290675.1 (1973 residues) under pathological conditions. Immunostaining for HA confirmed that Na_v_1.7‐HA indeed located in SMACs alongside Na_v_1.8 and TrkB in DRG neurons of *Scn9a^HA/+^* SNI mice at 6 weeks post‐injury (Figure 2A). Notably, HA immunostaining results demonstrated that Na_v_1.7 is widely expressed across all neuron sizes, with varying expression levels within each size category (Figure S5G,H), well in line with single‐cell sequencing data (https://linnarssonlab.org/drg/, https://ernforsgroup.shinyapps.io/macaquedrg/, https://ernforsluolabs.shinyapps.io/HumanDRG/, http://research‐pub.gene.com/XSpeciesDRGAtlas/#symbol/symbol/19.html, https://painseq.shinyapps.io/publish/#) [35, 36, 37].

Given that low‐threshold mechanoreceptor TrkB DRG neurons expressing full‐length TrkB isoform play an important role in mechanical allodynia [38, 39, 40], we investigated whether these neurons develop Na_v_1.7 and Na_v_1.8 SMACs following injury. To address this, we engineered a *TrkB^2A‐Tomato/+^* reporter mouse line by substituting the stop codon in the final exon of the *Trkb* gene with a 2A‐Tomato cassette (Figure S6A–D). Immunostaining against Tomato and TUJ1 showed that Tomato labelled 10.9% ± 3.0% neurons in *TrkB^2A‐Tomato/+^* at P0 (Figure S6E,F), consistent with previous data [41]. Moreover, Tomato labelled more than 95% neurons containing TrkB in cytoplasmic in ipsilateral and contralateral L4‐5 DRG of adult sham and SNI *TrkB^2A‐Tomato/+^* mice (Figure S6G,H). Co‐immunostaining analysis unequivocally demonstrated the formation of Na_v_1.7/Na_v_1.8/TrkB SMACs in LTMR TrkB neurons (Figure 2B).

### 2.3 Development of SMACs in DRG Neurons of NP Patients

**FIGURE 3:**
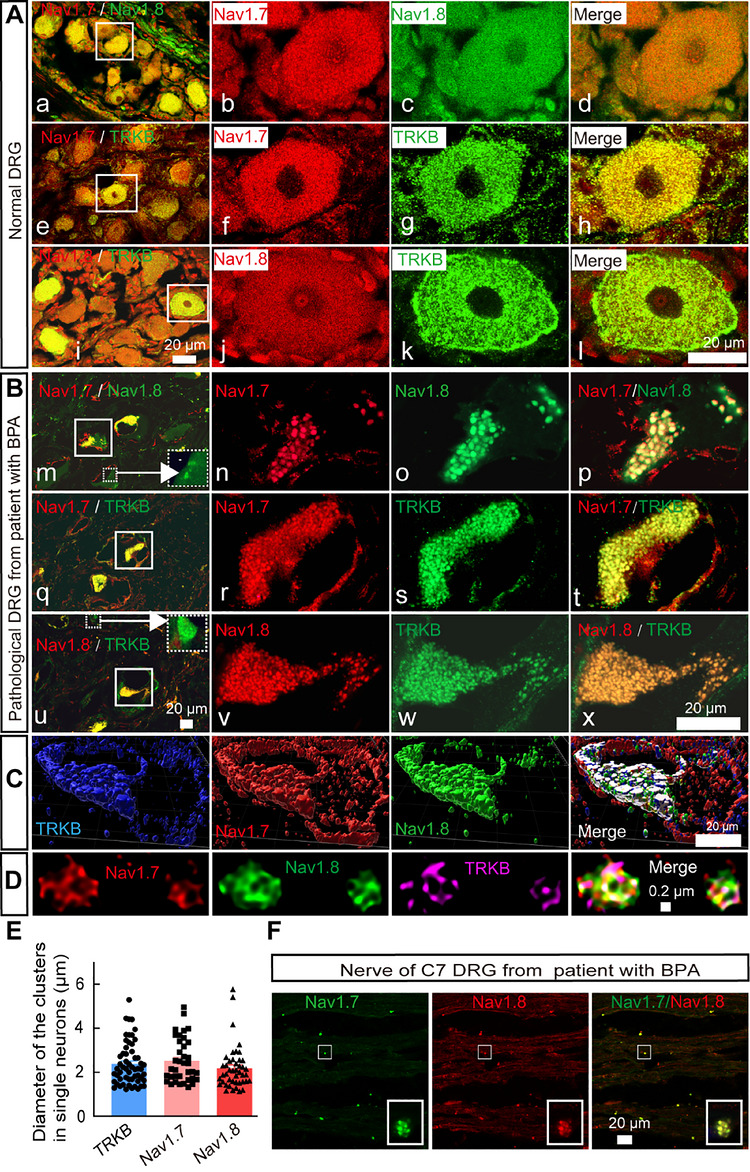
Formation of SMAC of Na_v_1.7, Na_v_1.8, and TRKB in DRG neurons from patients with NP due to brachial plexus avulsion (BPA). (A‐B) Immunostaining against Na_v_1.7 and Na_v_1.8 (a‐d, m‐p), Na_v_1.7 and TRKB (e‐h, q‐t) or Na_v_1.8 and TRKB (i‐l, u‐x) in DRG sections from naturally aborted human embryos (A) or patients (B). Arrows point to small clusters containing only Na_v_1.8 or only TRKB. (C) Three‐dimensional reconstruction of Na_v_1.7/Na_v_1.8/TRKB SMAC in DRG neurons from BPA patients with severe NP. (D) SIM images showed that Na_v_1.7, Na_v_1.8 and TRKB formed polygonal lattice cluster. (E) Diameter of clusters of TRKB, Na_v_1.7 or Na_v_1.8 in DRG neurons from 1 patient. (F) Immunostaining against Na_v_1.7 and Na_v_1.8 in DRG nerves from patients, revealing distribution of SMACs of Na_v_1.7 and Na_v_1.8 along nerve. Insets are high magnification view of the boxed region in corresponded images.The representative images were photographed from C7 avulsed root of a 57‐year‐old male BPA patient with the VAS scale of 8.

Based on these murine findings, we concluded that NP is associated with quantitative and qualitative alterations in Na_v_1.7 and Na_v_1.8 within DRG neurons. Prior to further validating this hypothesis, we aimed to corroborate these observations in human tissues. To this end, we performed immunostaining analysis on pathological DRG samples obtained from patients with brachial plexus avulsion (BPA) who had endured severe NP for a minimum of three months, comparing them with normal DRG tissue from 29‐week human embryos. In normal DRG, some TRKB^+^/Na_v_1.7^+^ neurons exhibited low expression of Na_v_1.8 (Figure S7A–D). Contrastingly, in the pathological DRG, a majority of TRKB^+^/Na_v_1.7^+^ neurons showed high expression of Na_v_1.8 (Figure S7E–H). Quantitative analysis demonstrated that both the number of neurons with high expression of Na_v_1.7, Na_v_1.8, and TRKB and the number of Na_v_1.7^+^/Na_v_1.8^+^/TRKB^+^ neurons were significantly higher in pathological DRG than in normal DRG (Figure S8A–H). The expression levels of Na_v_1.7, Na_v_1.8, and TRKB were 1.3‐, 1.6‐, and 1.6‐fold higher, respectively, in pathological DRG compared to normal DRG (Figure S8I). Consistent with murine observations, the majority of DRG neurons in severe NP patients harbored large SMACs comprised of Na_v_1.7, Na_v_1.8, and TRKB, a phenomenon notably absent in normal DRG; and small fractions of Na_v_1.7/Na_v_1.8 SMACs were localized on the plasma membrane (Figure 3A–C). Moreover, TRKB/Na_v_1.7 SMAC were present across all neuron sizes (diameter range: 30–120 µm), with over 73% of those with diameters >60 µm were classified as large neurons according to reported scale [42] (Figure S9A,B). SIM images showed that Na_v_1.7/Na_v_1.8/TRKB also formed the polygonal structure measuring approximately 160–250 nm in side length in SMAC of DRG neurons from patients (Figure 3D and Figure S10A–H). These SMACs contained tens to hundreds of polygonal lattice clusters (some appearing pentagonal or hexagonal), with an average diameter of 2 µm. Notably, a small subset of these clusters lacked Na_v_1.7, and contained only Na_v_1.8 or TrkB (Figure 3B and Video S1). Furthermore, SMACs encapsulating Na_v_1.7 and Na_v_1.8 were widely distributed along the DRG nerve of patients with severe NP (Figure 3F). Thus, this congruence between murine models and human patient data suggests SMACs as a pathological hallmark of severe chronic NP.

### 2.4 Combination of Na_v_1.7 and Na_v_1.8 Blockers Synergistically Relieves NP in Mouse Models

**FIGURE 4:**
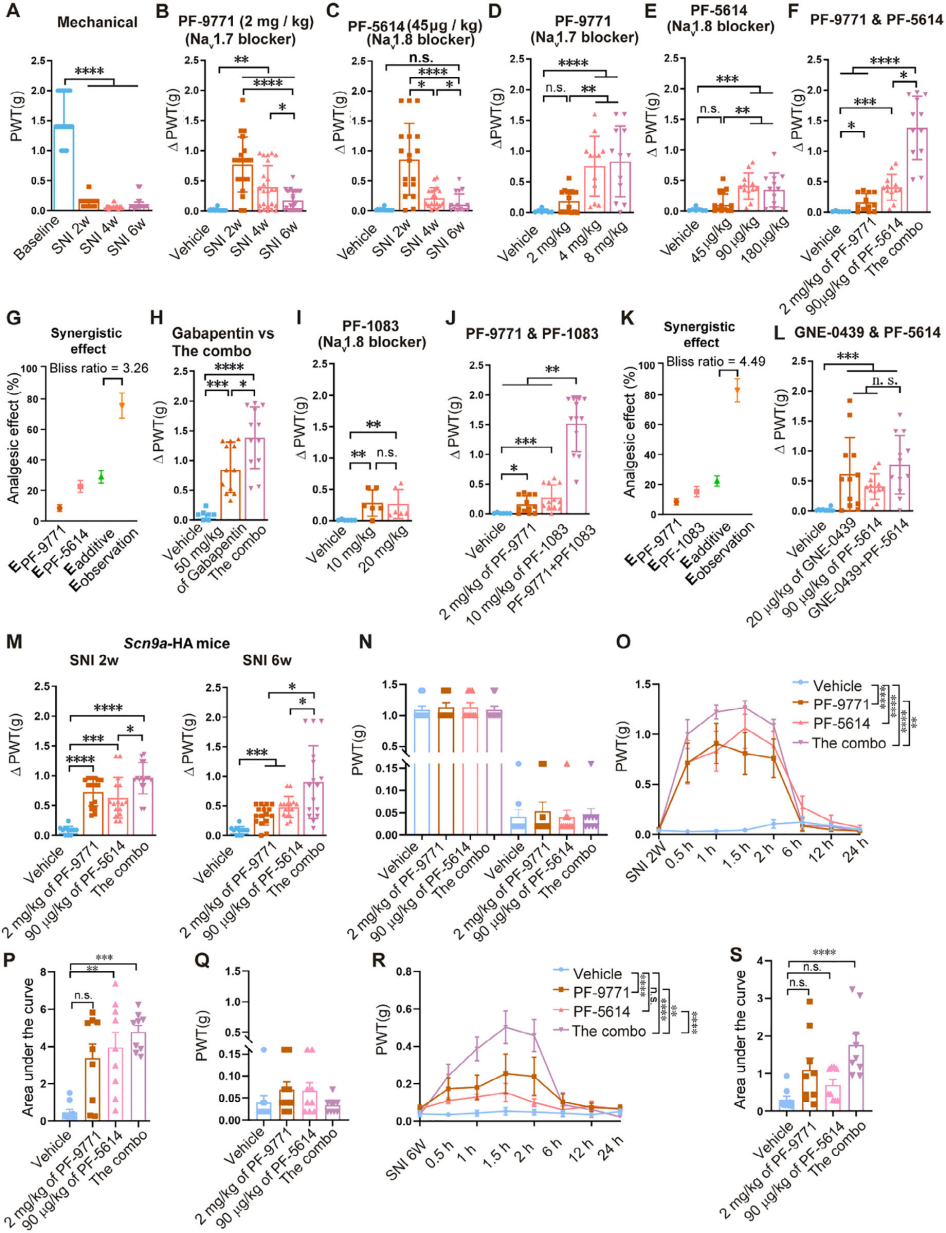
Mechanical allodynia in SNI models is mitigated more effectively by dual inhibition of Na_v_1.7 and Na_v_1.8 than by single inhibition. (A) Paw withdrawal threshold (PWT) in mice before injury and at the indicated weeks after SNI. Results are shown for 12 animals in each condition, and compared using the Mann‐Whitney test. Effect size = 0.3 (B–E) Change in paw withdrawal threshold (ΔPWT = PWTpost‐treatment—PWTpre‐treatment) in SNI mice after treatment with vehicle, PF‐05089771 or PF‐04885614 at the indicated doses and time points (D‐E: 6 weeks post‐injury). Data are shown for 20–28 animals per condition (panel B, effect size = 0.4), 17–21 animals (panel C, effect size = 0.4), or 9–16 animals (panels D‐E, effect size = 0.5). (F) Comparison of 2 mg/kg PF‐05089771 and 90 µg/kg PF‐04885614, alone or combined, at 6 weeks post‐injury (*n* = 12 animals per condition, effect size = 0.5). (G) Combined administration of 2 mg/kg PF‑05089771 and 90 µg/kg PF‐04885614 showed a significant synergistic analgesic effect (Bliss ratio = 3.26). Data are mean ± SEM. (H) The combo injection showed greater analgesic effects than 50 mg / kg of Gabapentin (n = 6 animals per condition, effect size = 0.5). (I–J) PF‐04531083 (I, n = 12 animals per condition, effect size = 0.7), or the combination of PF‐04531083 and PF‐05089771 (J, n =16 animals per condition, effect size = 0.5) in SNI mice at 6 weeks post‐injury. (K) Combined administration of 2 mg/kg PF‐05089771 and 10 mg/kg PF‐04531083 showed a significant synergistic analgesic effect (Bliss ratio = 4.49). Data are mean ± SEM. (L) The combination of 20 µg/kg GNE‐0439 and 90 µg/kg PF‐04885614 did not further alleviate NP in SNI mice at 6 weeks post‐injury than the same dose of GNE‐0439 and the same dose of PF‐04885614 (*n* = 12, effect size = 0.5) (M) Efficacy of the combination of PF‐05089771 and PF‐04885614 in *Scn9a^HA/+^* SNI mice at the indicated weeks post‐injury (*n* = 16, effect size = 0.4). (N) PWT measurements before SNI surgery (4 bars on left) and before drug administration at 2 weeks post‐injury. (O) Time‐course analysis of PWT following administration of 2 mg/kg PF‐05089771 and 90 µg/kg of PF‐04885614, alone or the combination, or vehicle in SNI mice at 2 weeks post‐injury. (P) Corresponding area under the curve analysis for the treatments shown in panel O. (Q) PWT measurements at 6 weeks post‐injury before drug administration. (R) Therapeutic efficacy of 2 mg/kg PF‐05089771 and 90 µg/kg of PF‐04885614, alone or combined in SNI mice at 6 weeks post‐injury. (S) Area under the curve analysis for the treatments shown in panel R. N‐S, *n* = 9 animals per group. Effect size = 0.6 (P‐S). n.s.: not significant, * *p* < 0.05, ** *p* < 0.01, *** *p* < 0.001, **** *p* < 0.0001 by Mann‐Whitney test or one‐way ANOVA with Tukey's test.

Both murine and human studies suggest that Na_v_1.7 and Na_v_1.8 function coordinately within SMACs to exacerbate NP, highlighting the necessity of concurrently targeting channels for effective pain relief. Notably, the analgesic efficacy of either the Na_v_1.7 blocker PF‐05089771 [22] or the Na_v_1.8 blocker PF‐04885614 [43] in alleviating mechanical allodynia in SNI mice exhibited a significant decline between 2 and 6 weeks post‐injury (Figure 4A–C), which was correlated to the formation of SMACs from 2 to 6 weeks post injury. Even at escalated doses, neither the Na_v_1.7 nor the Na_v_1.8 blocker substantially ameliorates NP in all tested mice by 6 weeks post‐injury (Figure 4D,E). In contrast, combining PF‐05089771 and PF‐04885614 (hereafter called “combination”) achieved excellent efficacy in all tested 12 mice even at 6 weeks post‐injury, surpassing the efficacy of the same doses of either inhibitor alone. Further analysis using Bliss model [44] demonstrated that the observed analgesic effect of combo was much greater than the additive effect, indicating synergistic effects of the Na_v_1.7 and the Na_v_1.8 blocker (Figure 4F,G; Figure S11A–C and Videos S5 and S6). Moreover, this combination was more effective than Gabapentin (Figure 4H), which caused sedation in some mice at dose of 100 mg/kg (data not shown). Additionally, we discovered that combining PF‐05089771 with another Na_v_1.8 blocker, PF‐04531083 [45], also demonstrated strong synergistic analgesic efficacy at 6 weeks post‐injury (Figure 4I–K). Conversely, the combination of Na_v_1.7 blocker GNE‐0439 [46] with PF‐04885614 did not prove more effective than GNE‐0439 alone (Figure 4L and Figure S11D,E). Furthermore, combining PF‐05089771 with PF‐04885614 also significantly relieved NP in *Scn9a* ^HA/+^ SNI mice at 6 weeks post‐injury, better than either blocker alone (Figure 4M).

To evaluate the time course of pain relief following drug administration, we compared the combination therapy with PF‐05089771 or PF‐04885614 monotherapy over 24 h in SNI mice at 2 and 6 weeks post‐injury. Before drug treatment, SNI mice exhibited severely reduced mechanical pain thresholds (as low as 0.02 g) at both time points. At 2 weeks post‐injury, both the combination and single‐drug treatments significantly alleviated NP, as shown in the time‐course curves (Figure 4N,O). However, the area under the curve (AUC) for PF‐05089771 was not significantly higher than that of the vehicle group, due to a weak response in some mice (Figure 4P). By 6 weeks post‐injury, only the combination therapy produced a significantly greater AUC compared to the vehicle group (Figure 4Q–S).

### 2.5 SMACs of Na_v_1.7 and Na_v_1.8 Contribute to Diabetic NP

**FIGURE 5:**
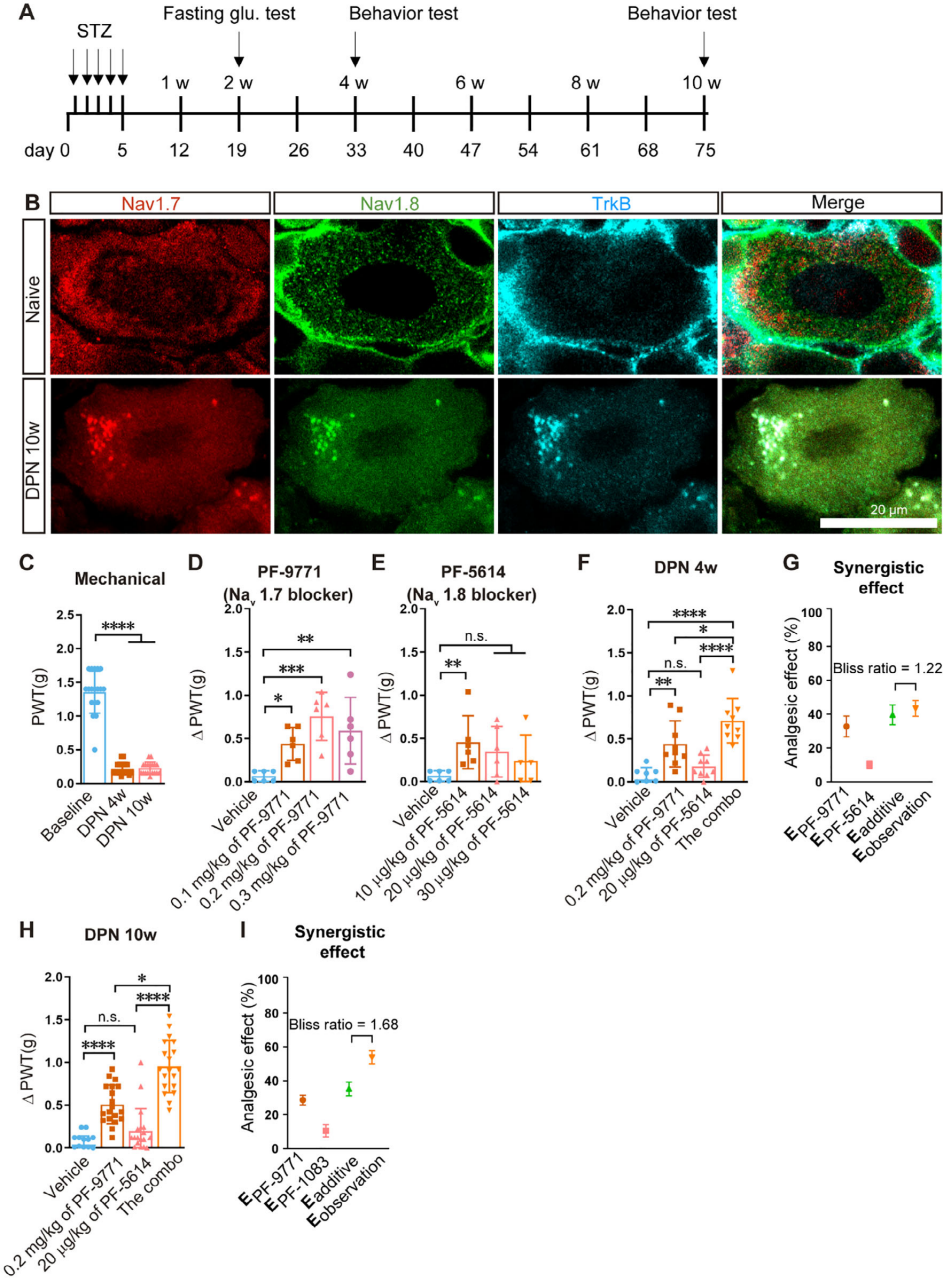
Na_v_1.7 and Na_v_1.8 SMACs contribute to diabetic neuropathic pain in mice. (A) Schematic of the generation of a mouse model of chronic diabetic neuropathy. (B) Immunostaining against Na_v_1.7, Na_v_1.8 and TrkB in DRG sections from naïve (upper panel) or diabetic mice (lower panel, DPN: Diabetic Peripheral Neuropathy). (C) PWT of diabetic mice at the indicated weeks after STZ treatment (*n* = 18 animals per condition, effect size = 0.8). (D,E) The dosage effect of PF‐05089771 (D, effect size = 0.7) and PF‐04885614 (E, effect size = 0.8) in diabetic mice at 4 weeks (*n* = 6 animals per condition). (F–I) Synergistic analgesic efficacy of the combination comprising higher doses of PF‐05089771 (0.2 mg/kg) and PF‐04885614 (20 µg/kg) in diabetic mice at 4 weeks (F,G, *n* = 9 animals per condition, effect size = 0.6) or 10 weeks after final injection of STZ (H‐I, *n* = 19 animals per condition, effect size = 0.4). * *p* < 0.05, ** *p* < 0.01, *** *p* < 0.001, **** *p* < 0.0001 by Kruskal‐Wallis test (C‐E and H) or one‐way ANOVA with Tukey's test (F).

Given that NP is a common complication of diabetes [23], we investigated whether SMACs containing Na_v_1.7 and Na_v_1.8 might also play a role in NP under diabetic conditions. To explore this, we established a diabetic mouse model through intraperitoneal administration of streptozocin (STZ). The model was deemed successful when the fasting blood glucose level exceeded 13 mm at 2 weeks following the final STZ injection (Figure 5A and Figure S12A). Immunostaining against Na_v_1.7, Na_v_1.8, and TrkB revealed a significant increase in the number of DRG neurons expressing Na_v_1.7 and Na_v_1.8 and co‐expressing both channels in diabetic mice compared to naïve mice (Figure S12B,C). Consistently, the overall protein expression level of Na_v_1.7 or Na_v_1.8 within the L4‐6 DRG lysate only showed an increase trend in diabetic mice at 10 weeks after the last STZ injection (Figure S12D). These data suggest that the increased expression of Na_v_1.7 and Na_v_1.8 in DRGs contributes to mechanical allodynia in diabetic mice. More importantly, we observed that large Na_v_1.7/Na_v_1.8/TrkB SMACs presented in DRG neurons of STZ‐induced diabetic mice at 10 weeks after the final STZ injection, not in control DRG from naïve mice (Figure 5B). Diabetic mice exhibited signs of mechanical pain by 4 weeks after the final STZ injection, with the low mechanical pain threshold persisting at least till 10 weeks (Figure 5C). The combination at dose of 0.2 mg/kg PF‐05089771 and 20 µg/kg PF‐04885614 synergistically relieved NP in diabetic mice at both 4 and 10 weeks after the last STZ injection, outperforming either inhibitor alone (Figure 5D–I and Figure S12E–G).

### 2.6 Activation of TrkB Promotes SMACs Formation

**FIGURE 6:**
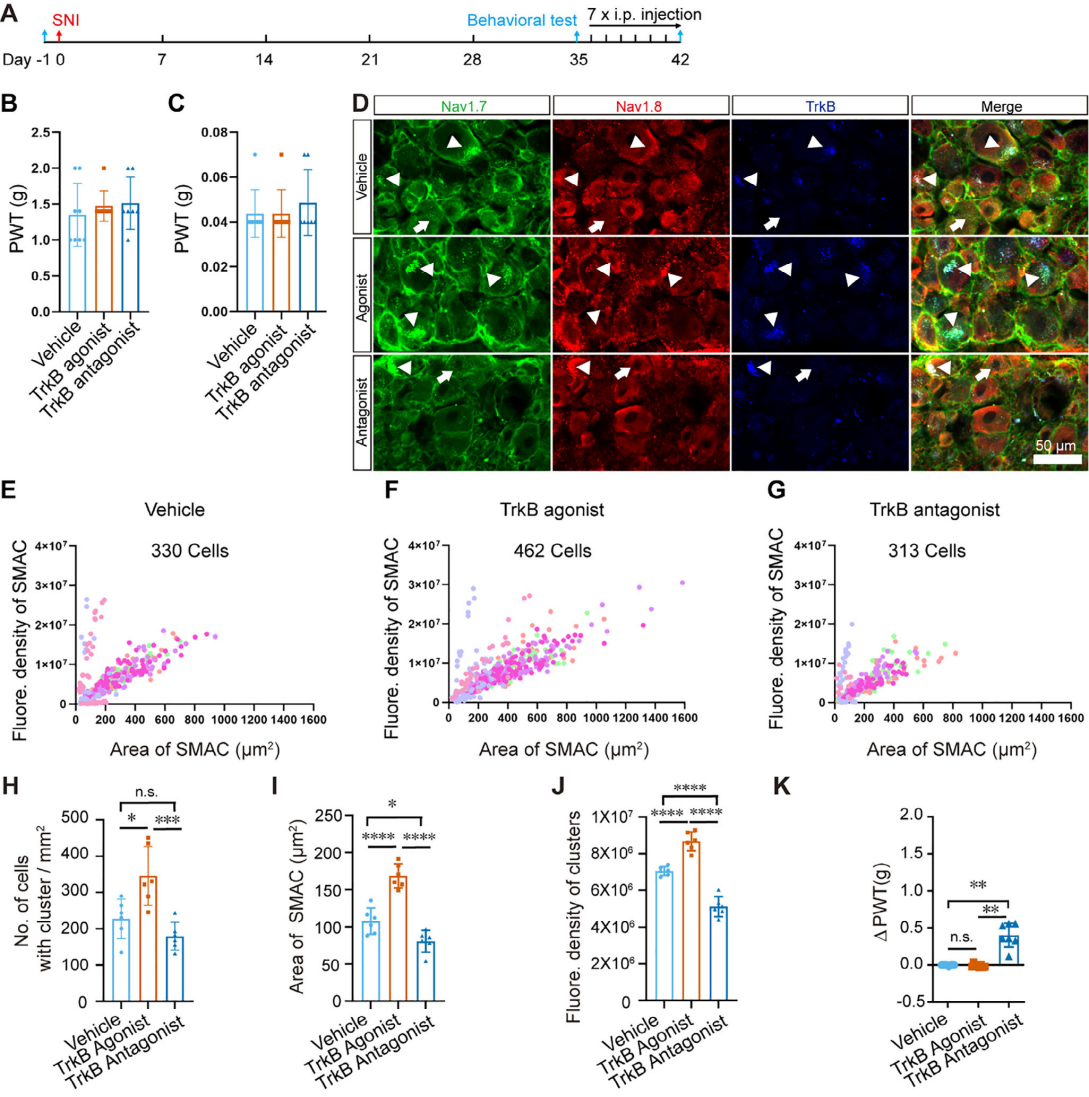
Activation of TrkB promotes SMAC formation. (A) A schematic of experimental procedure. TrkB agonist (7, 8‐Dihydroxyflavone) or antagonist (ANA‐12) or vehicle was administered daily for 7 days in the last week. (B, C) The paw withdrawal threshold of vehicle (*n* = 8), TrkB agonist (*n* = 8) or TrkB antagonist (*n* = 7) treated mice before (B, baseline) and 5 weeks post (C, before drug treatment) SNI operation. (D) Immunostaining against Na_v_1.7, Na_v_1.8, and TrkB in DRG sections from vehicle‐treated (upper panel), TrkB agonist‐treated (middle panel) and TrkB antagonist‐treated SNI mice (lower panel) at 6 weeks post‐injury. Arrowheads point to SMAC of Na_v_1.7/Na_v_1.8/TrkB, Arrows point to cluster containing Na_v_1.7, not Na_v_1.8/TrkB. (E–G) The fluorescence density and area of SMAC in L5 DRG neurons from SNI mice treated with vehicle (E), TrkB agonist (F), or TrkB antagonist (G) at the 6 weeks post‐injury. Each dot represents one cell, while dots with same color represents cells derived from same animal. *n* = 6 animals per group. (H) Numbers of cells containing cluster of Na_v_1.7, Na_v_1.8, and TrkB. (I, J) Area (I) and fluorescence density (J) of SMAC. (K) Change in paw withdrawal threshold in SNI mice 1 h after last treatment with vehicle, TrkB agonist or antagonist, effect size = 0.7. * *p* < 0.05, ** *p* < 0.01, *** *p* < 0.001 by Kruskal‐Wallis test (B,C) or the ANOVA test with Tukey's test (H‐J) or Dunn's multiple comparisons test (K, *n* = 6–8).

To explore the potential role of TrkB in the formation of SMACs, we conducted behavioral assessments on SNI mice with a C57BL/6 genetic background at 35 days post‐injury. Following these tests, the mice were randomly allocated into three groups and subjected to a 7‐day treatment protocol. This regimen involved daily intraperitoneal injections of either a vehicle, a TrkB agonist (7, 8‐Dihydroxyflavone), or a TrkB antagonist (ANA‐12) (Figure 6A–C). Compared to vehicle treated mice, TrkB agonist significantly increased the number of DRG neurons exhibiting Na_v_1.7/Na_v_1.8/TrkB cluster, expanded the area of SMACs, and enhanced the density of these clusters. Conversely, the TrkB antagonist significantly decreased the area of SMACs and the density of clusters (Figure 6D–J). Aligning with TrkB antagonist's capacity to inhibit SMACs growth, mechanical pain in SNI mice showed significant partial alleviation 1 h after final injection. Vice versa, TrkB agonist substantially promoted SMACs growth and lowered mechanical threshold in 5 out of 8 SNI mice, it did not significantly reduce the overall mechanical threshold, possibly due to the already minimized threshold by SNI (Figure 6K).

### 2.7 Cytoskeletal Proteins Promote Formation of SMACs Under Pathologic Condition

**FIGURE 7:**
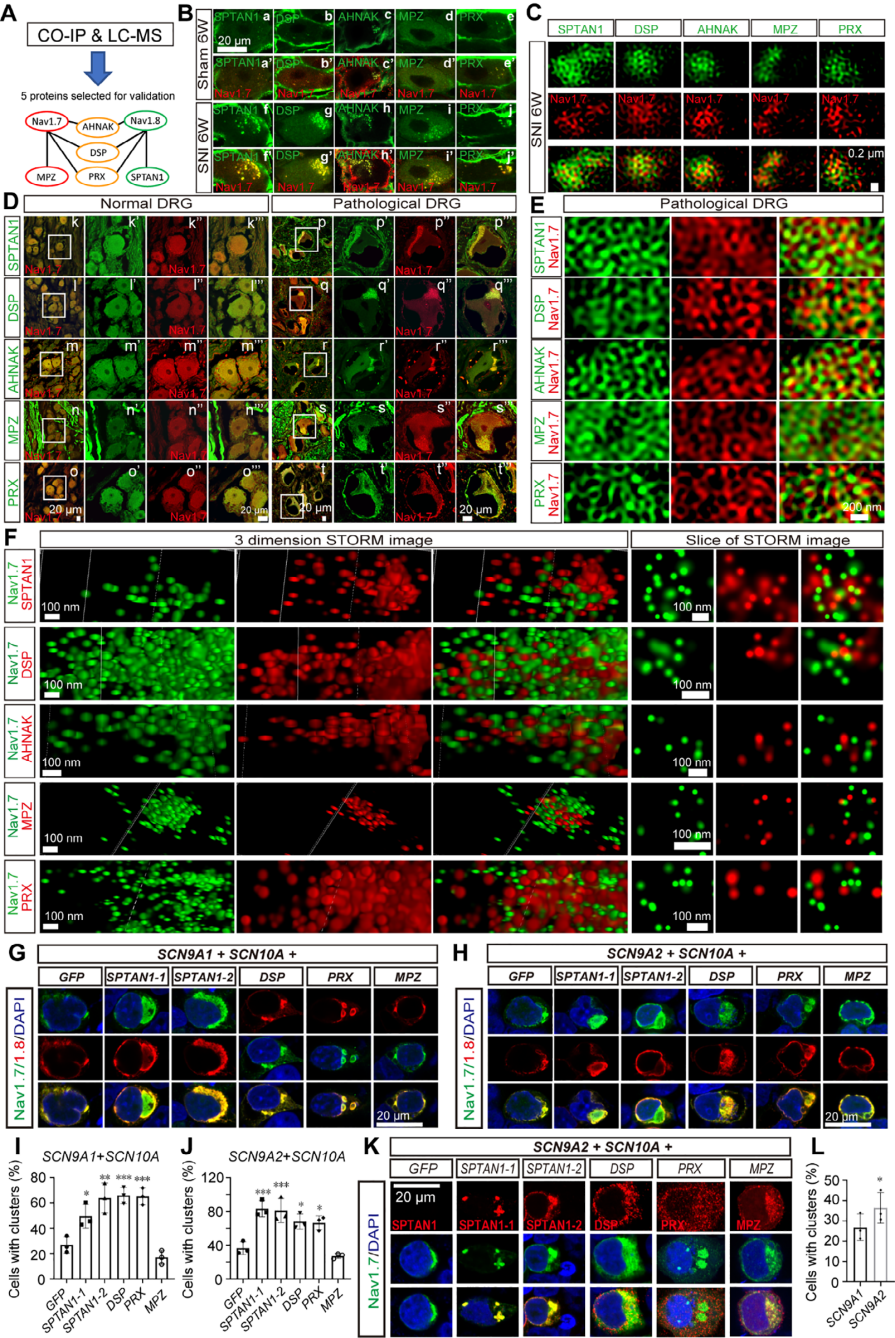
The cytoskeletal proteins promote the clustering of Na_v_1.7 and Na_v_1.8. (A) Selected five proteins that were co‐immunoprecipitated by antibodies against Na_v_1.7 or Na_v_1.8 and identified by mass spectrometry. (B) Immunostaining against Na_v_1.7 (a’‐e’, f’‐j’), SPTAN1 (a, a’, f, f’), DSP (b, b’, g, g’), AHNAK (c, c’, h, h’), MPZ (d, d’, i, i’) or PRX (e, e’, j, j’) in DRG sections from mice subjected to sham surgery (a‐e) or SNI 6 weeks previously (f‐j). (C) SIM images of cluster immunostained with antibodies against Na_v_1.7 (red) and SPTAN1, DSP, AHNAK, MPZ or PRX (green) in DRG sections of SNI mice at 6 weeks post‐injury. (D) Immunostaining against the proteins indicated at the *far left* in DRG sections from naturally aborted human embryos (k‐o) or from patients with NP‐induced by BPA (p‐t). (E) SIM images of cluster immunostained with antibodies against Na_v_1.7 (red) and SPTAN1, DSP, AHNAK, MPZ or PRX (green) in DRG sections of BPA patients. (F) 3D (Left panel) and one slice (Right panel) view of STORM images of cluster immunostained with antibodies against Na_v_1.7 and SPTAN1, DSP, AHNAK, MPZ or PRX in primary cultured DRG neurons from SNI mice at 6 weeks post‐injury. (G,H) Immunostaining against Na_v_1.7 and Na_v_1.8 in cultures of HEK293 cells co‐transfected with plasmids expressing *SCN10A* and the shorter (*SCN9A1*) or longer (*SCN9A2*) isoform of *SCN9A* (see Methods), as well as with plasmid expressing one of the five potential interactors SPTAN1 (Two isoforms of SPTAN1 were tested, as described in Methods.), DSP, PRX or MPZ. Control cells expressed green fluorescent protein (GFP) without any potential interactor. (I,J) Percentages of cells containing clusters of Na_v_1.7 and Na_v_1.8 among cells that were co‐transfected with the (I) shorter or (J) longer isoform of *SCN9A*, *SCN10A* as well as one of the four potential interactors. (K) Immunostaining of co‐transfected cells showing overlap of Na_v_1.7 with SPTAN1 (either isoform), DSP, PRX, and MPZ. Cells were counterstained with DAPI. (L) Percentages of cells containing clusters of Na_v_1.7 and Na_v_1.8 among cells that were co‐transfected with the shorter or longer isoform of *SCN9A*, *SCN10A*, and *GFP*. * *p* < 0.05, ** *p* < 0.01, *** *p* < 0.001, **** *p* < 0.001 by one‐way ANOVA test or Student's *t* test (I, J, and L, *n* = 3 independent experiments).

To identify potential proteins that facilitate the formation of Na_v_1.7/Na_v_1.8 SMACs, which could serve as therapeutic targets for NP, we conducted a series of experiments. Using antibodies specific to Na_v_1.7 and Na_v_1.8, we immunoprecipitated proteins from the L4‐6 DRG of SNI mice at 6 weeks post‐injury. These proteins were subsequently identified by Mass Spectrometry. In two independent experiments, we identified several proteins whose molecular weights corresponded to the bands observed in SDS‐polyacrylamide gels, suggesting their potential involvement in the assembly of Na_v_1.7/Na_v_1.8 SMACs (Figures S13A–C and S14A and Tables S2–S5). The network analysis of co‐immunoprecipitated proteins with Na_v_1.7 and Na_v_1.8 showed that some of them may interact with these channels (Figure S14B), and we also noticed 11 proteins whose mRNA expression in L4‐6 DRG was altered by nerve injury in mice [39]: *Sptan1*, *Dsp*, *Prx*, *Mpz*, *Ahnak*, *Thbs2*, *Gnmt*, *Ass1*, *Aldh1l1*, *Anxa1*, and *Rpsa*. Based on these results, we selected cytoskeletal proteins SPTAN1, DSP, PRX, MPZ, and AHNAK for further validation (Figure 7A), and confirmed their co‐localization with Na_v_1.7 in the SMACs of DRG neurons from SNI mice (Figure 7B). Sham animals, in contrast, showed occasional co‐localization of Na_v_1.7 with SPTAN1, DSP, AHNAK, or PRX, but not MPZ, in small clusters (Figure 7B). SIM images unveiled that these five proteins closely intermingled with Na_v_1.7 and also formed polygonal lattice clusters by themselves (Figure 7C and Figures S15–S17). These cytoskeletal proteins linked clusters of Na_v_1.7 to form SMACs in mouse DRG from SNI mice 6 weeks post‐injury (Figures S15–S17). Similarly, these five proteins co‐localized with Na_v_1.7 in SMACs in pathological DRG from patients with BPA‐induced NP, but not in normal DRG (Figure 7D), and they formed distinct polygonal lattice clusters and grouped clusters as an entity SMAC (Figure 7E and Figures S18–S20), especially, images acquired and reconstructed from both Zeiss and HIS‐SIM clearly demonstrated that SPTAN1 linked Na_v_1.7 clusters together to form an entity SMAC (Figure S18). Further insights derived from STORM images revealed that these five proteins formed columnar clusters exhibiting a gradient density distribution. Within these structures, Na_v_1.7 was observed to be closely intermingled with the proteins in briefly cultured DRG neurons from SNI mice (Figure 7F; Figure S21A–O and Videos S7–S11).

To directly assess the involvement of SPTAN1, DSP, PRX, and MPZ (excepting of AHNAK due to its prohibitive size for overexpression, 5656 residues) in the assembly of Na_v_1.7/Na_v_1.8 SMACs, we conducted overexpression experiments in HEK293 cells. These cells were transfected with plasmids expressing one of these proteins together with plasmids expressing Na_v_1.7 and/or Na_v_1.8. Specifically, we explored two isoforms of SPTAN1, one containing 2498 residues (ENST00000630866.2, named SPTAN1‐1) and another containing 2452 residues (ENST00000358161.9, called SPTAN1‐2). Additionally, we examined two Na_v_1.7 isoforms: SCN9A, with 1977 residues (NM_002977), and SCN9A2 (ENST00000303354.11), which includes an extra sequence of 11 residues (VIIDKATSDDS) at positions 648–658, their orthologous transcripts are predominantly expressed in mouse DRG [39]. Our results demonstrated that the Na_v_1.7 antibody and Na_v_1.8 antibody don't cross‐react with Na_v_1.8/Na_v_1.9 and Na_v_1.7/Na_v_1.9 in the overexpression system (Figure S22A). Our findings revealed that overexpressing SPTAN1‐1, SPTAN1‐2, DSP, PRX or MPZ individually facilitated the clustering of both Na_v_1.7 and Na_v_1.8. Furthermore, co‐expressing all five proteins promoted formation of large Na_v_1.7 and Na_v_1.8 clusters (Figure S22B–E), while the numbers of cells containing Na_v_1.7/Na_v_1.8 clusters increased when the two channels were co‐expressed with any of the four proteins, except MPZ (Figure 7G–J). SPTAN1‐1 and SPTAN1‐2 co‐localized with Na_v_1.7 in SMACs, while DSP, PRX, and MPZ clustered and partially overlapped with Na_v_1.7 (Figure 7K). Intriguingly, without overexpression of DSP, PRX, MPZ, and SPTAN1, the longer Na_v_1.7 (SCN9A2) isoform more readily formed clusters with Na_v_1.8 compared to its shorter isoform (SCN9A1) (Figure 7L), suggesting that the additional 11 residues in SCN9A2 might strengthen interactions with Na_v_1.8 and/or other components of SMACs.

**FIGURE 8:**
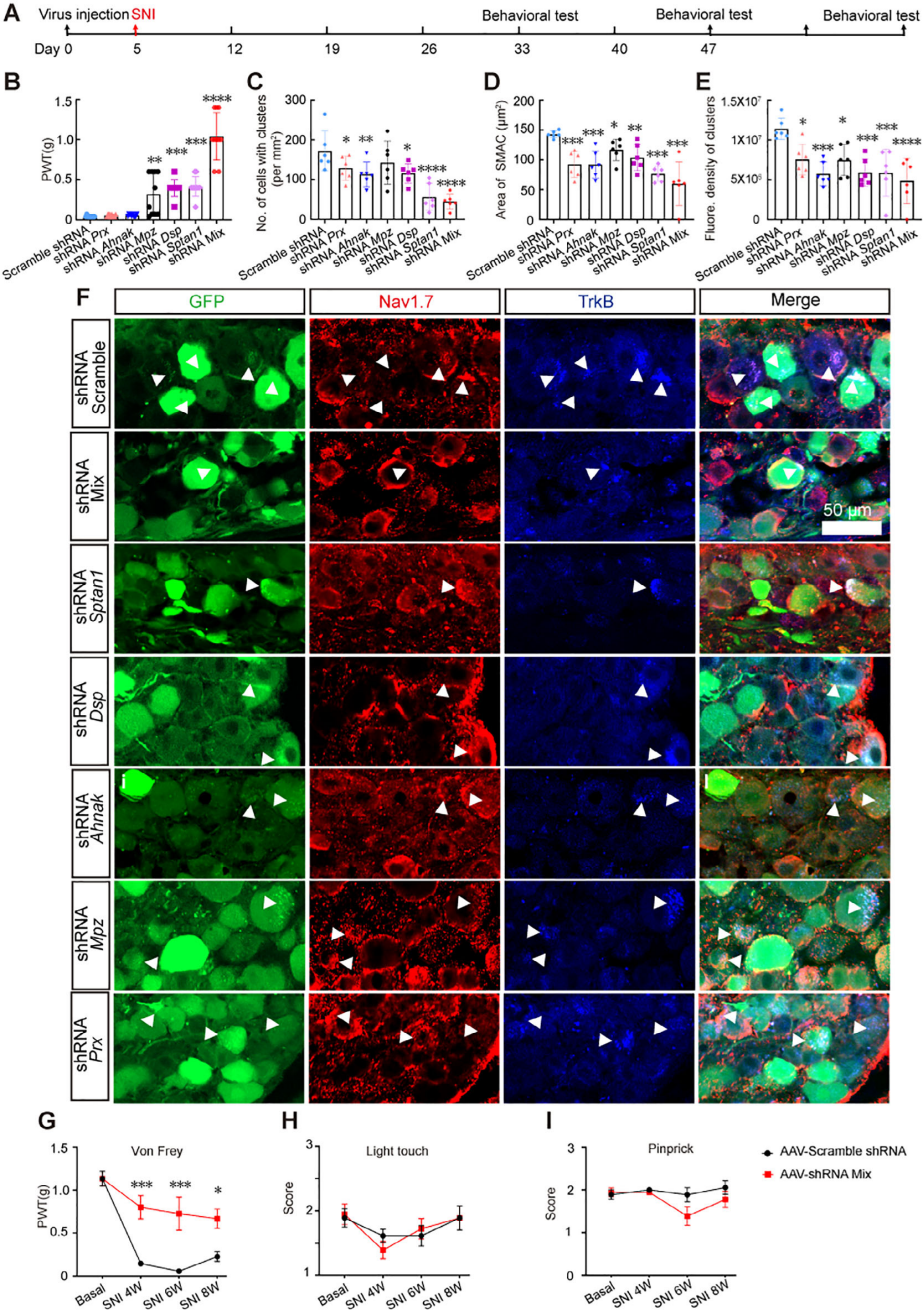
Targeting cytoskeletal proteins inhibits SMACs formation and relieves NP. (A) Time course of knock‐down experiment using shRNA‐expressing AAV virus. (B) The PWT of SNI mice with DRG injection of AAV viruses expressing either scrambled shRNA, or shRNA targeting *Prx*, *Ahnak*, *Mpz*, *Dsp*, *Sptan1*, or all of the 5 shRNAs (mix). (C–E) Number of DRG neurons with TrkB/Na_v_1.7 clusters (C) and area of SMAC (D), and fluorescence density of clusters (E) in L5 DRG of SNI mice with DRG injection of AAV viruses expressing either scrambled shRNA, or shRNA targeting *Prx*, *Ahnak*, *Mpz*, *Dsp*, *Sptan1*, or all of the 5 shRNAs (mix). (F) Immunostaining against GFP (Green), Na_v_1.7 (Red), and TrkB (Blue) in L5 DRG section from SNI mice with DRG injection of scrambled shRNA‐expressing, shRNA *Sptan1*‐expressing, shRNA *Dsp*‐expressing, shRNA *Ahnak*‐expressing, shRNA *Mpz*‐expressing, shRNA *Prx*‐expressing, or mixture of 5 shRNA‐expressing AAV viruses. Arrowheads point to neurons with SMAC. (G‐I) Time‐course analysis of PWT in SNI mice with injection of AAV‐ scrambled shRNA or AAV‐shRNAs (mix) from 4 to 8 weeks post‐injury, assessed using von Frey filament stimulation (G), soft brush stimulation (H), or pinprick testing (I), *n* = 6 animals per group, effect size = 0.9 (G–I). * *p* < 0.05, ** *p* < 0.01, *** *p* < 0.001, **** *p* < 0.001 by Kruskal‐Wallis test (B, effect size = 0.5) or unpaired Student's *t* test (C–E). *n* = 10 in B, *n* = 6 in C–E.

To determine whether the knock‐down of SPTAN1, DSP, AHNAK, MPZ, or PRX could inhibit the formation of SMAC and the progression of severe NP, we conducted a targeted intervention. The shRNAs targeting these five proteins were screened in HEK293 cells with overexpressing the targeted cDNA fragment of each gene and the ShRNAs with more than 70% knockdown efficiency were cloned in AAV vector (Figure S23A–E and Table S6). Further the knockdown efficiency of each AAV‐shRNA was validated by injecting into DRG of *WT* mice (Figure S23F–K). Five days prior to the SNI surgery, we administered rated a viral mixture expressing shRNA targeting these five proteins into the L4 and L5 DRG of mice. This intervention effectively prevented the development of NP in all 10 examined mice. Additionally, it significantly reduced the number of neurons containing SMACs, the overall area of SMACs, and the density of Na_v_1.7 and TrkB within the clusters, compared to control SNI mice that received a scrambled shRNA‐expressing virus (Figure 8A–F; Figure S24A,B, and Videos S12 and S13). These findings suggest a critical role for these proteins in SMAC formation and NP progression. Knock‐down of each individual protein significantly reduced both the area of SMAC and the density of Na_v_1.7 and TrkB in the clusters (Figure 8D,E). Specifically, knocking down either SPTAN1 or DSP not only significantly reduced the generation of SMAC but also partially relieve NP, while knock‐down of AHNAK alone decreased the generation of SMAC, but did not significantly relieve NP (Figure 8B,C). In line with previous report that mutation of MPZ is associated with painful neuropathy [47, 48], knock‐down of MPZ alone resulted in a significant increase in mechanical pain threshold in SNI mice at 6 weeks post‐injury (Figure 8B). These results demonstrated that SPTAN1, DSP, and AHNAK substantially promote the formation of Na_v_1.7/Na_v_1.8 SMACs in vivo, and that MPZ and PRX also contribute to SMACs growth. To evaluate the long‐term efficacy and potential side effects of mixed siRNAs, we assessed mechanical pain (von Frey test), light touch sensitivity (brush test), and acute pain responses (pinprick test). The results showed that AAV‐mix‐shRNAs significantly alleviated NP from 2 to 8 weeks post‐injury (the final observation time point) compared to the AAV‐scramble control, without affecting other somatic sensory modalities (Figure 8G–I and Videos S14 and S15). These results indicate that AAV‐mix‐shRNAs induce sustained NP alleviation (persisting for at least 2 months) while preserving physiological sensation.

### 2.8 Selective Interactions Among Five Cytoskeletal Proteins Form the Scaffold of SMACs

**FIGURE 9:**
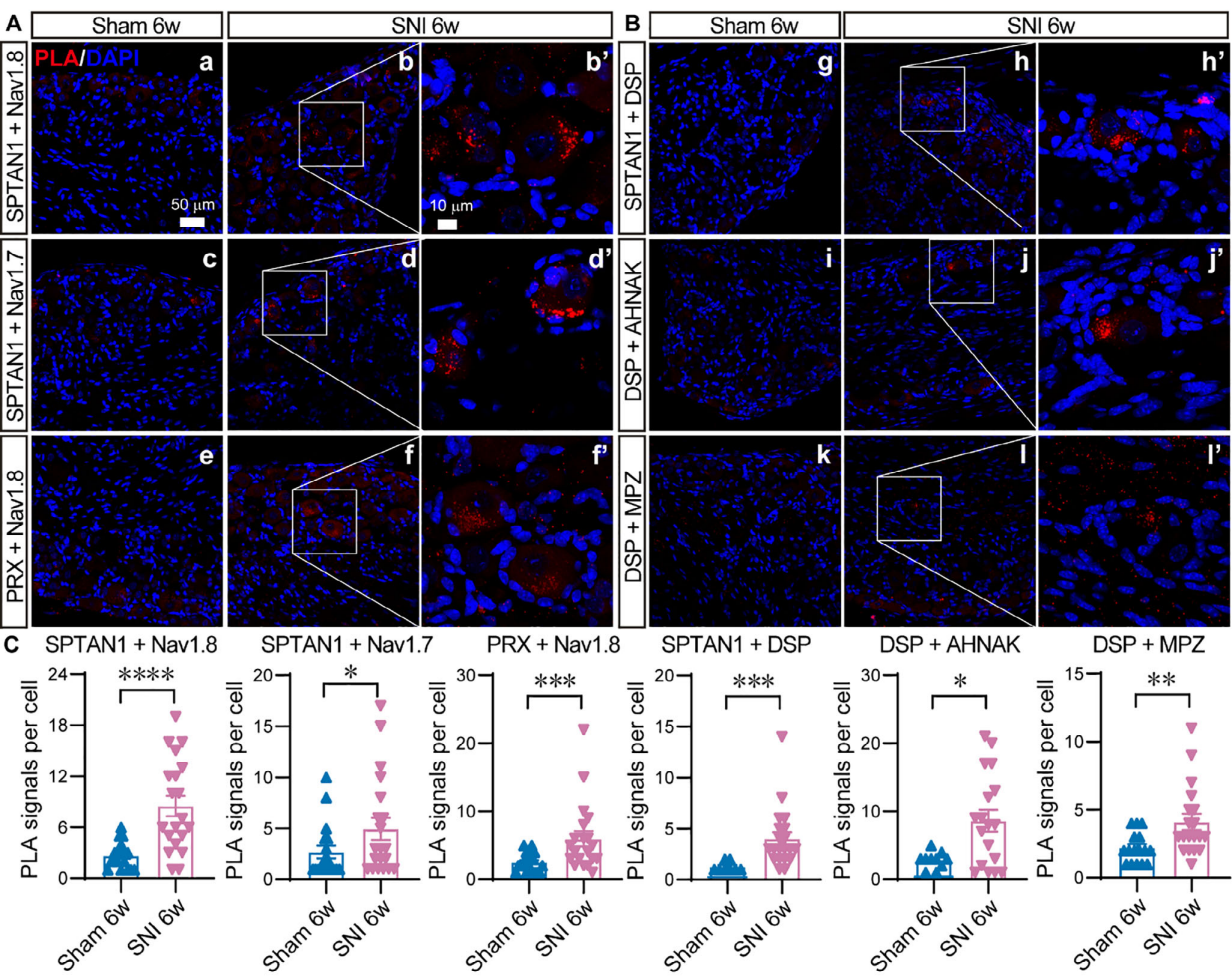
Proximity ligation assay (PLA) reveals direct interaction of Na_v_1.7/Na_v_1.8 and five cytoskeletal proteins. (A,B) The images illustrated PLA signals in DRG neurons from sham‐operated (a, c, e, g, i, k) and SNI‐operated (b, d, f, h, j, l) mice at 6 weeks post‐injury, b’, d’, f’ h’, j’, l’ are the high magnification view of the boxed region in b, d, f, h, j, l. (C) Analyzed data showed PLA signals per cell significantly increased in SNI mice, compared to sham‐operated controls. *n* = 20 cells from 3 animals per group. * *p* < 0.05, ** *p* < 0.01, *** *p* < 0.001, **** *p* < 0.0001, unpaired Student's *t* test.

To investigate potential direct interactions between Na_v_1.7/Na_v_1.8 and five cytoskeletal proteins, we performed protein‐protein docking using HADDOCK 2.4 [49] and predicted some potential interactions, and then we performed Proximity Ligation Assay (PLA) utilizing the L4‐5 DRGs from SNI and sham mice at 6 weeks post‐injury. Our findings unequivocally confirmed the direct interactions between Na_v_1.7/Na_v_1.8 and these cytoskeletal proteins, including Na_v_1.7/Na_v_1.8 with SPTAN1, and Na_v_1.8 with PRX. Interactions of DSP with SPTAN1, AHNAK, and MPZ were also observed (Figure 9A,B). Notably, the number of PLA signals per cell was significantly increased in DRG neurons derived from SNI mice compared to the sham controls at 6 weeks post‐injury (Figure 9C). These results strongly suggest that the interactions among Na_v_1.7/Na_v_1.8 and cytoskeletal proteins are enhanced under NP conditions. Collectively, our data support a model in which DSP interacts with SPTAN1, AHNAK, and MPZ to form a scaffold, while SPTAN1 further recruits Na_v_1.7/Na_v_1.8, and Na_v_1.8 associates with PRX, facilitating SMAC assembly.

### 2.9 TrkB/CREB Signaling Regulates the Expression of Four Cytoskeletal Proteins, and TrkB Interacts With DSP

**FIGURE 10:**
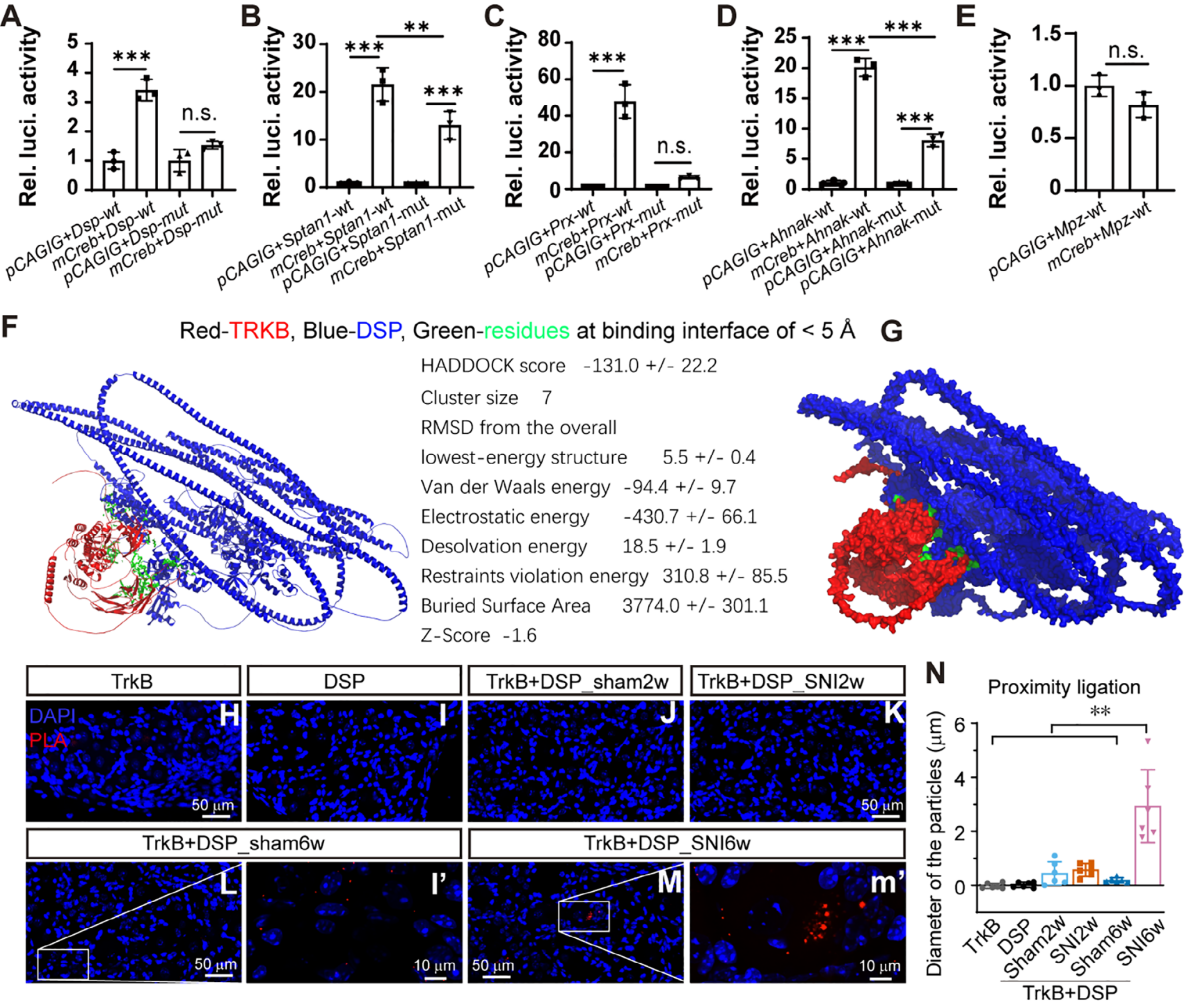
BDNF/TRKB/CREB signaling pathway activates expression of the four cytoskeletal proteins, and TRKB directly interacts with DSP. (A) CREB activated transcription of *Dsp*. Co‐transfection of *pCAG‐Creb* and *pGL4.17‐Dsp* into HEK293 cells significantly increased the activity of the *Dsp* promoter, driving firefly luciferase expression, compared with empty vector (*pCAGIG*) co‐transfected control (*left* two bars). However, co‐transfection of *pCAG‐Creb* didn't increase the activity of the promoter of *Dsp* with mutated CREB‐binding sites (*right* two bars). (B) CREB activated *Sptan1* transcription. Overexpression of CREB significantly enhanced the activity of the *Sptan1* promoter (*left* two bars), while the *Sptan1* promoter with mutated CREB‐binding sites significantly reduced the activation of CREB (*right* two bars). (C) CREB activated *Prx* transcription. Overexpression of CREB significantly enhanced the activity of the *Prx* promoter (*left* two bars), in contrast, mutation of the CREB‐binding sites in the promoter abolished this transcriptional activation (*right* two bars). (D) CREB activated *Ahnak* transcription. Overexpression of CREB significantly enhanced the activity of the *Ahnak* promoter (*left* two bars), while the *Ahnak* promoter with mutated CREB‐binding sites significantly reduced the activation of CREB (*right* two bars). (E) Co‐transfection of *pCAG‐Creb* and *pGL4.17‐Mpz* into HEK293 cells didn't increase the activity of the *Mpz* promoter, compared with empty vector (*pCAGIG*) co‐transfected control. (F,G) The interaction of TRKB (Red) and DSP (Blue) was predicted by HADDOCK (https://rascar.science.uu.nl/haddock2.4), and then the corresponding structure was downloaded and visualized using PyMOL. Residues at the predicted binding interface (within 5 Å of each other) were highlighted in Green. (H–N) PLA demonstrated that the direct interaction of TrkB and DSP significantly increased in DRG neurons from SNI mice at 6 weeks post‐injury, compared to sham‐operated controls. Data are shown in mean ± SEM, *n* = 3 independent technical replicates (A‐E) and *n* = 20 cells from 3 animals per group (N). n.s.: not significant, ** *p* < 0.01, *** *p* < 0.001, **** *p* < 0.0001 by one‐way ANOVA with Tukey's multiple comparisons (A–D) or unpaired Student's *t* test (E and N).

To investigate whether TrkB signaling directly regulates the expression of cytoskeletal proteins, we analyzed the 1 kilobase promoters of the five cytoskeletal proteins and found potential CREB‐binding sites (TGAC[G/T][T/C][A/G]) [50, 51] predicted by AnimalTFDB 4.0 [52]. Dual luciferase reporter assay demonstrated that overexpression of CREB significantly increased the activity of the cloned promoters of *Dsp*, *Sptan1*, *Prx*, and *Ahnak*, and this transcriptional activation was abolished or significantly reduced by the mutations in the CREB‐binding sites in the promoters (Figure 10A–E and Table S6). These findings indicate that BDNF/TrkB/CREB signaling pathway drives the upregulation of structural proteins of SMACs, thereby promoting SMACs formation in TrkB LTMR neurons.

Since TrkB co‐localizes with the cytoskeletal proteins in SMACs, we hypothesized that TrkB may directly interact with the structural proteins of SMACs. To test this, we performed protein‐protein docking using HADDOCK 2.4 [49] and predicted potential interactions between TRKB and the five cytoskeletal proteins. Among these, DSP exhibited the strongest predicted binding affinity, with the lowest docking score (‐131.0 ± 22.2) and a binding interface of <5 Å (Figure 10F,G). Furthermore, PLA confirmed a significant increase in TrkB‐DSP interaction in SNI mice at 6 weeks post‐injury, compared to sham‐operated controls (Figure 10H–N).

### 2.10 Combination of Na_v_1.7 and Na_v_1.8 Blockers Inhibits Action Potentials

**FIGURE 11:**
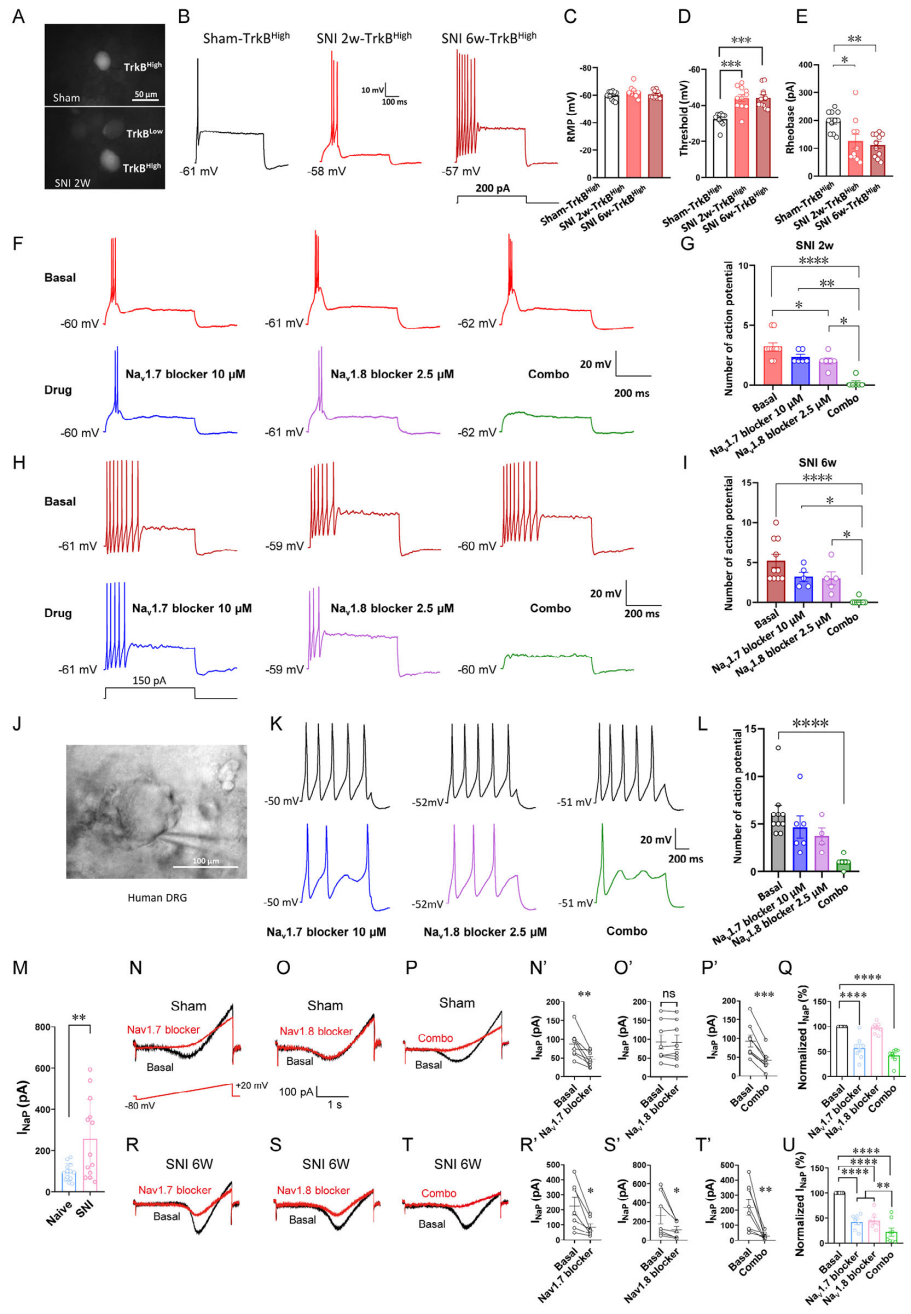
Combined inhibition of Na_v_1.7 and Na_v_1.8 blocks action potentials in hyperexcitable DRG neurons. (A) Representative images of neurons in DRG showing low or high expression of TrkB in *TrkB^2A‐Tomato/+^* mice subjected to SNI or not. Scale bar, 50 µm. (B) Representative firing diagrams for DRG neurons expressing high levels of TrkB in sham or SNI mice at 2 weeks and 6 weeks post‐injury. (C‐E) Resting membrane potential (C), threshold (D) and rheobase (E) of neurons like those shown in panel B (n = 10–11 neurons from 2–3 animals per condition). (F–I) Comparison of the Na_v_1.7 blocker PF‐05089771 (10 µm) and Na_v_1.8 blocker PF‐04885614 (2.5 µm), alone or together, for inhibiting the multiple firing of neurons like those in panel B. These experiments were performed at 2 weeks post‐injury (F,G) (*n* = 6–10 neurons from 2–3 animals per condition) or 6 weeks post‐injury (H‐I) (*n* = 5‐10 neurons from 2–3 animals per condition). (J) Representative micrograph of whole‐mount DRG from a patient with NP after BPA. Scale bar, 50 µm. (K) Typical firing diagrams of neurons in whole‐mount DRG as in panel J in the absence (*above*) or presence of the single or combined blockers at the indicated doses (*below*). (L) The combination of Na_v_1.7 blocker PF‐05089771 (10 µM) and Na_v_1.8 blocker PF‐04885614 (2.5 µm), but not either one alone, significantly inhibited the multiple firing of DRG neurons from the patient with NP after BPA. Data are shown for 4–10 neurons from two patients. (M–Q) Effects of sodium channel blockers on persistent sodium current (I_NaP_). I_NaP_ current was significantly increased in TrkB/Tomato‐expressing DRG neurons in SNI *TrkB^2A‐Tomato/+^* mice than in sham *TrkB^2A‐Tomato/+^* mice at 6 weeks post‐injury (M, *n* = 13, neurons from 2–3 animals per condition), and I_NaP_ current in Tomato‐expressing DRG neurons of sham mice was significantly blocked by Na_v_1.7 inhibitor PF‐05089771 (10 µM) (N, N’, Q), but not affected by Na_v_1.8 inhibitor PF‐04885614 (2.5 µm) (O, O’, Q), and combination of PF‐05089771 and PF‐04885614 did not inhibit I_NaP_ current more than PF‐05089771 (P, P’, Q). While I_NaP_ current in Tomato‐expressing DRG neurons of SNI mice at 6 weeks post‐injury was significantly blocked by 10 µm PF‐05089771 (R, R’, U), or 2.5 µm PF‐04885614 (S, S’, U), combination of PF‐05089771 and PF‐04885614 further reduced I_NaP_ currents (T, T’, U). n = 7‐15 neurons from 4–6 animals per condition. Data are shown in mean ± SEM. n.s.: not significant, * *p* < 0.05, ** *p* < 0.01, *** *p* < 0.001, **** *p* < 0.0001, by one‐way ANOVA test (C, E, and U) or Kruskal‐Wallis test (D, G, I, and L) or unpaired *t* test with Welch's correction (M), or paired *t* test (N’, O’, P’, R’, and T’) or Wilcoxon matched‐pairs signed rank test (S) or Kruskal‐Wallis test with Dunn's multiple comparisons test (Q).

Next, we directly examined whether Na_v_1.7/Na_v_1.8 SMACs modulate the firing properties of LTMR DRG neurons. Whole‐cell patch‐clamp recordings revealed that SNI significantly reduced the activation threshold of L4‐5 DRG neurons, and unexpectedly, induced a shift in their firing pattern from single spike to repetitive discharges in TrkB LTMR neurons. Notably, the frequency of these repetitive discharges continuously escalated over time (from 2 weeks to 6 weeks) following the injury (Figure 11A–E), suggesting a time‐dependent enhancement in neuronal excitability under NP conditions. At 2 weeks post‐injury, treatment with either Na_v_1.7 blocker PF‐05089771 or the Na_v_1.8 blocker PF‐04885614 alone significantly suppressed the action potentials of TrkB LTMR neurons, and the combination of both blockers was more effective at suppressing activity (Figure 11F,G). However, by 6 weeks post‐injury, the hyperexcitability became resistant to treatment with either blocker alone, but it was substantially inhibited by the combination of both blockers and exhibited frequency‐current relationships. This inhibition was reversible, as it could be washed out within 15–30 min (Figure 11H,I and Figure S25A–D). Consistent with our findings in the SNI model, analogous results were observed in whole‐cell patch‐clamp recordings of DRG neurons obtained from patients who had been suffering from severe NP for over 3 months due to BPA (Figure 11J–L). The overfiring and its blockade were associated with amplitude of total Na^+^ currents which can be significantly reduced by 71% by combination of blockers, but not by single blocker on its own (Figure S26A–C). These findings highlight the synergistic function of Na_v_1.7 and Na_v_1.8 within SMACs in exacerbation of NP. Since it is reported that Na_v_1.7 gain‐function mutation could increase isolated persistent sodium current (I_NaP_) of DRG neurons [53], which was proven to be Tetrodotoxin‐sensitive (TTX‐S) sodium current. It is reasonable to speculate that I_NaP_ current could increase in DRG neurons containing SMAC. Indeed, I_NaP_ current was increased 2.7‐fold in SMAC‐containing TrkB/Tomato^+^ DRG neurons from *TrkB^2A‐Tomato/+^* SNI mice than in non‐SMAC‐containing TrkB DRG neurons from *TrkB^2A‐Tomato/+^* sham mice at 6 weeks post‐injury (Figure 11M). Interestingly, while 54% of I_NaP_ current in non‐SMAC‐containing Tomato‐expressing DRG neurons of sham mice was blocked by Na_v_1.7 inhibitor PF‐05089771, but it's not affected by Na_v_1.8 inhibitor PF‐04885614, the combination of PF‐04885614 and PF‐05089771 didn't further reduce I_NaP_ current beyond 62% (Figure 11N–Q). In contrast, in SMAC‐containing neurons from SNI mice, the same concentration of either Na_v_1.7 or Na_v_1.8 inhibitor blocked 65% and 61% of I_NaP_ current, respectively. Remarkably, combining PF‐05089771 and PF‐04885614 reduced I_NaP_ currents by 91% (Figure 11R–U). These findings illustrate the pivotal role of SMACs in enabling Na_v_1.7 to generate higher I_NaP_ and enabling Na_v_1.8 to regulate I_NaP_ current through interacting with Na_v_1.7, highlighting the synergistic function of Na_v_1.7 and Na_v_1.8 within SMACs in exacerbation of NP.

### 2.11 SMAC Coordinates Repetitive Firing

**FIGURE 12:**
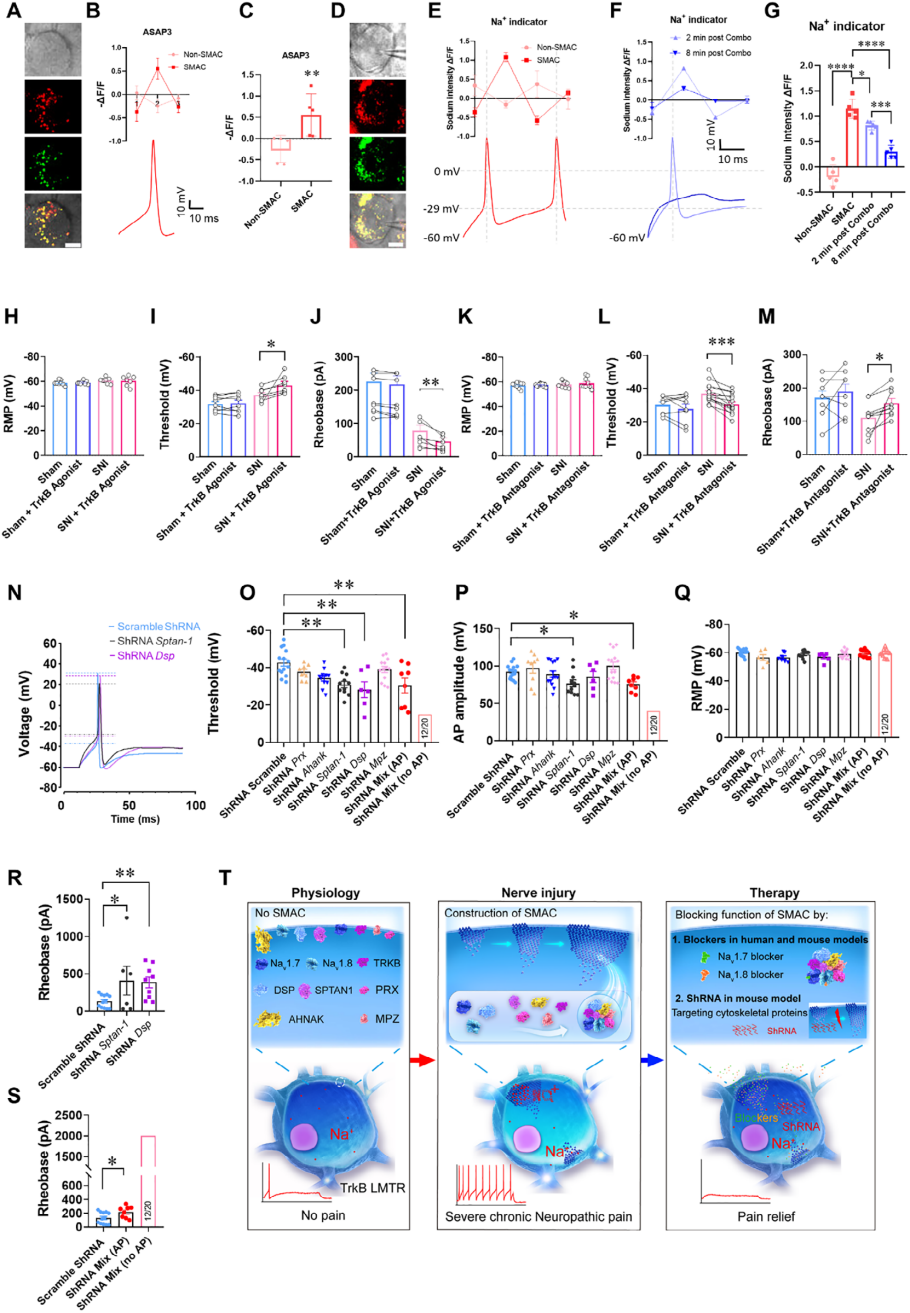
SMAC of Na_v_1.7/Na_v_1.8/TrkB coordinates repetitive firing. (A) Images of patch‐clamp recorded ASAP3‐expressing (green) Tomato (red) DRG neurons derived from *TrkB^2A‐Tomato/+^* SNI mice at 6 weeks post‐injury. (B) The change of ASAP3 intensity (potential, upper panel) within SMAC region during an action potential (lower panel) of injured TrkB DRG neurons. (C) The change of ASAP3 intensity within SMAC region and parallel non‐SMAC region. (D) Images of patch‐clamp recorded Tomato (red) DRG neurons which were derived from *TrkB^2A‐Tomato/+^* SNI mice at 6 weeks post‐injury and incubated in sodium indicator (green). (E) The change of sodium indicator intensity (green) within SMAC region and parallel non‐SMAC region (upper panel), and the change of potential recorded in stimulated TrkB neurons (lower panel). (F) Inhibition of the change of sodium indicator intensity within SMAC region and the generation of action potential of TrkB neurons 2 min (light blue) and 8 min (blue) after addition of combo of PF‐05089771 and PF‐04885614. (G) The change of sodium indicator intensity (green) within non‐SMAC region and parallel SMAC region, as well as SMAC region after incubation of combo. (H–M) TrkB regulated voltage threshold and rheobase of injured DRG neurons. TrkB agonist 7, 8‐Dihydroxyflavone (500 nm) didn't affect resting membrane potential (RMP) in Tomato‐expressing DRG neurons of sham as well as SNI mice at 6 weeks post‐injury (H), but reduced both action potential threshold (I) and rheobase (J) in DRG neurons from SNI, not sham, mice. Vice versa, TrkB antagonist ANA‐12 (100 µm) didn't affect RMP in Tomato‐expressing DRG neurons of Sham as well as SNI mice at 6 weeks post‐injury (K), but elevated both action potential threshold (L) and rheobase (M) from SNI, not sham, mice. *n* = 7–14 neurons from 6–7 animals per condition. (N–Q) Knockdown cytoskeletal proteins of SMAC affected electrophysiological properties of DRG neurons. Typical curves of action potentials generated in DRG neurons from SNI mice with injection of scrambled shRNA, shRNA *Sptan1*, or shRNA *Dsp* expressing virus (N). Knock‐down of SPTAN1 or DSP significantly elevated threshold of action potential, and knock‐down of five proteins significantly elevated threshold of action potential or blocked the generation of action potential (O). Knock‐down of SPTAN1 or five proteins together significantly reduced amplitude of action potential (P). Knock‐down of anyone or five proteins together didn't affect RMP (Q). Right bar in O‐Q showed 60% (12/20) of DRG neurons failed to generate action potential. (R, S) Knock‐down of SPTAN1 or DSP (R) or five cytoskeletal proteins of SMAC (S) can significantly elevate rheobase. *n* = 5–14 neurons from 2–3 animals per condition. * *p* < 0.05, ** *p* < 0.01, *** *p* < 0.001, **** *p* < 0.0001 by Mann Whitney test (C) or one‐way ANOVA test (G‐H, K and O‐Q) or paired *t* test (I‐J and L‐M) or Kruskal‐Wallis test (R) or unpaired *t* test (S). (T) Following nerve injury, cytoskeletal proteins SPTAN1, DSP, AHNAK, PRX, and MPZ facilitate the assembly of Na_v_1.7, Na_v_1.8, and TRKB into 3D SMACs. Blocking SMAC function, either by using a combination of Na_v_1.7 and Na_v_1.8 blockers or by shRNA‐targeting these cytoskeletal proteins, effectively inhibits action potentials in pathological DRG neurons, resulting in significant pain relief.

To further investigate the function of SMAC in NP, firstly we examined whether Na_v_1.7/Na_v_1.8 SMAC directly affects intracellular sodium ion flow. The change of potential in SMAC area of TrkB LTMR neurons was significantly bigger than non‐SMAC area during an action potential, as showed by the fluorescent voltage indicator, ASAP3 [54] (Figure 12A–C). The results from experiments of two‐photon laser‐scanning and patch‐clamp recording in parallel showed that Na_v_1.7/Na_v_1.8 SMAC accumulated sodium ions locally. Imaging with CoroNa Green (a low‐affinity Na^+^ indicator; Kd ≈ 50–80 mM, with relatively slow on/off kinetics) revealed that the Na^+^‐dependent fluorescence signal remained confined to SMAC‐associated regions for several minutes after stimulation (Figure S27A), creating a Na^+^ potential difference, and that sodium ions continuously flowed into SMAC area of TrkB neurons even after the peak of the first firing triggered by the stimuli, and then sodium ions effluxed out from the SMAC area once it was full charged with Na^+^. This outflow of sodium ions from SMACs facilitated the second firing of TrkB neuron in SNI mice at 6 weeks post‐injury (Figure 12D,E), because treatment of PF‐05089771 and PF‐04885614 for 2 min can significantly inhibit flow of Na^+^ into SMAC area and block the initiation of the second action potential (Figure 12F,G). Given Na_v_ channels on plasma membrane were blocked prior to those in SMAC area during diffusion of Na_v_ channels blockers from cell membrane to intracellular area, that inhibition of first action potential only happened until 8 min after addition of the combination of blockers (Figure 12F) suggests that flow of sodium ions into SMAC area also facilitated the generation of first action potential when TrkB neurons were stimulated by five electro pulses. These findings suggest that Na_v_1.7/Na_v_1.8 SMACs, alongside a high concentration of Na^+^, act like a bio‐transistor, significantly amplifying sodium currents to robustly promote repetitive firing in injured DRG neurons.

Secondly, we explored if TrkB in SMACs affects function of Na_v_ channels. Application of a TrkB agonist significantly reduced action potential threshold by 6 mV and decreased rheobase within 3–5 min, without affecting resting membrane potential (RMP), in SMAC‐containing Tomato‐expressing DRG neurons from SNI mice, not in non‐SMAC‐containing TrkB DRG neurons from sham mice at 6 weeks post‐injury (Figure 12H–J). Vice versa, administering a TrkB antagonist can elevate both action potential threshold and rheobase rapidly, without affecting RMP, in SMAC‐containing Tomato‐expressing DRG neurons from SNI mice, not in non‐SMAC‐containing TrkB DRG neurons from sham mice (Figure 12K–M). These data indicate that SMACs confer upon TrkB the ability to modulate the action potential threshold and rheobase, likely through interactions with Na_v_1.7 and Na_v_1.8. This modulation suggests a pivotal role for TrkB within SMACs in regulating neuronal excitability, offering insights into the complex interplay between these components in the pathophysiology of NP.

Thirdly, we measured the function of cytoskeletal proteins in SMACs. Patch‐clamp recording revealed that knock‐down of all five components (PRX, AHNAK, MPZ, SPTAN1, and DSP) of SMACs significantly elevated threshold of action potential and decreased amplitude of action potential in 40% of DRG neurons from SNI mice at 6 weeks post‐injury, without affecting their RMP when compared to that in scrambled shRNA‐expressing virus injected control SNI mice (Figure 12N–Q). Remarkably, knock‐down of all five components inhibited action potential generation entirely in 60% of DRG neurons, even with injection currents elevated up to 2000 pA (Figure 12O–Q and Figure S27B–G). The knockdown of SPTAN1 alone significantly increased the action potential threshold and reduced its amplitude, while knockdown of DSP alone significantly elevated the action potential threshold (Figure 12O) and reduced action potential amplitude (Figure 12P) without affecting the membrane potential (Figure 12Q). Additionally, knock‐down of all five components, or SPTAN1, or DSP, led to a substantial increase in rheobase, which is directly related to the function of voltage‐gated sodium channels (Figure 12R,S). These results, coupled with the observation that knock‐down of all five components blocked SMACs formation and mitigated severe NP, support that SMACs exacerbate NP through lowering rheobase and voltage threshold while increasing voltage amplitude, and that cytoskeletal proteins DSP and SPTAN1 are crucial for the formation and function of SMACs.

## 3 Discussion

Clustering of receptors in membranes can amplify the downstream signals induced by the same amount of ligand [25, 26], and changes in the size and geometric structure of SMACs containing receptors can boost signaling amplitude [26, 28]. Here we show that nerve injury promoted Na_v_1.7, Na_v_1.8, and TrkB to form polygonal lattice clusters and subsequently assembled into big SMACs in nerve and soma of both human and mouse DRG neurons (Figures 1, 2, 3 and 5). TrkB LTMR DRG neurons containing Na_v_1.7/Na_v_1.8 SMACs had lower action potential threshold and capability of repetitively firing (Figure 11), of which the underlying mechanism is that numerous lattice structured Na_v_1.7 and Na_v_1.8 within SMACs can be easily activated by subthreshold stimulus to open at same time, leading to accelerate and enlarge sodium flow into SMACs to generate multiple action potentials. Since it is known that activation of TrkB LTMR DRG neurons under injury conditions directly contributes to NP, the formation of Na_v_1.7/Na_v_1.8 SMACs in these neurons substantially exacerbates NP. Many Na^+^ ions were retained and enriched in SMACs (Figure S27A) as voltage‐gated sodium channels can bind sodium ions [55], leading to formation of an intracellular Na^+^ potential difference within SMAC region. So, Na^+^‐enriched SMACs function as a positive bio‐transistor to facilitate subthreshold stimuli to induce action potential and repetitive firing. Closely assembled Na_v_1.7 and Na_v_1.8 in highly condensed clusters within Na^+^‐fulfilled SMACs enable increase of total sodium currents (Figures 1, 2, 3, and 12). Combined inhibition of Na_v_1.7 and Na_v_1.8, neither alone, can block the action potential and mechanical allodynia, suggesting interaction and cooperation of Na_v_1.7 and Na_v_1.8 existing in SMACS.

Given that rheobase is the minimal electrical current injected into a cell to elicit an action potential and depends on Na_v_s and RMP [56], and that Na_v_1.7 is able to boost weak depolarization to initiate action potential firing [57, 58] and conformational change of Na_v_1.7 caused by gain‐of‐function mutations lowered voltage threshold by 8 mV [53, 59], it's reasonable to speculate that TrkB rapidly regulated voltage threshold and rheobase (Figure 6 and Figure 12H–M) through interacting with Na_v_1.7 and Na_v_1.8 within SMACs.

CoroNa Green is a lowaffinity indicator (Kd ≈ 50–80 mm) with relatively slow kinetics (time constants several to tens of ms), making it suboptimal for resolving sub‐millisecond Na^+^ transients. However, these properties were deliberately leveraged: low affinity minimizes saturation under high local Na^+^ loads, while the slower off rate enables the dye to act as an integrative tracer, facilitating visualization of net Na^+^ flux direction—an advantage for our focus on Na^+^ trafficking rather than instantaneous concentration. Na^+^ influx through densely clustered Na_v_1.7/Na_v_1.8 channels generates a transient elevation of submembrane Na^+^ selectively within SMAC regions, persisting beyond the first action‐potential peak due to restricted diffusion in the crowded lattice (intermolecular distances ∼30–70 nm). This local depolarizing drive, facilitated by possible electrostatic retardation from negatively charged residues [60] and reduced Na^+^/K^+^ ATPase capacity in injured neurons [61, 62], contributes to the initiation of repetitive firing in TrkB^+^ neurons of SNI mice at 6‐week post‐injury (Figure 12D–G).

We identified five cytoskeletal proteins (SPTAN1, DSP, AHNAK, PRX and MPZ) promote formation of SMAC of Na_v_1.7/Na_v_1.8. PLA demonstrated that TrkB directly binds to DSP, and DSP in turn interacts with SPTAN1, AHNAK and MPZ to form a scaffold, while SPTAN1 further recruits Na_v_1.7/Na_v_1.8, and Na_v_1.8 associates with PRX, facilitating SMAC assembly (Figures 9 and 10). Knockdown of SPTAN1 or DSP significantly blocked the formation of SMACs and increased the rheobase and voltage threshold, and that knockdown of five cytoskeletal proteins blocked generation of action potential in 60% of DRG neurons from SNI mice at 6 weeks post‐injury (Figure 12N–P). Interestingly, knockdown of SPTAN1 also reduced action potential amplitude (Figure 12P), this could be due to the fact that SPTAN1 organizes Na_v_1.8 into polygonal lattice structure because it's known that Na_v_1.8 participates in rising phase of action potential [7, 63, 64]. These findings prove that SPTAN1 and DSP are important for SMAC formation as well as Na_v_1.7 and Na_v_1.8 are required to be structured along cytoskeletal proteins SPTAN1 and DSP to initiate action potential.

Previous studies have revealed that TrkB‐expressing LTMR neurons (often large‐diameter) and nociceptors (small‐diameter) both contribute to pain sensitization and mechanical allodynia in neuropathic states [17, 20, 38, 39, 65, 66, 67]. Our findings that SMACs develop in both TrkB^+^ (predominantly large) and TrkB^−^ DRG neurons across a wide size range (small to large in humans; including small nociceptors in mice) suggest that these supramolecular clusters represent a convergent pathological mechanism enabling contributions from both A‐fiber mechanoreceptors and C‐fiber nociceptors to chronic NP. Although the Na_v_1.7 antibody used in this study was not validated using knockout tissue, the Na_v_1.8 antibody (NeuroMab clone N134/12, catalog #75–166) was validated by the supplier using Na_v_1.8 knockout mouse DRG neurons. In addition, we performed overexpression validation for both the Na_v_1.7 and the Na_v_1.8 antibodies in HEK293 cells (Figure S22A), which demonstrated clean, isoform‐specific signals without cross‐detection of Na_v_1.7, Na_v_1.8, or Na_v_1.9; and the specificity of the Na_v_1.7 antibody was also confirmed using *Scn9a^HA/+^* knock‐in mice, where an HA antibody specifically detects the tagged Na_v_1.7 protein (Figure S3D,E). Our findings align with the reported expression patterns of Na_v_1.7 in DRG neurons and extend these observations across species. While Black et al. (2012) described Na_v_1.7 as “robustly expressed in small‐diameter DRG neurons” with limited intensity in rare large neurons [68], modern single‐cell sequencing data from mice, macaques, and humans consistently demonstrate *SCN9A* expression in *NF* ^+^ large neurons, including TrkB LTMRs [35, 36, 37, 69]. Moreover, our HA immunostaining data reveal that Na_v_1.7 is widely expressed across all neuron sizes, with varying expression levels within each size category. Notably, some medium to large DRG neurons exhibit highest total Na_v_1.7 protein level (integrated intensity), although certain small‐sized DRG neurons show the highest Na_v_1.7 intensity (Figure S5G,H). Over 73% TRKB SMAC DRG neurons from BPA patients have diameters > 60 µm. The consistency across species at both mRNA and protein levels underscores the biological significance of Na_v_1.7 in both nociceptive and non‐nociceptive pathways. Our findings also align with the reported expression patterns of Na_v_1.8 and electrophysiologic data in DRG neurons in cross‐species. While Na_v_1.8 expression or Na_v_1.8‐like TTX‐resistant sodium currents were often reported more restricted expression in rodents, predominantly in small‐ to medium‐diameter nociceptors (∼50–70% nociceptors) [8, 70, 71], data from Na_v_1.8‐Cre lines demonstrate that 75% of DRG neurons express Na_v_1.8‐Cre, including >90% of neurons expressing markers of nociceptors and, unexpectedly, a large population (∼40%) of neurons with myelinated A fibers [71]. This is consistent with our immunostaining data (70%–80% of DRG neurons positive), single‐cell sequencing data showing *Scn10a* expression in 4 out of 5 major DRG neuron populations in mice (https://www.linnarssonlab.org/drg/) [35], Ramachandra et al.’s immunostaining results (48% of large neurons positively stained in adult mice) [72], and electrophysiological evidence of Na_v_1.8‐like TTX‐resistant sodium current in large cutaneous afferent DRG neurons [73]. Similarly, in adult human DRG, 60–80% of large diameter neurons (60–80 µm) were positively labeled with an Na_v_1.8 antibody [72, 74], consistent with single‐cell sequencing data (∼80% of human DRG neurons that express GAPDH also express *SCN10A*, https://ernforsluolabs.shinyapps.io/HumanDRG/) [37] and electrophysiological evidence that Na_v_1.8‐like currents show broad distribution across neuron diameters, including medium‐ and large‐diameter neurons [75]. Moreover, Recent human DRG recordings further support broad Na_v_1.8 channel presence and function, with nearly all tested neurons (191 out of 195, 98%) showing clear Na_v_1.8‐like currents or sensitivity to selective inhibitors) [76]. Following nerve injury, the upregulation of Na_v_1.7 and Na_v_1.8 likely arises from dual mechanisms: transcriptional increases in *Scn9a*/*Scn10a* mRNA and post‐transcriptional de‐repression mediated by injury‐induced downregulation of miRNAs such as *miR‐96* [34]. The dynamic change in the number of Na_v_1.7/Na_v_1.8 co‐expressing neurons along injury time‐course mirrors oscillatory *Scn9a* expression patterns observed in NF1/NF2 neuronal subtypes along sciatic nerve transection time‐course [69]. Notably, species‐specific differences in expression pattern—such as higher Na_v_1.7 and lower Na_v_1.8 expression in human DRG versus the inverse in mice [77]—should be considered when extrapolating preclinical findings to clinical applications.

Due to the well‐recognized ethical and logistical challenges in obtaining truly healthy adult human DRG tissue (i.e., from individuals without pre‐mortem pain or neurological conditions), we used DRG from a 29‐week human fetus as our “normal” control. The developmental stage of a 29‐week human fetus corresponds most closely to the late gestation to early postnatal period in mice, particularly around E18.5 to P7–P10 [78, 79]. In rodents, Na_v_1.8 mRNA and protein expression reaches adult‐like levels by postnatal day 7 [80]. Critically, the SMACs of Na_v_1.7/Na_v_1.8 we describe were observed exclusively in DRG from adult mouse models of NP and were entirely absent in healthy adult mouse DRG (both in our own staining and in independent reports). This injury‐specific, cross‐species pattern strongly supports that SMAC formation represents a pathological hallmark in NP rather than a developmental feature. We plan to replicate these key findings using adult post‐mortem DRG from healthy donors when such ethically approved samples become available in future extensions of this work.

Na_v_1.7 blocker PF‐05089771, Na_v_1.8 blockers PF‐04885614 and PF‐04531083 developed by Pfizer exhibited high selectivity. Alexandrou et al. [81] reported PF‐05089771 with IC_50_ = 8, 11 nm for mouse and human Na_v_1.7, respectively; its IC_50_ values are 0.11, 0.16, 0.85, 10, 11, 25, and >10 µm for Na_v_1.2, Na_v_1.6, Na_v_1.1, Na_v_1.4, Na_v_1.3, Na_v_1.5, and Na_v_1.8, respectively. PF‐04885614 was reported with IC_50_ = 53 nm for human Na_v_1.8 channel [43], showing IC_50_ values are 4.2, 7.0, 11, 16 and 27 µm for hNa_v_1.6, hNa_v_1.7, hNa_v_1.1, hNa_v_1.2, and hNa_v_1.5, respectively (https://www.tocris.com/products/pf‐04885614_4916). Information about Na_v_1.8 blocker PF‐04531083 remains undisclosed, though it advanced to clinical trials [82]. PF‐04885614 or PF‐04531083 demonstrated synergistic efficacy when combined with Na_v_1.7 blockers, indicating their Na_v_1.8‐specific inhibition and functional coordination between Na_v_1.8 and Na_v_1.7 in SMAC. However, only the Na_v_1.7 blocker PF‐05089771 (phase II trial completed), not GNE‐0439, showed synergistic analgesic efficacy when combined with Na_v_1.8 blockers. Detailed chemical property information for GNE‐0439—which likely did not meet the criteria for preclinical candidate selection—remains limited, including its selectivity relative to Na_v_1.8, potential non‐specific binding to other targets (ion channels, receptors), pharmacokinetic properties, and effects on absorption, transporters, and metabolic enzymes. Whether it has potential drug–drug interactions with the Na_v_1.8 blocker PF‐04885614 is also a critical consideration, particularly in the context of densely packed Na_v_1.8 and Na_v_1.7 within SMAC clusters, where both channels bind to the scaffold protein SPTAN1. In this setting, conformational changes induced by GNE‐0439 could potentially affect the structure of Na_v_1.8. Therefore, the lack of synergistic analgesic effect may be attributed to multiple factors, including the use of Na_v_1.7‐selective chemical scaffolds and non‐pore‐blocking mechanisms by GNE‐0439 [46], as well as critical differences in state dependence and allosteric coupling to Na_v_1.8—especially when Na_v_1.7/Na_v_1.8 SMAC develop in DRG neurons from NP animal models—and differences in pharmacokinetic alignment. Together, these aspects may collectively contribute to the failure to inhibit SMAC function and chronic NP.

Although our study primarily focused on neuronal mechanisms underlying SMAC formation, we cannot exclude potential contributions from non‐neuronal cells or inflammation in the injured DRG. Satellite glial cells (SGCs), Schwann cells, and myelin components undergo significant phenotypic and functional changes following peripheral nerve injury, including upregulation of glial fibrillary acidic protein (GFAP), altered potassium buffering, enhanced gap junction coupling, and release of pro‐inflammatory cytokines such as IL‐1β and TNF‐α [83]. These glial alterations can modulate neuronal excitability and membrane protein organization. Similarly, inflammatory responses involving immune cell infiltration and cytokine signaling may indirectly influence ion channel clustering through changes in the local biochemical and mechanical microenvironment [25]. Schwann cells, in particular, play critical roles in nodal architecture and sodium channel clustering at nodes of Ranvier via direct axo‐glial interactions [47, 48, 84, 85]. Notably, satellite glial cells in sensory ganglia have emerged as important contributors to chronic pain [86, 87], with accumulating evidence supporting their activation and neuro‐glial interactions in the development and maintenance of NP states. Future studies will be important to dissect the relative contributions of neuronal‐intrinsic cytoskeletal interactions versus glial‐ or inflammation‐driven mechanisms to the formation and maintenance of pathologic SMACs.

## 4 Conclusion

Our study demonstrated that formation of Na_v_1.7/Na_v_1.8/TrkB SMACs was facilitated by TrkB/CREB signaling pathway and the cytoskeletal proteins (SPTAN1, DSP, AHNAK, MPZ, and PRX) in injured‐DRG neurons in both human and mouse during the progress of NP. TrkB directly binds to DSP, and DSP in turn interacts with SPTAN1, AHNAK, and MPZ to form a scaffold, while SPTAN1 further recruits Na_v_1.7/Na_v_1.8, and Na_v_1.8 associates with PRX, facilitating SMAC assembly. The SMAC, packing a total of approximately 785 000 Na_v_1.7/Na_v_1.8 molecules with density of ∼1242 Na_v_1.7/Na_v_1.8 molecules/µm^3^ (Table S1) and enriched with Na^+^, forms a potential difference and act as a bio‐transistor to facilitate the generation of action potential, contributing substantially to hyperexcitability of LTMR DRG neurons and the exacerbation of severe NP. Inhibition of Na_v_1.7 and Na_v_1.8 can block the action potential of both human and mouse injured DRG neurons, as well as alleviate NP in mouse models with high efficacy, while knockdown of cytoskeletal proteins within SMACs can effectively eliminate NP through blocking the formation of SMACs in mouse models (Figures 8 and 12T). These findings position the Na_v_1.7/Na_v_1.8 SMAC as a promising therapeutic target for NP, indicating that a combined blockade of Na_v_1.7 and Na_v_1.8 is required to achieve efficient relief of chronic NP.

## 5 Experimental Section/Methods

### 5.1 Sex as a Biological Variable

Transgenic mice (*Scn9a^HA^* tag mice and *TrkB^2A‐Tomato/+^*) were used in both sexes for SMAC immunostaining and electrophysiology in DRG neurons, and human DRG samples were from both male and female patients. No sex differences were observed in Na_v_1.7/Na_v_1.8 SMAC formation or channel properties. Pharmacological studies used only adult male mice to minimize animal numbers, as no sex differences appeared in core mechanistic assays. This is consistent with limited/no major sex dimorphism in DRG Na_v_ channel expression and neuropathic pathology in rodents and humans [77, 88].

### 5.2 Human Subjects

Human pathological DRGs were collected from 7 patients with brachial plexus avulsion (BPA), the avulsed roots of the affected brachial plexus and the dislocated cervical DRG tissues were resected during the surgical process of nerve reconstruction or transplantation under the ethic permit (KY20222228) proved by the Ethics Committee of Xijing Hospital, the first affiliated hospital of Fourth Military Medical University. All BPA patients included in the study suffered from severe NP in the affected extremities, with the VAS scale higher than 6. Normal cervical DRG tissues from 2 naturally aborted 29‐week human embryos were collected according to the ethic permit (MECDU‐201909‐1) proved by the Medical Ethics Committee of Dali University. Patients involved in this study have signed the informed consent form, and all specimens were handled in an anonymized way according to ethical and legal standards. The collected human tissues were subjected to biological analysis and patch clamp recording.

### 5.3 Animals

In this study, all animals were housed with a maximum of 6 per cage, at 21°C, 50% humidity, on a 12 h light: 12 h dark schedule in the SPF standard animal facility, and all animal work was conducted under ethical permission (TJLAC‐017‐040, TJAA08621402, TJAA08625101, and IACUC‐20230211) from Tongji University and Fourth Military Medical University ethical review panel.

*Scn9a^HA^* tag mouse was generated by knocking a hemagglutinin (HA) coding sequence into upstream of the stop codon of *Scn9a* transcript (NM_001290675.1, 1973 residues) by Genome Tagging Project (GTP) Center, SIBCB, CAS, and was genotyped by PCR using primer pairs: (*Scn9a‐HA*‐F: 5′ TCACAACCACACTGAAGCGA 3′, *Scn9a‐HA*‐R: 5′ ATCCGGCACATCATACGGAT 3′, *Scn9a*‐ WT‐F: 5′ GCAGGAAATAGAGCTTCGGT 3′, *Scn9a*‐ WT‐R: 5′ TCACAGCTAATCCTGTTTGTGTT 3′).

*TrkB^2A‐Tomato/+^* mice were generated through knocking 2A‐Tomato cassette into upstream of the stop codon of exon 18 of *TrkB* which is only presented in the full length *TrkB* transcript by the CRO Company (Shanghai Model Organisms Center, Inc.) using CRISPR/Cas9 technology. Briefly, in vitro transcribed *Cas9* mRNA and gRNA with homologous arms‐encompassed targeting vector were injected into fertilized eggs of C57BL/6J mice, and positive F0 mice genotyped by PCR with specific primers (PI: 5′ TTTTTCTTCCCGGATTTTCTCGT 3′, PII 5′ TCTCATGGCCTTTAGGTGGTCAGT 3′, PIII 5′ TCCTTGCGCCGGTGATGGTT 3′, PIV 5′ CTTCCCGGCTGTCTTGTTTGTATG 3′) were crossed with *wild type* C57BL/6J mice to get F1 heterozygous mice which were identified by genotyping PCR with primer pairs (P1: 5′ CACACCCGGAAGAACATCAAGA 3′, P2 5′ CGTCAAAGGCAGGAATACAACAAG 3′, P3 5′ GTTGGTAAATGGTTAAGGAGACACACG 3′, P4 5′ GGACTCCACGTCTCCCGCCAACT 3′). Positive F1 *TrkB^2A‐Tomato/+^* mice were further analyzed using Southern‐blot to select only one‐site knock‐in clones.

#### 5.3.1 Animal Pain Models

Spared nerve injury (SNI) surgical procedures were performed under isoflurane‐induced anaesthesia as previously described [34]. Briefly, a small incision was made in the proximal one‐third of the lateral thigh on the left side of the mouse. The sciatic nerve was exposed through blunt dissection, and the tibial and common peroneal nerves were ligated with a surgical suture. A 1–2 mm segment of these nerves was then excised with micro‐scissors, while the sural nerve was left intact. For the sham surgery, the sciatic nerve was exposed but not manipulated further, and it was carefully returned to its original position. The skin wound was then sutured, and the mice were placed on a heating pad to recover.

Mouse model of diabetes: Wild type C57BL/6 mice at age of 4–5 weeks were intraperitoneally injected 55 mg/kg Streptozotocin (STZ, dissolved in 100 mmol/L citrate buffer, pH 4.5) after 12 h food‐fasting and deprived water for 2 h, followed by 4 consecutive intraperitoneal injection of 55 mg/kg STZ after 6 h food‐fasting once a day, 2 h water deprivation after each injection. Two weeks post final injection of STZ, blood glucose was measured after 6 h food‐fasting. Mice with fasting blood glucose more than 13 mM were designated to diabetic group. Mechanical threshold of diabetic mice was measured at 4 weeks and 10 weeks post final injection of STZ. Only the diabetic mice which have mechanical threshold≤0.4 g at 4 weeks were used for the later experiment. The animal number of initially assigned to the vehicle, DNP4w, and DNP10w groups was 12. However, one cage of DNP 4w mice (*n* = 6) was died due to the bad health condition before drug tests.

#### 5.3.2 Drug Treatments

For mouse SNI model, Na_v_1.7 channel blocker PF‐05089771 (Tocris Bioscience, 2, 4, and 8 mg/kg) and GNE‐0439 (ProbeChem, # PC‐62325, 20 and 40 µg/kg), or Na_v_1.8 channel blockers including PF‐04885614 (Tocris Bioscience, 45, 90, and 180 µg/kg) and PF‐04531083 (Tocris Bioscience, 10 and 20 mg/kg), or Gabapentin (Sigma, PHR1049‐1G, 50 mg/kg), or TrkB agonist (Sigma, D5446‐50MG, 7, 8‐Dihydroxyflavone, 5 mg/kg), or TrkB antagonist (Sigma, SML0209‐25MG, ANA‐12, 0.5 mg/kg) were administered individually via i.p. into adult male mice and mechanical threshold was tested 60 min after drug administration and finished within 90 min. TrkB agonist and antagonist were administered individually via i.p. once a day for 7 consecutive days from day 35 to day 42 post‐injury. The combination comprising three different doses of PF‐05089771 and PF‐04885614 (1 mg/kg: 45 µg/kg, or 2 mg/kg: 90 µg/kg, or 4 mg/kg: 180 µg/kg), or combined PF‐05089771 (2 mg/kg) and PF‐04531083 (10 mg/kg), or combined GNE‐0439 (20 µg/kg) and PF‐04885614 (90 µg/kg) were administered simultaneously via i.p. into adult male mice and mechanical threshold was tested 60 min after drug administration and finished within 90 min.

For mouse DNP model, Na_v_1.7 channel blocker PF‐05089771 (0.1, 0.2, and 0.3 mg/kg), or Na_v_1.8 channel blocker PF‐04885614 (10, 20, and 30 µg/kg) were administered individually via i.p. into adult male mice and mechanical threshold was tested 60 min after drug administration and finished within 90 min. The combination of PF‐05089771 (0.2 mg/kg) and PF‐04885614 (20 µg/kg) was administered simultaneously via i.p. into adult male mice and mechanical threshold was tested 60 min after drug administration and finished within 90 min.

#### 5.3.3 Behavioral Test

Before behavioral test, mice were placed in transparent plexiglass chambers on top of a raised wire mesh and allowed to acclimate for 20 min.

Mechanical threshold: The paw withdrawal threshold of the hind paws was measured with a set of calibrated monofilaments (von Frey hairs, DanMic Global, USA) in order of increasing forces from 0.008 to 2 g. Each monofilament was applied five times. The force at which the animal withdrew the paw from at least three out of five consecutive stimuli was recorded as paw withdrawal threshold. The little touch part of the side foot of the mice undergoing SNI was gently brushed with a soft‐bristled brush, and the mice were scored based on their responses.

Pinprick test: A 27‐gauge needle (the tip was cut to make the needle with a flat mouth) was gently applied to the plantar surface of the hind paw without penetrating the skin. A score system was used according to the extent of the response. 0‐ no response; 1‐ move, look around to see what happened; 2‐ brief quick lift or withdrawal or removal of hind paw; 3‐ brief quick shakes of hind paw, or jumps; 4‐ based on score 3, 1–2 time(s) extra high frequency shakes of hind paw.

#### 5.3.4 Synergy Assessment by Bliss Independence Model

Analgesic effects were evaluated using the von Frey test to measure mechanical paw withdrawal threshold. The analgesic effect percentage at 1 h after drug administration was calculated as:

$$\begin{array}{ccc} & & \mathrm{Analgestic}\;\mathrm{Effect}\left(\%\right)\\ & & \;=\frac{Posttreatment\;threshold-Baseline\;threshold}{Cutoff\;threshold-Baseline\;threshold}\times 100\% \end{array}$$

where baseline threshold was the pre‐drug mechanical pain threshold, post‐treatment threshold was the measured threshold at 1 h after drug administration, and cut‐off threshold was the maximum preset stimulus intensity 2 g.

For drug combination studies, analgesic synergy was determined using the Bliss independence model as previously described [44]. The expected additive analgesic effect of the combination was calculated as:

$$\;E_{additive}=E_{A\;}+E_{B}-E_{A}\times \;E_{B}$$

where E_A_ and E_B_ represent the analgesic effect (decimal fraction) of drug A and drug B alone, respectively. The observed analgesic effect (E_observation_) of the combination group was directly obtained from experimental measurements. Synergy was defined as:

$$Bliss\;ratio=E_{Observation}÷\;E_{additive}$$

indicating a greater‐than‐additive analgesic response. The analgesic effect percentage was plotted including the observed combination effect and Bliss expected effect for visual comparison.

### 5.4 Real‐Time Quantitative RT‐PCR

As previously described [34], total RNA was isolated from mouse L4‐6 DRG using Trizol Reagent (Thermo Fisher Scientific, Massachusetts, USA) and then reverse transcribed using the Revert Aid First strand cDNA synthesis Kit (Thermo Fisher Scientific, Massachusetts, USA, K1621) into cDNA. The expression level of *Scn9a* and *Scn10a* was quantified by real‐time PCR using SYB‐Green and ABI QuantStudio 3 machine (Thermo Fisher Scientific, Massachusetts, USA) with specific primers reported by Sun et al. [34], and then normalized to 18S ribosomal RNA.

### 5.5 Western Blot

Proteins were isolated from freshly collected L4‐6 DRGs from naive, diabetic, and SNI mice at 2 weeks or 6 weeks post operation, or from human cervical DRGs (3 DRGs from 3 BPA patients, including 20, 30, 57 years old males, and 3 DRGs from 2 naturally aborted embryos). The probing procedure was same as that previously described [89] except for primary antibodies against Na_v_1.7 (Abcam, ab85015, 1:800) and Na_v_1.8 (NeuroMab, 75–166, 1:1000), TrkB (R&D system, AF1494, 1:1000), GAPDH (Proteintech Group, 60004‐1‐Ig, 1:3000) and β‐ACTIN (Sigma‐Aldrich, A5441, 1:5000). The Chemiluminescence signals based on enhanced chemiluminescence (ECL) were detected by Gel Imaging System (Clinx, Shanghai, China; GenoSens2000). The exposure durations for Na_v_1.7, Na_v_1.8, TrkB, and GAPDH were 70 sec, 120 sec, 20 sec and 10 sec, respectively. The images were analyzed using Image J software (NIH, USA).

### 5.6 Immunostainings

Mouse DRGs were collected after transcardiac perfusion with 20 mL of ice‐cold saline followed by 20 mL of 4% paraformaldehyde (PFA) under anesthesia. Mouse and human DRGs were post‐fixed in 4% paraformaldehyde (PFA) for 24 and 48 h, respectively, and then in 30% sucrose for 24 h at 4°C, and embedded in OCT, further sectioned in thickness of 14 and 12 µm using a cryostat, respectively. Primary DRG cells were shortly cultured 3 h and then fixed in 4% PFA for 10 min, washed 3 times with 1x PBS for 5 min each time. Immunostainings were performed as previously described [39, 90]. DRG sections were washed in PBS for 5 min × 2 times, blocked in 10% bovine serum albumin (Sigma‐Aldrich, USA) in PBS containing 0.1% Triton X‐100 (Sigma‐Aldrich, USA) and incubated with primary antibodies at 4°C overnight. The following primary antibodies were used: mouse antibodies against Na_v_1.8 (NeuroMab, 75–166, 1:200), SPTAN1 (Spectrin α II, Santa Cruz, sc‐46696, 1:250), AHNAK (Santa Cruz, sc‐390743, 1:250), Flotillin2 (Flot2, Santa Cruz, sc‐28320, 1:200), PRX (Santa Cruz, sc‐137222, 1:500), DSP (Desmoplakin, Abcam, ab164341:100) and NeuN (Merck, MAB377, 1:200) and HA (Cell Signaling Technology, #2367S, 1:500), rabbit antibodies against Na_v_1.7 (Proteintech Group, 20257‐1‐AP, 1:200), SCN11A (Abcam, ab65160, 1:50), TrkB‐ATTO Fluor‐488 (Alomone labs, ANT‐019‐AG, 1:50), DsRed (Clontech, #632496, 1:500) and NeuN (Cell Signaling Technology, 24307S, 1:200), chicken antibody against MPZ (Myelin Protein zero, Abcam, ab39375, 1:500) and NF200 (Abcam, ab4680, 1:1000), and goat antibody against TrkB (R&D system, AF1494, 1:400). Secondary antibodies were conjugated with Alexa Fluor 488/555/647 (Thermo Fisher Scientific, Massachusetts, USA, 1:1000), and goat anti chicken Alexa Fluor 647 (ab150171, Abcam, USA), goat anti‐rabbit IgG conjugated with Atto 488 (Sigma, 18772, 1:500), and goat anti‐mouse IgG conjugated with Cy3B (CY‐HXFA395, Standard Imaging (Beijing) Biotechnology Co., Ltd, China, 1:50). Omitting primary antibody served as a negative control. Fluorescent images from sham and SNI groups were captured using the same parameter settings with Olympus confocal microscope (Olympus FV3000, Ishikawa, Japan) or Zeiss confocal microscope (LSM880, LSM780, LSM710, Carl Zeiss, Oberkochen, Germany) or fluorescence microscope (Vert.A1, Carl Zeiss, Oberkochen, Germany), and processed with Adobe Photoshop software. The microscopy parameters used for image acquisition and analysis were as follows: the image size: 1024 × 1024 pixels, the exposure time per pixel was 4.1 µs, scan mode: frame and average of 2. To get super‐resolution images, DRG neurons derived from SNI mice at 6 weeks post‐injury were cultured for 3 h, and then fixed and immunostained with antibodies against Na_v_1.7, Na_v_1.8 and TrkB. Then the images were captured using super resolution microscope (Eryla7, Carl Zeiss, Oberkochen, Germany or HIS‐SIM, Guanzhou CSR Biotech Co. Ltd, China), or Nikon N‐STORM microscope (Nikon, Japan). The procedure for Hessian imaging was performed by following the previous report [91], sparse deconvolution was used to further improve the resolution and contrast in reconstructed images according to the previous report [92]. In this test, 4 DRGs from 4 BPA patients, including 20, 30, 46, 57‐year old males, were included, and at least 4 sections of DRG tissues from different patients were used in the final quantitative analysis.

To determine position of SMAC, we recorded the entire procedure from dissection to embedding. DRGs were dissected with longer central axons attached, and whole DRGs were embedded in a consistent orientation to preserve in vivo polarity, ensuring sagittal sections were parallel to the DRG long axis. Immunostained sections were imaged at low magnification to visualize overall DRG architecture, soma distribution, and the emergence of central axons toward the spinal cord, and at high magnification to resolve SMAC localization relative to individual neuronal processes.

### 5.7 TUNEL Staining

Mouse DRG sections from sham and SNI mice at 2 and 4 weeks post‐injury were stained using in situ cell death detection kit (REF 11684795910, Roche, Germany) according to the manufacturer's manual, and then counterstained with DAPI. Images were captured using Zeiss confocal microscope LSM880.

### 5.8 Dil Staining

L5 DRG sections were immunostained for Na_v_1.7 and Na_v_1.8, at the end, the sections were incubated with 1 µm Dil and DAPI for 10 min. For live labelling, 1 µL Dil (10 mg/mL in DMSO, MCE, HY‐D0083, USA) was injected via a Microsyringe into the L5 DRG at multiple site. One hour after the injections, mice were sacrificed via anesthetic overdose and perfused with 30 mL of chilled 1 × PBS followed by 30 mL of 4% PFA for fixation. The corresponding DRG was postfixed in PFA for 2 h and then dehydrated using gradient sucrose solutions of 20% and 30%. The L5 DRGs were sectioned using cryostat in thickness of 14 µm, and the sections were further processed for immunostaining as described above.

### 5.9 Quantification Methods

In every sixth serial section from L4/L5 DRGs of sham and SNI mice at 2 or 6 weeks post‐injury, fluorescent images of Na_v_1.7, Na_v_1.8, TrkB, and NeuN were thresholded based on corresponding negative controls, and positive cells were counted using ImageJ software (NIH, USA) [93]. Three sections were analyzed per animal. Data were represented as mean ± standard error of the mean (mean ± SEM) with statistical analyses performed using GraphPad Prism 9 and specific tests detailed per experiment. The diameters of Na_v_1.7, Na_v_1.8 and TRKB and the intensity and integrated intensity of Na_v_1.7‐HA staining and the area of Na_v_1.7‐HA‐expressing neurons were measured in Image J. The number of TUNEL positive cells was counted on 4 sections from every ninth serial sections (the images were captured using Zeiss LSM880), and the number of cells with cluster of Na_v_1.7/Na_v_1.8 in sham and SNI mice at 2 and 6 weeks post‐injury (Figure 1B–D) was counted on 3 every sixth serial sections (the images captured using Carl Zeiss fluorescence microscope Vert.A1), the number of cells with cluster of Na_v_1.7/Na_v_1.8/TrkB, the area of SMAC and the density of clusters from Vehicle, TrkB agonist or antagonist treated SNI mice was counted or measured 3 every sixth serial sections (the images were captured using Zeiss LSM880), the number of cells with cluster of Na_v_1.7/ TrkB, the area of SMAC and the density of clusters (Figure 4I–K) from shRNA‐expressing virus treated SNI mice was counted or measured 2 every eighth serial sections (the images were captured using Zeiss LSM710) using ImageJ [94]. The number of positive HEK293 cells or cluster‐containing HEK293 cells was counted from 3 images of each independent experiment, which were randomly photographed using LSM780 with 20x objective.

The numbers of Na_v_1.7 and Na_v_1.8 in the SMAC were counted in five regions along the long axis within two column clusters that exhibited a gradient in density. The area volume was measured and calculated based on the image scale bars. The densities of Na_v_1.7 and Na_v_1.8 were calculated by dividing the number of molecules by the corresponding area volume.

The cell volume was estimated under the assumption that neurons are spherical, using the formula:

$$V=\frac{4A^{3/2}}{3\sqrt{\pi }}$$

where A is the cross‑sectional area of DRG neurons measured using ImageJ.

SMAC volume was determined from 100× magnification Z‑stack immunofluorescence images. The cross sectional area was manually outlined, and the longitudinal depth of each structure was measured along the Z‑axis. Volume was subsequently calculated using the acquired area and depth measurements.

Finally, the numbers of Na_v_1.7 and Na_v_1.8 molecules per SMAC were estimated by multiplying the mean density by the SMAC volume.

### 5.10 Immunoprecipitation and Mass Spectrometry

#### 5.10.1 Immunoprecipitation

L4‐6 DRG from SNI and Sham mice were lyzed using cell membrane protein and cytoplasmic protein extraction kit (Beyotime, China, P0033), and then the lysates were used for immunoprecipitation using the antibody against Na_v_1.7 (Proteintech Group, 20257‐1‐AP, 0.5 µg/µL), the antibody against Na_v_1.8 (NeuroMab, 75–166, 0.5 µg/µL) and IgG (Mouse, Santa Cruz, sc‐2025, 0.5 µg/µL) according to the manual of Dynabeads Protein A Immunoprecipitation Kit (Invitrogen, 10006D). The proteins pulled down were separated in 6%–10% gradient SDS‐PAGE gels and stained with silver staining kit (Beyotime, China, P0017S), and the bands which showed difference between Sham and SNI groups were collected and sent to Shanghai Applied Protein Technology co. Ltd for LC‐MS measurement data analysis.

#### 5.10.2 LC‐MS

The in‐gel proteins were reduced, alkylated and then digested overnight with 12.5 ng/µL trypsin in 25 mm NH_4_HCO_3_ solution. The peptides were extracted three times with 60% ACN/0.1% TFA. The extracts were pooled and dried completely by a vacuum centrifuge. Each fraction was injected for nanoLC‐MS/MS analysis. The peptide mixture was loaded onto a reverse phase trap column (Thermo Scientific Acclaim PepMap100, 100 µm × 2 cm, nanoViper C18) connected to the C18‐reversed phase analytical column (Thermo Scientific Easy Column, 10 cm long, 75 µm inner diameter, 3 µm resin) in buffer A (0.1% Formic acid) and separated with a linear gradient of buffer B (84% acetonitrile and 0.1% Formic acid) at a flow rate of 300 nL/min controlled by IntelliFlow technology. LC‐MS analysis was performed on a Q Exactive mass spectrometer (Thermo Scientific) that was coupled to Easy nLC (Proxeon Biosystems, now Thermo Fisher Scientific) for 30 min. The instrument was run with peptide recognition mode enabled. The MS data were analyzed using MaxQuant software version 1.5.3.17 (Max Planck Institute of Biochemistry in Martinsried, Germany) [95]. MS data were searched against a non‐redundant International Protein Index arabidopsis sequence database v3.85 from the European Bioinformatics Institute (https://www.ebi.ac.uk/). The cutoff of global false discovery rate (FDR) for peptide and protein identification was set to 0.01. The mass spectrometry proteomics data have been deposited to the ProteomeXchange Consortium via the PRIDE [96] partner repository with the dataset identifier PXD039251.

### 5.11 Construction of Plasmids

Human *SPTAN1* (ENST00000630866.1, 2498 aa) and *SPTAN1* (NM_001195532, 2452 aa) were cloned into pCAG‐IRES‐GFP plasmid to get pCAG‐*SPTAN1‐1*‐IRES‐GFP and pCAG‐*SPTAN1‐2*‐IRES‐GFP, respectively. Mouse *Prx* (ENSMUST00000065487.7), human *DSP* (NM_004415), human *MPZ* (NM_000530), human *SCN9A* (NM_002977, 1977 residues), and mouse *Creb* (NM_009952) were cloned into pCAG‐IRES‐GFP plasmid to get pCAG‐*Prx*‐IRES‐GFP, pCAG‐*DSP*‐IRES‐GFP, pCAG‐*MPZ*‐IRES‐GFP, pCAG‐*SCN9A1*‐IRES‐mCherry, and pCAG‐*Creb*‐IRES‐GFP, respectively. Plasmids pCAG‐*SCN10A*‐IRES‐GFP (NM_006514.3), pCAG‐*SCN11A*‐IRES‐GFP (NM_001349253.2), and pCAG‐*SCN9A2*‐IRES‐mCherry (ENST00000642356.2, 1988 residues) were provided by Pengekiphen Company (Suzhou, China). The mouse promoters of *Dsp*, *Sptan1*, *Prx*, *Ahnak*, and *Mpz* were cloned into pGL4.17 vector, the cloned sequences are listed in Table S6.

### 5.12 Cell Culture and Transfection

HEK293 cells were cultured in DMEM medium containing 10% FBS, 1% penicillin and streptomycin at 37°C in an incubator supplied with 5% CO_2_. About 37,500 cells were suspended with mix of lipofectamine 2000 and 100 ng of each plasmid and then plated on PDL (Sigma, P6407) coated coverslip in a 48‐well plate. In first experiment, pCAG‐*SCN9A2*‐IRES‐mCherry or pCAG‐*SCN10A*‐IRES‐GFP was co‐transfected with pCAG‐*SPTAN1‐1*‐IRES‐GFP, pCAG‐*SPTAN1‐2*‐IRES‐GFP, pCAG‐*Prx*‐IRES‐GFP, pCAG‐*DSP*‐IRES‐GFP, and pCAG‐*MPZ*‐IRES‐GFP, respectively. In second experiment, pCAG‐*SCN9A1*‐IRES‐mCherry or pCAG‐*SCN9A2*‐IRES‐mCherry and pCAG‐*SCN10A*‐IRES‐GFP were cotransfected with pCAG‐*SPTAN1‐1*‐IRES‐GFP, pCAG‐*SPTAN1‐2*‐IRES‐GFP, pCAG‐*Prx*‐IRES‐GFP, pCAG‐*DSP*‐IRES‐GFP and pCAG‐*MPZ*‐IRES‐GFP, respectively. In antibody validation experiments, pCAG‐GFP, or pCAG‐*SCN9A1*‐IRES‐mCherry, or pCAG‐*SCN10A*‐IRES‐GFP, or pCAG‐*SCN11A*‐IRES‐GFP was transfected into HEK293 cells. Cells were fixed using 4% PFA 36 to 48 h post transfection, and then immunostained with antibodies, quantification data were derived from three independent experiments.

Before doing primary DRG neurons culture, confocal dish was coated with 0.1 mg/mL poly‐L‐lysine hydrobromide (P6282, Sigma, USA) in a cell culture incubator overnight, and then washed 3 times with sterile ddH_2_O, and dried at room temperature. L4‐6 DRGs from SNI mouse at 6 weeks post‐injury were harvested in cold Ham's F‐12K medium (PM150910, Procell, Wuhan, China) and any excess dorsal roots and spinal nerves were trimmed under a stereo microscope (Prior to dissection, all instruments were soaked in 70% ethanol). DRGs were digested in enzyme mix (3.55 mg/ml Dispase II (REF 04942078001, Roche, Japan) +1.65 mg/mL collagenase (C0130‐1G, Sigma, USA) + 0.125% Trypsin‐EDTA (REF 25200‐072, Gibico, Canada) in Ham's F‐12K medium) for 60 min at 37°C with gentle shaking every 5 min. After enzymatic digestion, equal volumes of Ham's F‐12K medium were added to terminate digestion. The digested cells were centrifuged at room temperature 5 min at 1000 rpm. Cell pellet was re‐suspended after discarding supernatant, and then cell suspension was planted to the center of each coated confocal dish and cultured at 37°C in an incubator supplied with 5% CO_2_ for 3 h.

### 5.13 Dual Luciferase Reporter Assay

HEK293 cells in 48‐well plate were co‐transfected with 50 ng/well pGL4.17‐*Dsp*‐promoter plasmid or pGL4.17‐*Sptan1*‐promoter plasmid, or pGL4.17‐*Prx*‐promoter plasmid, or pGL4.17‐*Ahnak*‐promoter plasmid, or pGL4.17‐*Mpz*‐promoter plasmid, or their mutant promoter plasmids, along with 5 ng/well pRL‐SV40‐renilla and 150 ng/well pCAG‐*Creb*‐IRES‐GFP or 150 ng/well pCAG‐IRES‐GFP empty vector, using Lipofectamine 3000 (Invitrogen, USA). Cells were lysed in passive lysis buffer 48 h post‐transfection, and Firefly and Renilla Luciferase luminescence were measured in a Victor luminometer (Wallac Sverige) using the Dual‐Luciferase Reporter Assay system (Promega, USA) according to the manufacturers' instructions. Firefly luminescence was normalized against Renilla luminescence for each well, and then the ratio of *Cre*‐transfected group was normalized to the ratio of empty vector‐transfected group. The normalized ratio was analyzed by unpaired Student's *t* test. Assays were performed in triplicate and data are derived from three independent experiments.

### 5.14 Protein‐Protein Docking Analysis

The predicted structures of human SPTAN1, MPZ, and AHNAK were retrieved from the AlphaFold Protein Structure Database (AF‐Q13813‐F1 for SPTAN1, AF‐P25189‐F1 for MPZ, and AF‐Q09666‐F1 for AHNAK) [97], whereas the experimentally determined structures of Na_v_1.7 (PDB 7W9K), Na_v_1.8 (PDB 9DBK), PRX (PDB 4CMZ), and DSP (PDB 3R6N) were obtained from the Protein Data Bank. Protein–protein docking was performed using the HADDOCK 2.4 web server (https://rascar.science.uu.nl/haddock2.4) [49]. The docking results suggested potential interactions of Na_v_1.8 with SPTAN1 (HADDOCK score = −108.5 ± 5.8, Z‐score = −2.8) and PRX (HADDOCK score = −65.0 ± 7.0, Z‐score = −2.2), Na_v_1.7 with SPTAN1 (HADDOCK score = −7.9 ± 15.7, Z‐score = −1.7), and DSP with SPTAN1 (HADDOCK score = 29.8 ± 26.2, Z‐score = −1.9), MPZ (HADDOCK score = 61.1 ± 28.1, Z‐score = −2.3), and AHNAK (HADDOCK score = 166.0 ± 44.7, Z‐score = −1.6). Among all tested docking pairs, Na_v_1.8–SPTAN1 yielded the most favorable docking result, with the lowest HADDOCK score and the most negative Z‐score.

A different docking strategy was used for the TRKB–DSP analysis because both proteins were represented as full‐length models and no experimentally resolved interface information was available to guide restraint definition. AF3‐DSP_HUMAN.pdb, representing full‐length DSP, was generated using AlphaFold 3, whereas the TRKB structure (AF‐Q16620‐F1) was retrieved from the AlphaFold Protein Structure Database. For docking of these two full‐length proteins with unknown binding interfaces, approximately 145 active residues were uniformly defined for each protein in HADDOCK. The top‐ranked cluster from the initial docking run, defined by the lowest HADDOCK score, was then used to infer putative interface regions for a refinement‐guided second round of docking. This procedure yielded a refined docking model with a HADDOCK score of −131.0 ± 22.2 and a Z‐score of −1.6. The resulting complex was downloaded and visualized using PyMOL (70).

### 5.15 Proximity Ligation Assay (PLA)

L5 DRGs collected from sham mice or SNI mice 2 weeks or 6 weeks after surgery were sliced using a cryostat at a thickness of 15 µm. The DRG sections were washed 3 times in PBS, blocked in proximity ligation blocking buffer (Sigma DUO92101) at room temperature and incubated with the following 7 pairs of primary antibodies in Duolink antibody diluent overnight at 4°C; 1) rabbit anti‐SPTAN1 (1:200; ProteinTech, 31676‐1‐AP) and mouse anti‐Na_v_1.8 (1:200; NeuroMab, 75–166) antibodies; 2) mouse anti‐SPTAN1 (1:250; Santa Cruz, sc‐46696) and rabbit anti‐Na_v_1.7 (1:200; ProteinTech, 20257‐1‐AP) antibodies; 3) Rabbit anti‐PRX (1:100; Abcam, Ab211292) and mouse anti‐Na_v_1.8 (1:200; NeuroMab, 75–166) antibodies; 4) mouse anti‐SPTAN1 (1:250; Santa Cruz, sc‐46696) and rabbit anti‐DSP (1:200; ProteinTech, 25318‐1‐AP) antibodies; 5) mouse anti‐DSP (1:100; Abcam, ab16434) and rabbit anti‐MPZ (1:50; Abcam, ab183868) antibodies; 6) rabbit anti‐ DSP (1:200; ProteinTech, 25318‐1‐AP) and mouse anti‐AHNAK (1:250; Santa Cruz, sc‐390743) antibodies; 7) goat anti‐TrkB (1:400; R&D systems, AF1494) and rabbit anti‐DSP (1:1000; ProteinTech, 25318‐1‐AP) antibodies. The negative control (without antibody) or single antibody control was also performed as Duolink PLA kit manufacturer's recommendations. After primary antibody incubation, DRG sections were then washed twice in wash buffer A at room temperature for 5 min. Anti‐rabbit PLUS (Sigma DUO92002) and anti‐mouse MINUS (Sigma DUO92004) or anti‐goat MINUS (Sigma DUO92006) probes were diluted 1:5 in Duolink antibody diluent and incubated with DRG sections for 1 h at 37°C. The DRG sections were then washed in wash buffer A at room temperature for 5 min twice and incubated with ligase diluted 1:40 in ligation buffer. After incubation at 37°C for 30 min, sections were washed twice with wash buffer A. Polymerase was diluted 1:80 in amplification buffer and incubated with sections for 100 min at 37°C. Sections were subsequently washed in wash buffer B twice for 10 min and mounted with Duolink In Situ mounting media with DAPI. Sections were imaged on a Zeiss LSM900. The average of PLA signals/cell normalized to negative controls from 20 cells/group was analyzed.

### 5.16 Knockdown shRNAs Screening and Generation of shRNA‐Expressing AAV Virions

The knockdown efficiency of three shRNAs targeting *Sptan1*, *Dsp*, *Ahnak*, *Mpz*, or *Prx* was evaluated by quantitative real‐time qRT‐PCR using primers listed in Table S6 (annealing temperature was 58°C). The assays were performed using cDNA derived from HEK293T cells which had been transiently co‐transfected with a truncated target gene cDNA and the respective shRNA constructs. Subsequently, the shRNA sequence exhibiting >70% knockdown efficiency was cloned into an AAV vector to produce shRNA‐expressing AAV virions (viral titer 5 × 10^12^) (BrainCase Co., Ltd., Shenzhen, China).

### 5.17 Injection of AAV Virions in DRG In Vivo

Virus injection in DRG was performed as described previously [98]. Briefly, 7–8 weeks old wild type C57BL/6 mice were anesthetized with isoflurane and L4 and L5 lumbar DRGs were exposed by removal of the lateral processes of the vertebrae. The epineurium over the DRG was opened, and the glass pipette with fine tip was inserted into the ganglion, to a depth of 100 µm from the surface of the exposed ganglion. After waiting 2 min to allow sealing of the tissue around the pipette tip, 0.7 µL of AAV2/9 virions (viral titer 5 × 10^12^, Scrambled ShRNA‐expressing virus, targeting‐*Prx* shRNA‐expressing virus (shRNA *Prx*), targeting‐*Ahnak* shRNA‐expressing virus (shRNA *Ahnak*), targeting‐*Sptan1* shRNA‐expressing virus (shRNA *Sptan1*), targeting‐*Dsp* shRNA‐expressing virus (shRNA *Dsp*), targeting‐*Mpz* shRNA‐expressing virus (shRNA *Mpz*) or Mix of five viruses) was injected into DRGs of C57BL/6 mice at a rate of 0.1 µL/min using microprocessor controlled minipump (RWD Life Science, China). The pipette was removed after a further delay of 5 min. The muscles overlying the spinal cord were carefully sutured and mice were allowed to recover at 37°C warming blanket. SNI surgery was performed 5 days after AAV virus injection. Three independent cohorts of mice were used. In the first cohort, *WT* mice received only AAV‐virus injection, and knockdown efficiency of shRNAs was assessed by extracting total RNA from L4–L5 DRG using TRIzol Reagent (Thermo Fisher Scientific, MA, USA), followed by reverse transcription with the RevertAid First Strand cDNA Synthesis Kit (Thermo Fisher Scientific, MA, USA, K1621). Real‐time quantitative PCR was performed using SYBR Green on an ABI QuantStudio 3 system (Thermo Fisher Scientific, MA, USA) with primers listed in Table S6 and 18S rRNA as internal control. In the second cohort, mice were allowed to recover for 6 weeks after SNI before behavioral tests and patch‐clamp analysis were conducted. In the third cohort, behavioral tests were performed at 4, 6, and 8 weeks post‐recovery.

### 5.18 Intact Whole‐Mount DRG Preparations and Whole‐Cell Patch Clamp Recording

As described previously [99], mouse L4/L5 DRGs and human cervical DRG were carefully removed and placed into artificial cerebrospinal fluid (ACSF). After removing the connective tissue, the ganglia were digested with a mixture of 0.4 mg/mL trypsin (Sigma, USA) and 1.0 mg/mL type‐A collagenase (Sigma, USA) for 30 min at 37°C. The intact ganglia were then incubated in ACSF oxygenated with 95% O_2_ and 5% CO_2_ at 28°C for at least 1 h before transferring them to the recording chamber. The ACSF contained (in mm): 125 NaCl, 2.5 KCl, 1.2 NaH_2_PO_4_, 1.0 MgCl_2_, 2.0 CaCl_2_, 26 NaHCO_3_, and 10 glucose. The patch pipettes (resistance of 3–7 MΩ) were filled with intracellular solution for DRG recording contained the following (in mm): K‐gluconate, 126; NaCl, 10; MgCl_2_, 1; EGTA, 10; NaATP, 2, and MgGTP, 0.1, adjusted to pH 7.4 with KOH and osmolarity 295–300 mOsm. To accurately measure voltage‐clamp sodium currents, the intracellular solution contained the following (in mm):CsCl 110, NaCl 5, MgCl_2_ 3, CaCl_2_ 1, EGTA 3, and HEPES 40, adjusted with Tris buffer to pH 7.4. The external solution was composed of (in mm): NaCl 90, choline chloride 30, TEACl 20, CaCl_2_ 0.1, MgCl_2_ 5, CoCl_2_ 5, HEPES 10, glucose 10 adjusted to pH 7.4 with NaOH. DRG neurons were visualized with a 40× water‐immersion objective using a microscope (BX51WI; Olympus, Tokyo, Japan) equipped with infrared differential interference contrast optics. Membrane potential was held at −60 mV under voltage‐clamp mode. Depolarizing current steps (500 ms in duration and 50 pA increments) were used to detect the action potential (AP). The AP threshold was determined by differentiating the AP waveform and setting a rising rate of 10 mV/ms as the AP inflection point. The effects of drugs were detected by bath application of 10 µm Na_v_1.7 blocker PF‐05089771 or 2.5 µm Na_v_1.8 blocker PF‐04885614, or the combination which contains 10 µm Na_v_1.7 blocker and 2.5 µm Na_v_1.8 blocker, or 500 nm TrkB agonist 7, 8‐Dihydroxyflavone, or 100 µm TrkB antagonist ANA‐12. Whole‐cell current and voltage recordings were acquired with an Axon700B amplifier (Molecular Devices Corporation) and pCLAMP10.0 software. Signals were low‐pass filtered at 5 kHz, sampled at 10 kHz and analyzed offline.

In voltage‐clamp experiments, the transient sodium current (I_Na_) was evoked by a test pulse to +0 mV from the holding potential, −60 mV. The persistent sodium current (I_NaP_) was recorded by applying a 3 s depolarization ramp current from −80 to −20 mV at a holding potential of −60 mV [100]. Membrane potential was held at −60 mV under voltage clamp. Neurons that showed resting membrane potentials below −50 mV along with overshooting action potentials were selected for further study. Leak subtraction was performed online using a P/4 protocol with 4 subpulses of opposite polarity. Series resistance was maintained below 20 MΩ with voltage errors minimized by 70–80% series resistance compensation in the whole‐cell configuration.

### 5.19 Recording of Potential in SMAC Area Using the Potential Indicator ASAP3

ASAP3 fluorescence is a good potential indicator and can well reflect the change of potential in subcellular zone in neurons responding to stimuli [54, 101]. 0.8 µL of rAAV‐hSyn‐ASAP3‐WPRE‐hGH polyA (BrainVTA, China, the sequence of ASAP3 was previous reported by Villette et al. [54]) was injected into the L4 and L5 DRG of SNI *TrkB^2A‐Tomato/+^* mice at 2 weeks post‐injury as described above. Animals were used at 4 weeks after AAV injections. Whole‐mount L4‐L5 DRG from SNI *TrkB^2A‐Tomato/+^* mice at 6 weeks post‐injury were prepared as described above, and then transferred to the imaging chamber containing extracellular imaging buffer. DRG were visualized with infrared differential interference contrast (DIC) illumination with 1.0‐NA water‐immersion and fluorescence signal of ASAP3 (green) were acquired at frequency of 73.6 Hz (14.58 ms/frame) with a line scanning mode across a cell using two‐photon laser‐scanning microscope (FVMPE‐RS, Olympus) equipped with a mode‐locked tunable (690–1040 nm) Mai Tai eHPDS Ti:sapphire laser (Spectra‐Physics) tuned to 920 nm. Signals recorded along each line were integrated to produce a fluorescence trace over time. Tomato‐expressing neurons with SMAC were selected and whole‐cell current‐clamp recording was performed using a Multiclamp 700B amplifier (Molecular Devices) with the pCLAMP11.0 software and borosilicate glass microelectrodes (3‐7 MΩ) filled with an internal solution (K‐gluconate, 126; NaCl, 10; MgCl2, 1; EGTA, 10; NaATP, 2 and MgGTP, 0.1, pH 7.4, 295–300 mOsm). To induce DRG firing, 100 or 300‐ms pulses of 200∼500‐pA current was injected to induce spiking (5 pulses with interval 5 s). Potential signals were filtered with a Bessel filter at 2 kHz to eliminate high frequency noise, digitized at 10 kHz. Time array images of ASAP3 signal were exported from OlyVIA software and analyzed using ImageJ. A frame of ASAP3 image was divided into 36 slices (a TrkB neuron covered about 26–32 slices), and the SMAC area and non‐SMAC area on each slice were selected with box tool according to Tomato fluorescence signal and the ASAP3 fluorescence intensity value of the selected area on each slice from stack images was measured and exported from ImageJ to an excel file. To quantify the change of ASAP3 fluorescence signals, the change of fluorescence intensity (△F) in each selected area from each frame was normalized to the average (F) of 2 s baseline fluorescence signals. The data (△F/F) from the slice which was photographed during the occurrence of peak of first action potential at each pulse stimuli was picked up, and the average of five these data (each cell was stimulated for 5 programmed‐pulses) presented the change of ASAP3 fluorescence signal (potential) within SMAC area or non‐SMAC area in parallel with action potential response to the stimuli.

### 5.20 Recording of Na^+^ Concentration and Action Potential in Parallel

Whole‐mount L4‐L5 DRG from SNI *TrkB^2A‐Tomato/+^* mice at 6 weeks post‐injury were prepared as described above, and then were incubated with 1 µm Na^+^ indicator, CoroNa Green (AM, C36676, Thermo Fisher Scientific). Whole‐cell current‐clamp recording and two‐photon imaging were done as described above, except the frequency of image acquiring was 76 Hz (the size of frame was smaller). For combo treatment, 5 µL combo of PF‐05089771 (10 mm) and PF‐04885614 (2.5 mm) was added into 5 mL imaging buffer without stirring after the neuron was recorded for 5 times of programmed‐pulses stimuli. The same neuron was recorded from the time point of application of combo till no spike could be induced by 5 times of programmed‐pulses stimuli. The frame of Na^+^ indicator image was divided into 32 slices and the fluorescence intensity of SMAC area and non‐SMAC area in each slice was measured using ImageJ and exported to an excel file. The fluorescence traces in 2 s baseline were used to get a trend line in excel, and then the fluorescence traces were corrected for photobleaching according to the formula of the trend line. Afterward, the change of Na^+^ indicator signal within SMAC area and non‐SMAC area was quantified and presented in the same way as ASAP3 signal described above. The information of reagents, antibodies, software, equipment is collectively listed in Table S7.

### 5.21 Statistical Analysis

All electrophysiological data were shown as individual raw data points with mean and standard deviation (mean ± SEM) and other numerical data were shown as individual raw data points with mean and standard deviation (mean ± SD) and analysed by GraphPad Prism 8 software (GraphPad Software Inc, CA, USA) The D'Agostino‐Pearson omnibus normality test was performed before further analysis. The non‐parametric Kruskal‐Wallis test was used when the data were not normally distributed. The numerical data of immunostaining results between 2 groups like sham and SNI mice were analysed by unpaired Student's *t* test. A value of *p* < 0.05 was considered as statistically significant. The detailed information including sample size and statistical methods for each figure was listed in “statistic reporting”‐Table S8.

## Funding

This work was supported by grants from the National Natural Science Foundation of China (32070977, 51971236, and 31871063 to C.G.P.; 82101320 to L.T.S.; 82171212, 82371225 and 82571386 to R.G.X.; 82302681 to H.X.; 8213012, 82293640, and 82293643 to L.Z.X.; 82060149 to H.Y.S.; 82221001 to S.X.W.), from the Major National Science and Technology Projects of China (2018ZX09733001‐006‐005 to C.G.P.), from the Ministry of Science and Technology of China (2021ZD0203205 to R.G.X.), grants from Excellent Youth Science Foundation of Shaanxi Province (Grant No. 2025JC‐JCQN‐103) to R.G.X., from Scientific and Technological Innovation 2030—major project of Brain Science and Brain‐Like Intelligence Technology (2021ZD0202804 to L.Z.X.), and from The second round of the three‐year action plan for “strengthening and promoting Traditional Chinese Medicine” of Hongkou District [HKGYQYXM‐2022‐06] to L.Z.X, and grants from Shanghai Fourth People's Hospital (Sykyqd11201, SY‐SKZT‐2025‐1003) to C.G.P. *Scn9a^HA^* mice were supplied by the Genome Tagging Project Center at the Institute of Biochemistry and Cell Biology of the Chinese Academy of Sciences (Shanghai, China), which was supported by the Shanghai Municipal Commission for Science and Technology (19411951800).

## Conflicts of Interest

The authors declare that they have competing interests. Part of data in Figures 4, 9, S4, and S9 from patent applications (PCT/CN2021/072071, ZL202010845095.2, and CN202210052495.7) authored by C.G.P., L.T.S., R.L.X., and R.G.X.

## Supporting information




**Supporting File 1**: advs75466‐sup‐0001‐SuppMat.docx.

**Supporting File 2**: advs75466‐sup‐0002‐TableS2.xlsx.

**Supporting File 3**: advs75466‐sup‐0003‐TableS3.xlsx.

**Supporting File 4**: advs75466‐sup‐0004‐TableS4.xlsx.

**Supporting File 5**: advs75466‐sup‐0005‐TableS5.xlsx.

**Supporting File 6**: advs75466‐sup‐0006‐TableS8.xlsx.

**Supporting File 7**: advs75466‐sup‐0007‐MovieS1.mp4.

**Supporting File 8**: advs75466‐sup‐0008‐MovieS2.mp4.

**Supporting File 9**: advs75466‐sup‐0009‐MovieS3.mp4.

**Supporting File 10**: advs75466‐sup‐0010‐MovieS4.mp4.

**Supporting File 11**: advs75466‐sup‐0011‐MovieS5.mp4.

**Supporting File 12**: advs75466‐sup‐0012‐MovieS6.mp4.

**Supporting File 13**: advs75466‐sup‐0013‐MovieS7.mp4.

**Supporting File 14**: advs75466‐sup‐0014‐MovieS8.mp4.

**Supporting File 15**: advs75466‐sup‐0015‐MovieS9.mp4.

**Supporting File 16**: advs75466‐sup‐0016‐MovieS10.mp4.

**Supporting File 17**: advs75466‐sup‐0017‐MovieS11.mp4.

**Supporting File 18**: advs75466‐sup‐0018‐MovieS12.mp4.

**Supporting File 19**: advs75466‐sup‐0019‐MovieS13.mp4.

**Supporting File 20**: advs75466‐sup‐0020‐MovieS14.mp4.

**Supporting File 21**: advs75466‐sup‐0021‐MovieS15.mp4.

## Data Availability

All data are available in the main text or the supplementary materials. The mass spectrometry proteomics data have been deposited to the ProteomeXchange Consortium via the PRIDE partner repository with the dataset identifier PXD039251.
