# Supplementary material for: Targeting Supramolecular Active Complexes of Nav1.7/Nav1.8 to Relieve Chronic Neuropathic Pain
Source: Adv Sci (Weinh). 2026 May 20;13(41):e22185. doi: 10.1002/advs.202522185 (PMC13335541; doi:10.1002/advs.202522185)
Supplement: Supplementary file 1 — Supporting File 1: advs75466‐sup‐0001‐SuppMat.docx. [file ADVS-13-e22185-s021.docx]

Supporting Information

Targeting Supramolecular Active Complexes of Na_v_1.7/Na_v_1.8 to Relieve Chronic Neuropathic Pain

*Liting Sun ^1, †, Ψ^, Hang Xian ^2,3, †^, Yunxin Shi ^1,2, †^, Taotan Yang ^1,4, †^, Hongyan Shuai ^5, †^, Wenchao Hu^2^, Siying Fei ^1^, Miao Xu ^1, 6^, Taoyuan Yang ^1^, Ruilong Xia ^1^, Ting Wen ^1^, Fengting Zhu ^1,5^, Yan Fu ^1^, Yang Li ^1^, Wei Xia ^1^, Ran Qian ^1^, Yuanying Liu ^2^, Zhicheng Tian ^2^, Lamei Li ^2^, Qian Zhou^1^, Lize Xiong ^6^, Rui Cong ^3^, Ceng Luo ^2^, Shengxi Wu ^2^, Xiafeng Shen ^1,^ ^*^, Xin Yu ^5,^ ^*^, Rou-Gang Xie ^2,7^ ^*^, Changgeng Peng ^1,6^ ^*^*

**This PDF file includes:**

Figure S1 to 27

Table S1, S6 and S7

Description of Table S2 to S5 (Separate excel file)

Description of Video S1 to S15

References (1 to 8) (if applicable—these should refer only to references in the SM)

**Other Supplementary Materials for this manuscript include the following:**

Movies S1 to S15

Data S2 to S5

Statistic data


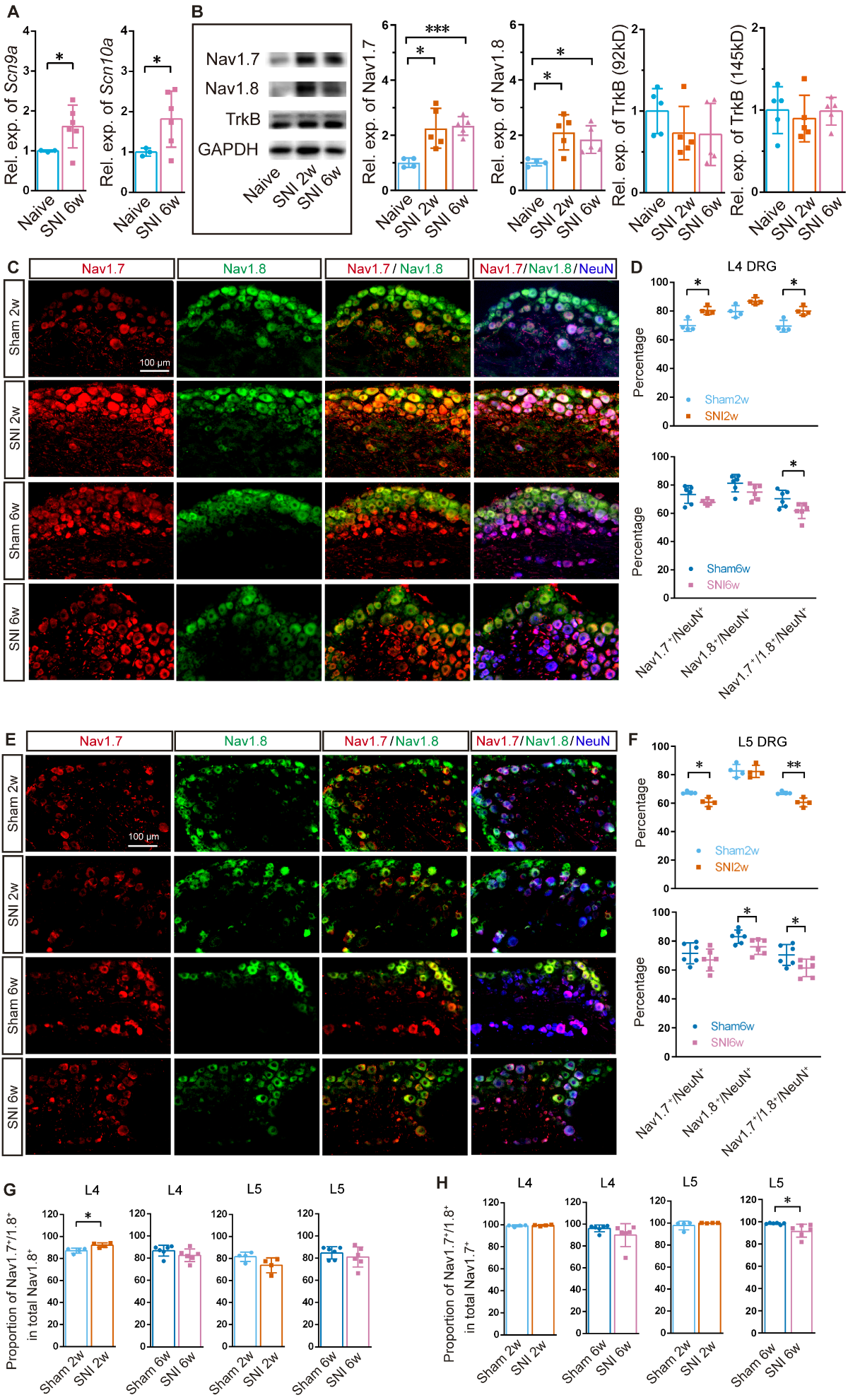
Figure S1. Increased expression of Na_v_1.7 and Na_v_1.8 in L4-L6 DRGs of SNI mice at 2 weeks or 6 weeks post-injury. (A) Quantitative RT-PCR showed the relative expression levels of *Scn9a* and *Scn10a* in L4-L6 DRGs of naïve mice and SNI mice at 6 weeks post-injury. (B) The protein levels of Na_v_1.7, Na_v_1.8, and TrkB in L4-L6 DRGs of naïve mice and SNI mice at 2 weeks and 6 weeks post-injury were measured by Western-blot assay and normalized to the expression level of GAPDH. (C, D) Quantitative analysis of L4 DRG sections immunostained with antibodies against (C) Na_v_1.7 (red), Na_v_1.8 (green) or NeuN (blue). Percentages of Na_v_1.7^+^, Na_v_1.8^+^ and Na_v_1.7^+^/ Na_v_1.8^+^ neurons in L4 DRG of sham and SNI mice at 2 weeks and 6 weeks post-injury (D). (E, F) Immunostaining of L5 DRG against Na_v_1.7 (red), Na_v_1.8 (green) and NeuN (blue) in sham and SNI mice, and the percentages of Na_v_1.7^+^, Na_v_1.8^+^ and Na_v_1.7^+^/ Na_v_1.8^+^ neurons in L5 DRG of sham and SNI mice. (G) Proportions of Na_v_1.7^+^/Na_v_1.8^+^ neurons among total Na_v_1.8^+^ neurons in L4 or L5 DRG of sham and SNI either at 2 or 6 weeks post-injury. (H) Proportions of Na_v_1.7^+^/Na_v_1.8^+^ neurons among total Na_v_1.7^+^ neurons in L4 or L5 DRG of sham and SNI mice at 2 weeks or 6 weeks post-injury. Scale bar=100 μm, * *p*<0.05, ** *p*<0.01, *** *p*<0.001, unpaired Student’s *t* test, or Mann Whitney test, or Brown-Forsythe ANONA test, or Ordinary one-way ANOVA with Tukey's multiple comparisons test, n=4-6 animals per condition.


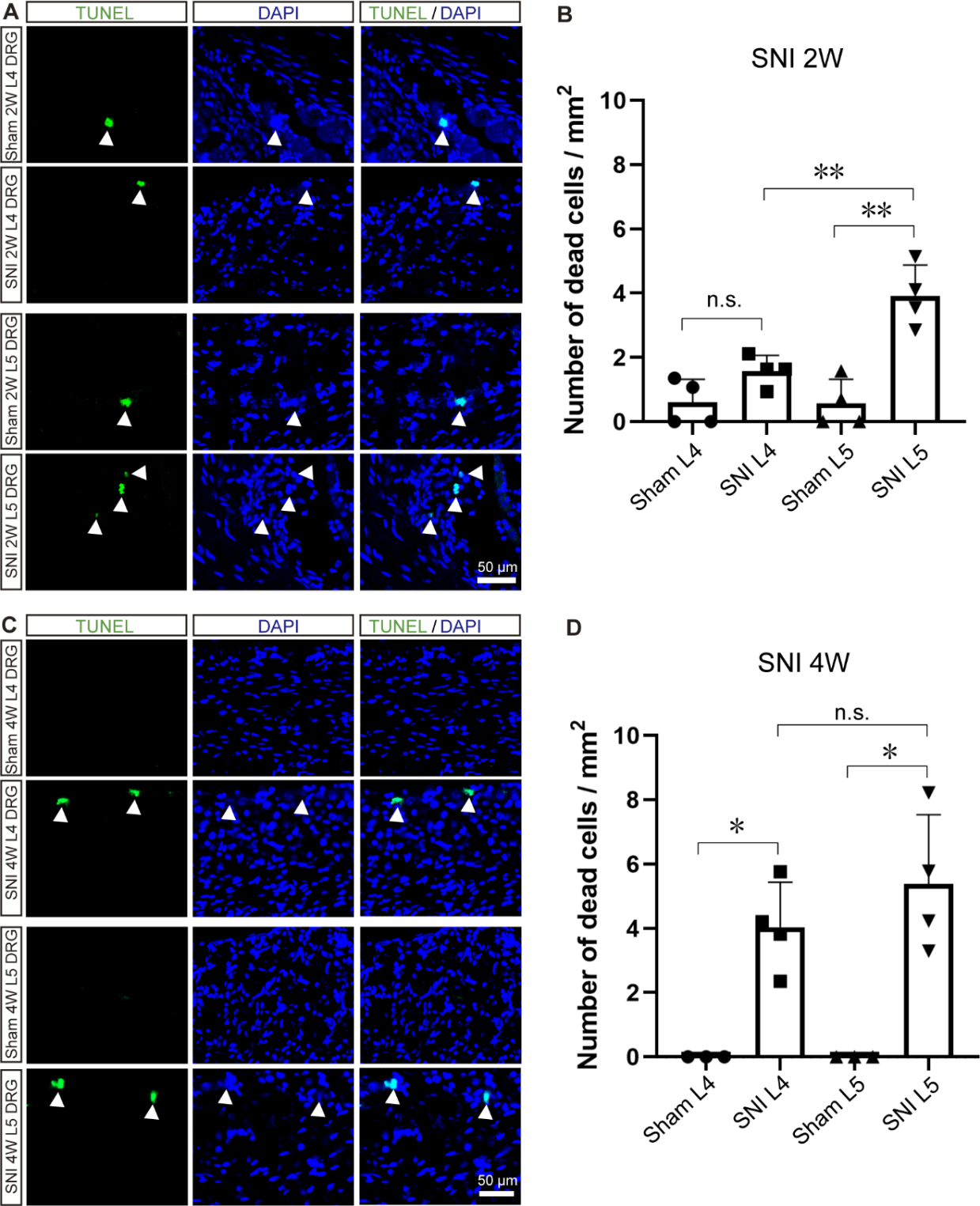
Figure S2. Increased neuronal apoptosis in DRG of SNI mice. (A) TUNEL (Green) and DAPI double-staining of L4-5 DRG sections from sham and SNI mice at 2 weeks after injury. (B) Quantification of apoptotic neurons in L4-5 DRG in sham and SNI mice at 2 weeks after injury. (C) TUNEL (Green) and DAPI double-staining of L4-5 DRG sections from sham and SNI mice at 4 weeks post-injury. (D) Quantification of apoptotic neurons in L4-5 DRG in sham and SNI mice at 4 weeks post-injury. Scale bar=50 μm, * *p*<0.01, ** *p*<0.01, Brown-Forsythe ANONA test with Dunn's multiple comparisons test, n=4 animals per condition. n.s.: not significant Arrowheads pointed to TUNEL signal positive cells.


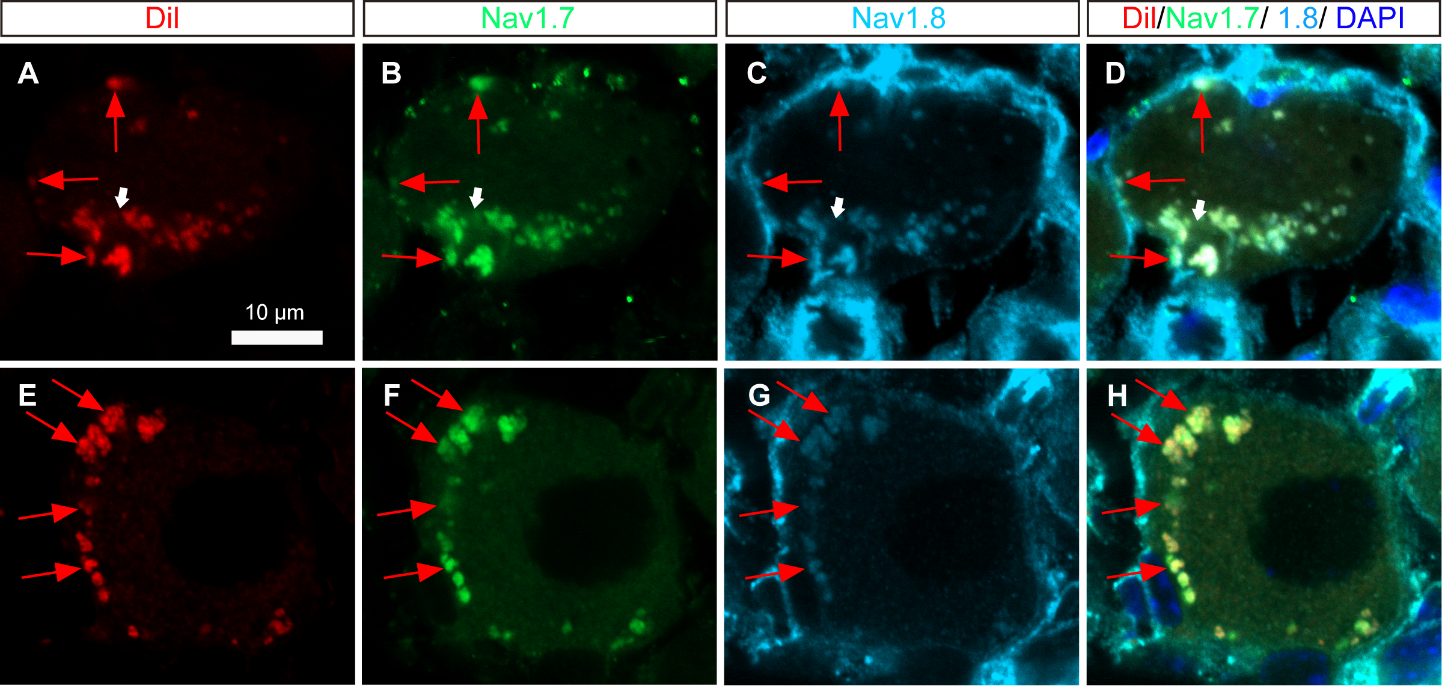


**Figure S3.** **Na_v_1.7/Na_v_1.8 SMAC located on DRG neuron surface 6 weeks post-SNI injury.** **(A-D)** Fixed sections of DRG derived from SNI mice at 6 weeks post-injury were immunostained with Dil (A, red), a plasma membrane dye, along with Na_v_1.7 (B, green) and Na_v_1.8 (C, light blue), and counterstained with DAPI (D). **(E-H)** Dil (E) was injected into L5 DRG of mice at 6 weeks post-injury, and the DRG was dissected 1 hour later. Cryosections of L5 DRG were then immunostained for Na_v_1.7 (F, green) and Na_v_1.8 (G, light blue), with DAPI (H) as a nuclear counterstain. Notice: Dil labelled SMAC on DRG neuronal membrane was tightly connected to satellite glial cells which were recently found to play an important role in NP too [1-2]. Red arrows point to clusters of SMAC on plasma membrane, white arrows point to axon base.


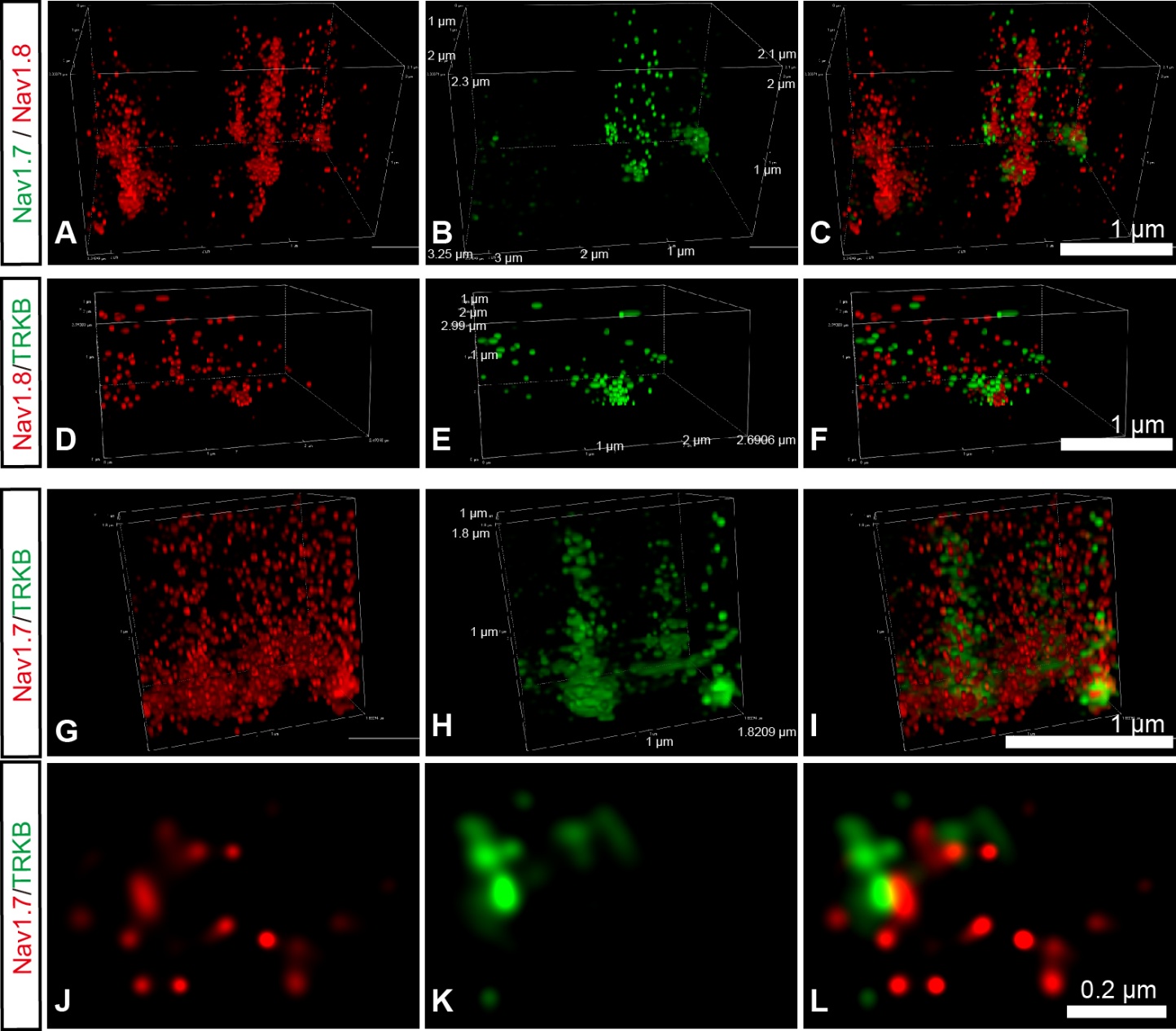


**Figure S4. 3D STORM images of Na_v_1.7/Na_v_1.8 SMAC in primary cultured DRG neurons from SNI mice at 6 weeks post-injury. (A-C)** 3D STORM images of immunostaining against Na_v_1.8 (A, Red) and Na_v_1.7 (B, Green), and merged channels (C). **(D-F)** 3D STORM images of immunostaining against Na_v_1.8 (D, Red) and TrkB (E, Green), and merged channels (F). **(G-I)** 3D STORM images of immunostaining against Na_v_1.7 (G, Red), and TrkB (H, Green), and merged channels (I). **(J-L)** A slice view of 3D STORM image of immunostaining against Na_v_1.7 (J, Red), TrkB (K, Green), and merged channels (L).


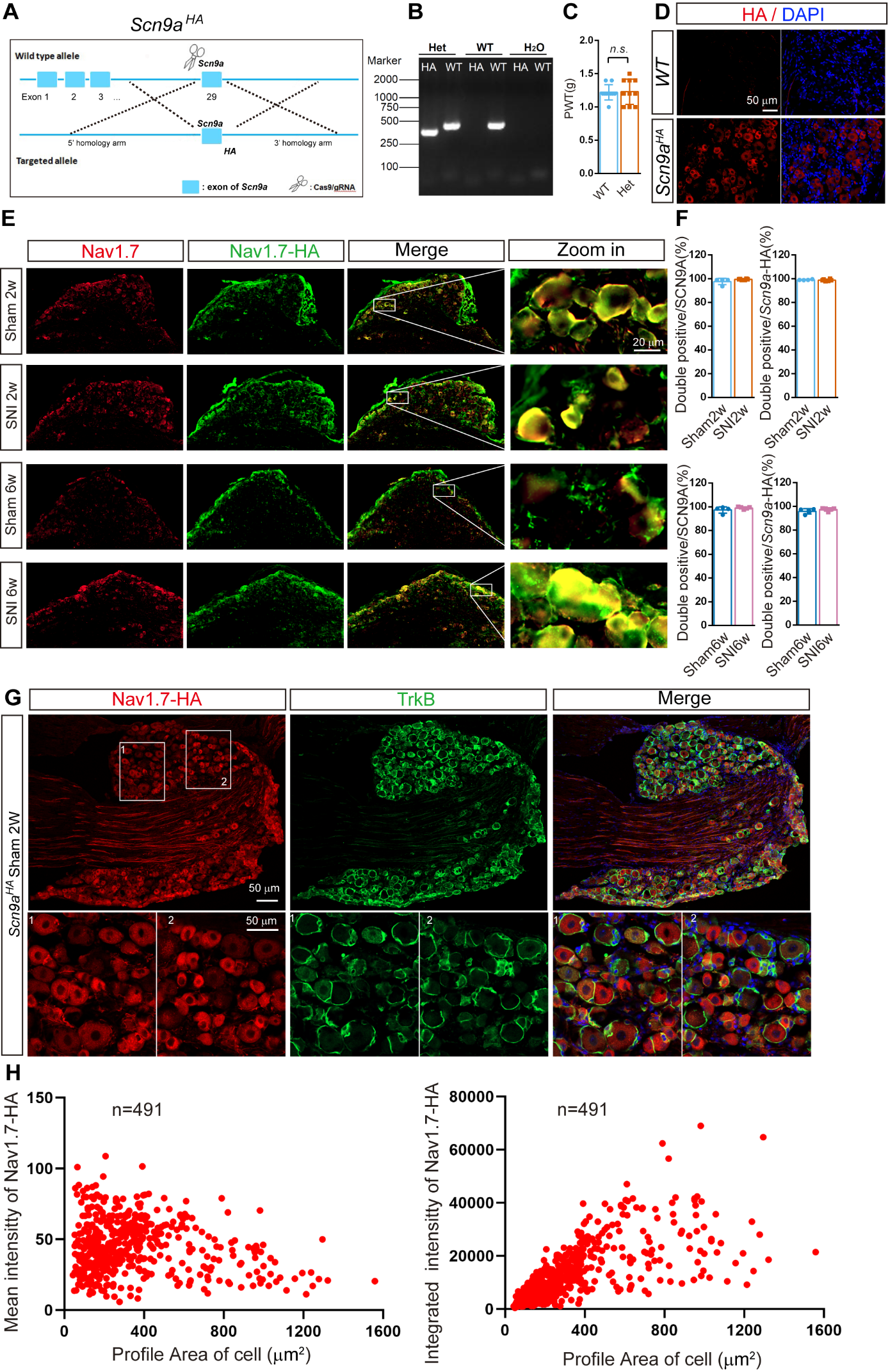


Figure S5. Generation and validation of *Scn9a^HA/+^* mice. (A) Schematic view of *wild-type* (*WT*) and targeted *Scn9a* alleles in mice. Hemagglutinin (HA) tag was inserted upstream of the stop codon of exon 29 in *Scn9a* (NM_001290675.1)*.* (B) Genotyping PCRs with two primer pairs to recognize *WT* and *HA* knocked-in alleles. (C) Mechanical pain thresholds of *Scn9a^HA/+^* mice and *WT* littermates (n=10 animals per group). (D) Immunostaining for HA (red) on DRG sections from *WT* (upper panel) and *Scn9a^HA/+^* (lower panel) mice, with nuclei counterstained with DAPI (blue). (E) Triple staining against HA (red), Na_v_1.7 (green) and DAPI (blue) in DRG sections from sham and SNI mice at 2 and 6 weeks post-injury. (F) Proportions of Na_v_1.7-expressing cells in DRG co-labelled by antibody against HA and antibody against Na_v_1.7 from sham and SNI mice at 2 weeks post-injury (n=4-6 animals per group) and 6 weeks post-injury (n=4 animals per group). Ratios of the number of double-positive cells to the number of Na_v_1.7-positive cells were as follows: sham 2 weeks post-injury, (97.6% ± 2.69%); sham 6 weeks post-injury, 97.3%± 2.77%; SNI 2 weeks post-injury, 99.4% ± 0.41%; and SNI 6 weeks post-injury, 99.1% ± 0.93%. Ratios of the number of double-positive cells to the number of Na_v_1.7-HA positive cells were as follows: sham 2 weeks post-injury, 99.0% ± 0.33%; sham 6 weeks post-injury, 95.8% ± 2.23%; SNI 2 weeks post-injury, 98.76 %± 0.77%; and SNI 6 weeks post-injury, 97.4 %± 1.38%. (G) Triple staining for HA (red), TrkB (green) and DAPI (blue) on DRG sections from sham *Scn9a^HA/+^* mice at 2 weeks post-injury showed co-expression Na_v_1.7 and TrkB in DRG neurons. (H) Quantification of DRG neurons in (G) showed that Na_v_1.7 was widely expressed across all neuron sizes, with varying expression levels within each size category (left graph), in line with single-cell sequencing data. Notably, medium-to large-DRG neurons exhibit the highest total Na_v_1.7 protein level (integrated intensity), although some small sized DRG neurons show the highest Na_v_1.7 intensity. *n.s.*: not significant, unpaired *t* test with Welch's correction or Mann-Whitney test.


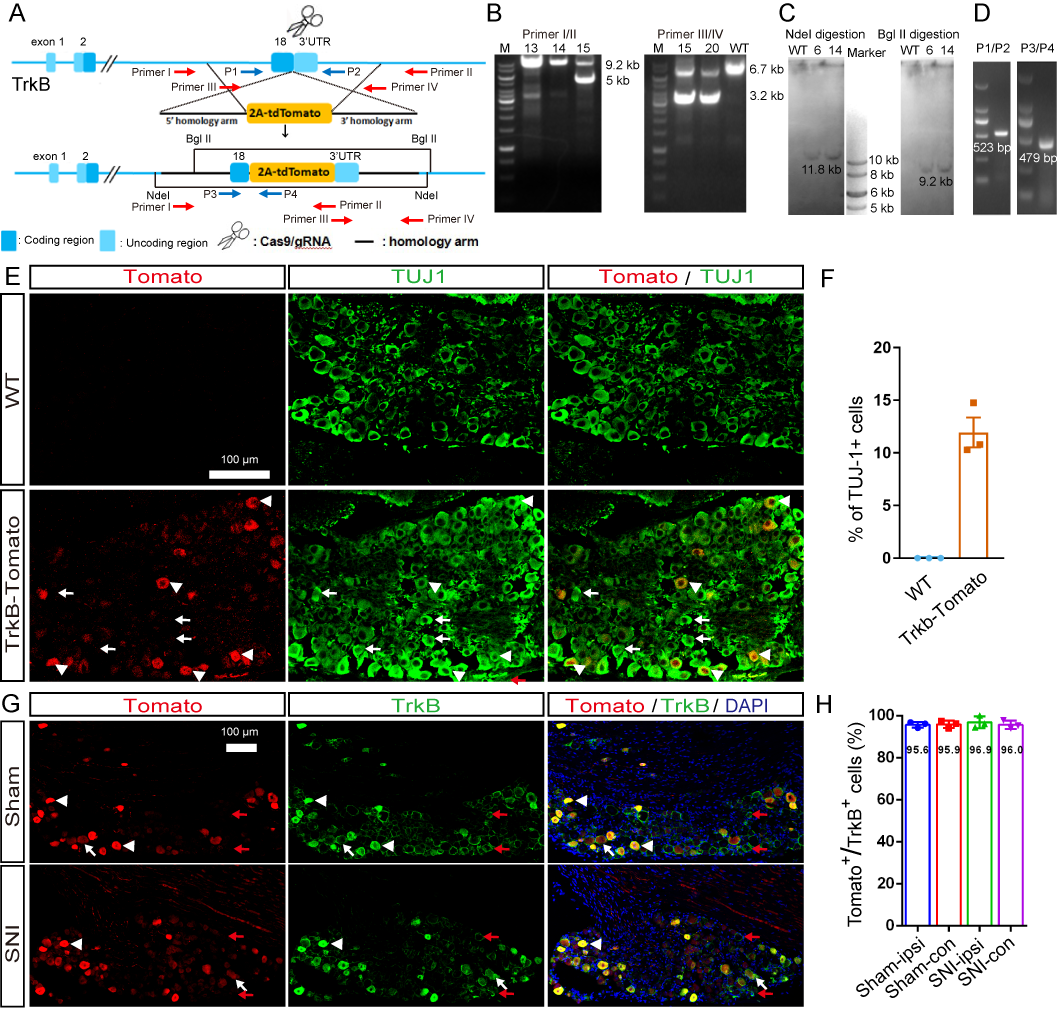
Figure S6. Generation and validation of *TrkB^2A-Tomato/+^* mice. (A) Schematic view of *WT* and targeted *TrkB* alleles, with the location of primers. *TrkB^2A-Tomato/+^* mice were generated using CRISPR/Cas9 technology. (B) The targeted allele in F0 and F1 mice was detected using PCR with two primer pairs: primers I and II in panel A gave a 9.2-kb band for the targeted allele, while primers III and IV in panel A gave a 6.7-kb band for the targeted allele. These results were confirmed by Sanger sequencing. (C) In correctly generated mice, Southern blotting of DNA from mouse tail with *Tomato* probe detected an 11.8-kb band after *Nde*I digestion and a 9.2-kb band after Bgl II digestion. (D) Two primer pairs were used to genotype the *WT* allele (P1/P2, a 523-bp band) and the targeted allele (P3/P4, a 479-bp band) of *TrkB*. (E) Immunostaining against Tomato and TUJ1 showed that some neurons expressed Tomato in *TrkB^2A-Tomato/+^* mice，not in *WT* mice. Arrowheads point to Tomato^+^/TUJ1^+^ neurons, and arrows point to Tomato^-^/ TUJ1^+^ neurons. (F) Quantitation showed that all Tomato^+^ were TUJ1^+^ neurons, while Tomato labelled 10.9 ± 3.0% of TUJ1^+^ neurons (n=4 animals), consistent with the reported percentage of LTMR TrkB neurons [3]. (G) Immunostaining against Tomato and TrkB demonstrated that all Tomato^+^ neurons were TrkB^+^ cells, and that there were neurons expressing low or high levels of Tomato in DRG of sham and SNI mice at 2 weeks post-injury. Arrowheads point to TrkB^+^ neurons expressing high levels of Tomato^+^; white arrows, to TrkB^+^ neurons expressing low levels of Tomato^+^; and red arrows, to TrkB^+^ neurons not expressing detectable Tomato. (H) Quantitation showing that approximately 96% LTMR TrkB neurons (these neurons containing TrkB in cytoplasmic) expressed Tomato in ipsilateral and contralateral DRG of sham and SNI mice. Scale bar=100 μm, n=3-4 animals per condition.


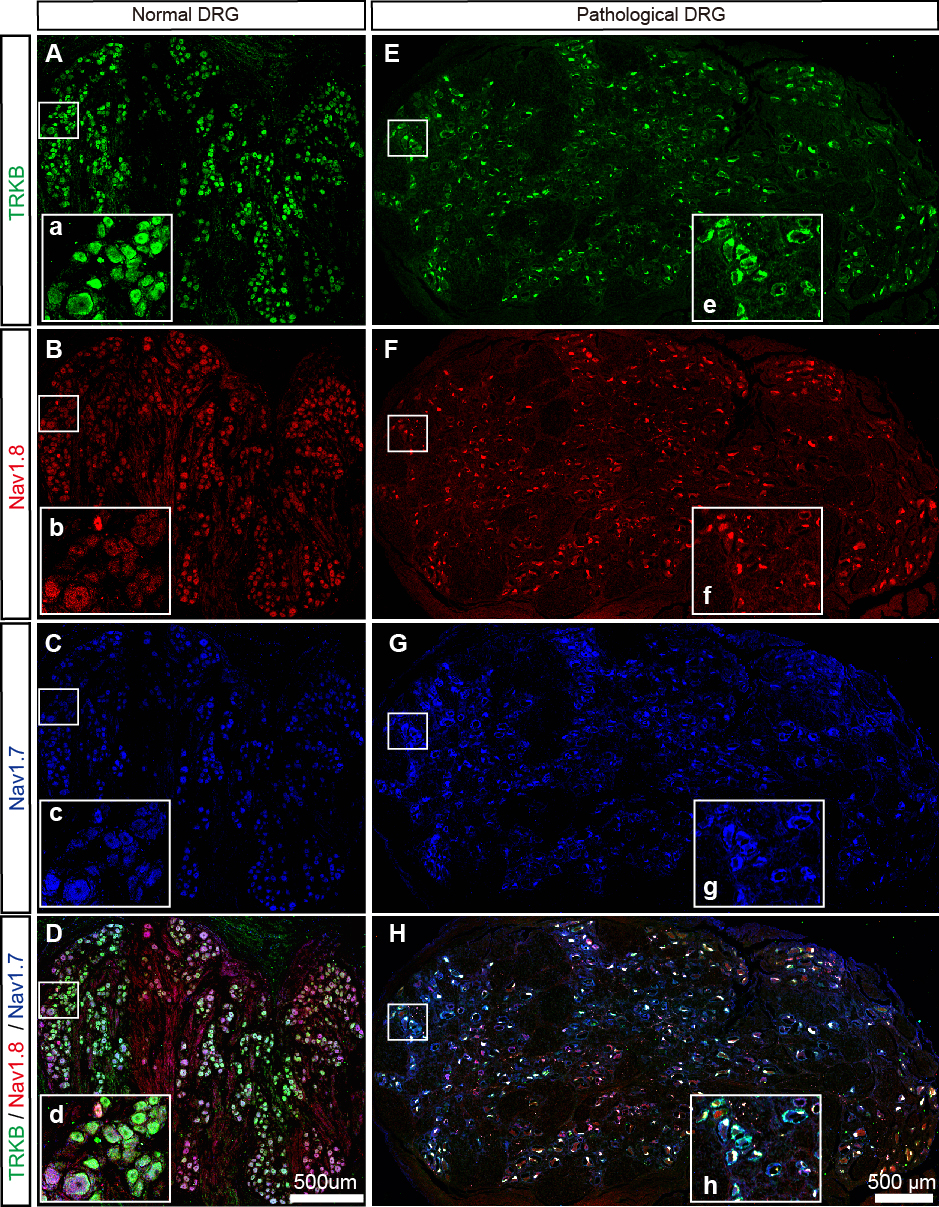
Figure S7. Elevated expression of Na_v_1.8 in Na_v_1.7^+^/TRKB^+^ neurons in DRG of patients with neuropathic pain (NP) after brachial plexus avulsion. Immunostaining against Na_v_1.7 (blue), Na_v_1.8 (red) and TRKB (green) in DRG sections from normal DRG from human embryos naturally aborted at 29 weeks (A-D) and from patients with NP after brachial plexus avulsion (E-H) showed that DRG from patients contained more TRKB/Na_v_1.7 neurons expressing high levels of Na_v_1.8 than normal DRG from human embryos. Panels a-h are higher-magnification views of the boxed small region (top left) in panels A-H, respectively. Scale bar=500 μm.


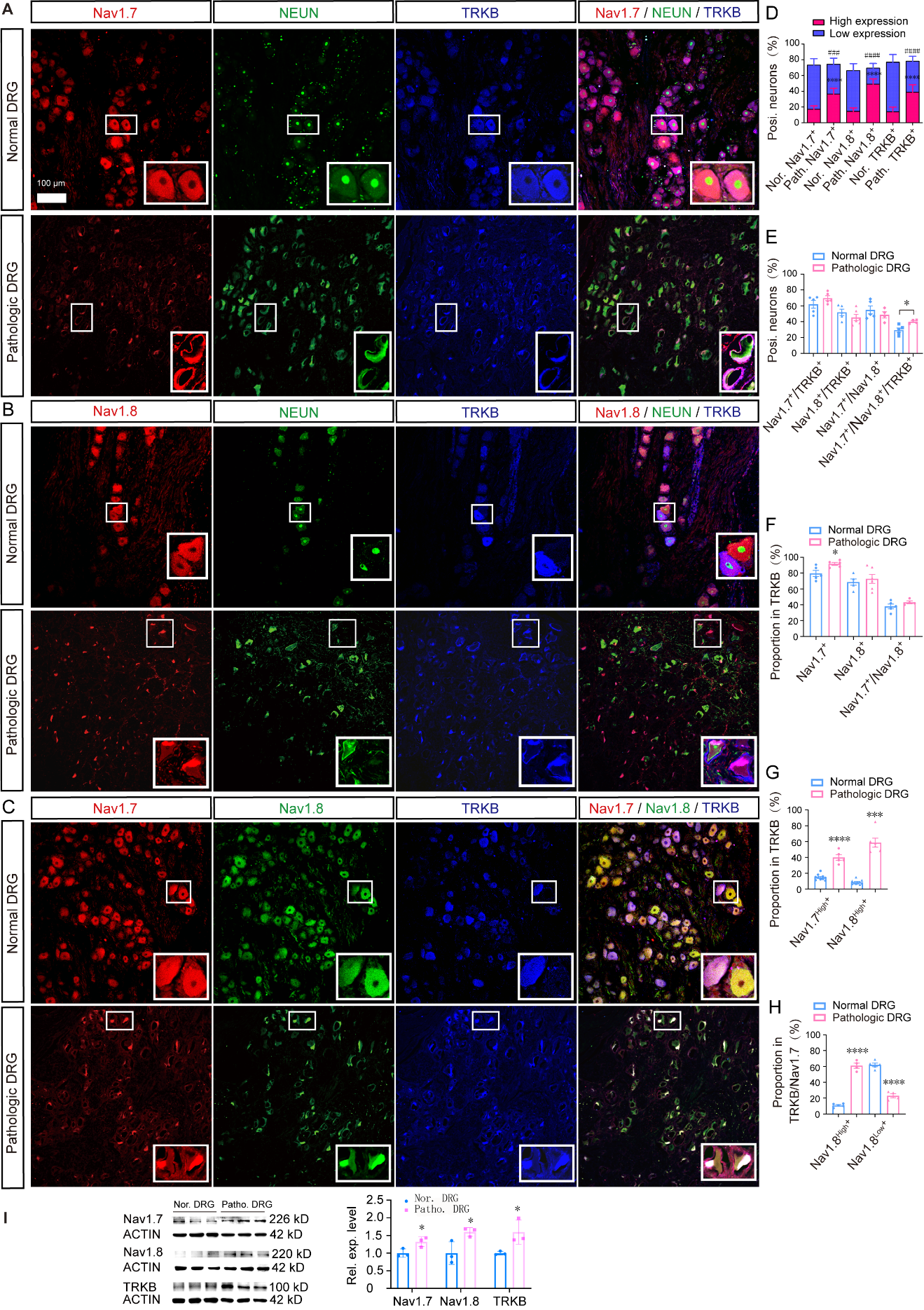
Figure S8. Upregulation of Na_v_1.7, Na_v_1.8 and TRKB in pathological DRG from patients with NP after brachial plexus avulsion. (A-C) Immunostaining against (A) Na_v_1.7/TRKB/NEUN, (B) Na_v_1.8/TRKB/NEUN, or (C) Na_v_1.7/Na_v_1.8/TRKB in sections of normal DRG from human embryos naturally aborted at 29 weeks (upper panel) or sections of pathological DRG from patients (lower panel). (D) Proportions of neurons expressing high (red) or low (blue) levels of Na_v_1.7, Na_v_1.8 and TRKB in normal and pathological DRG. (E) Proportions of Na_v_1.7^+^/TRKB^+^, Na_v_1.8^+^/TRKB^+^ and Na_v_1.7^+^/Na_v_1.8^+^/TRKB^+^ neurons in normal and pathological DRG. (F) Percentages of TRKB neurons expressing Na_v_1.7, Na_v_1.8 and Na_v_1.7/Na_v_1.8. (G) Proportions of TRKB neurons expressing high levels of Na_v_1.7 (Na_v_1.7^High+^) or Na_v_1.8 (Na_v_1.8^High+^). (H) Proportions of TRKB/Na_v_1.7 neurons expressing a high level of Na_v_1.8 (Na_v_1.8^High+^). Expression of high level: fluorescence intensity higher than average intensity. (I) Western blotting of the expression level of Na_v_1.7, Na_v_1.8 and TRKB in normal and pathological DRG of human. Scale bar=100 μm. * *p*<0.05, ** *p*<0.01, **** *p*<0.0001, ^###^ *p*<0.001, ^####^ *p*<0.0001, unpaired Student’s *t* test, n=3-6 DRG sections from 2-3 individuals per condition (D-H), n=3 DRGs from 2-3 individuals per condition (I). Inset at bottom right of each image is higher-magnification views of the boxed small region in corresponded image. It’s noticed that about 60%-70% human DRG neurons expressed TRKB, which is in line with single cell sequencing data of human DRG and non-human primate DRG that showed about 74% and 46% DRG neurons expressing *TrkB* mRNA from Prof. Patrik Ernfors group (https://ernforsluolabs.shinyapps.io/HumanDRG/; SmartSeq2 datasets, <https://ernforsgroup.shinyapps.io/macaquedrg/>) [4-5].


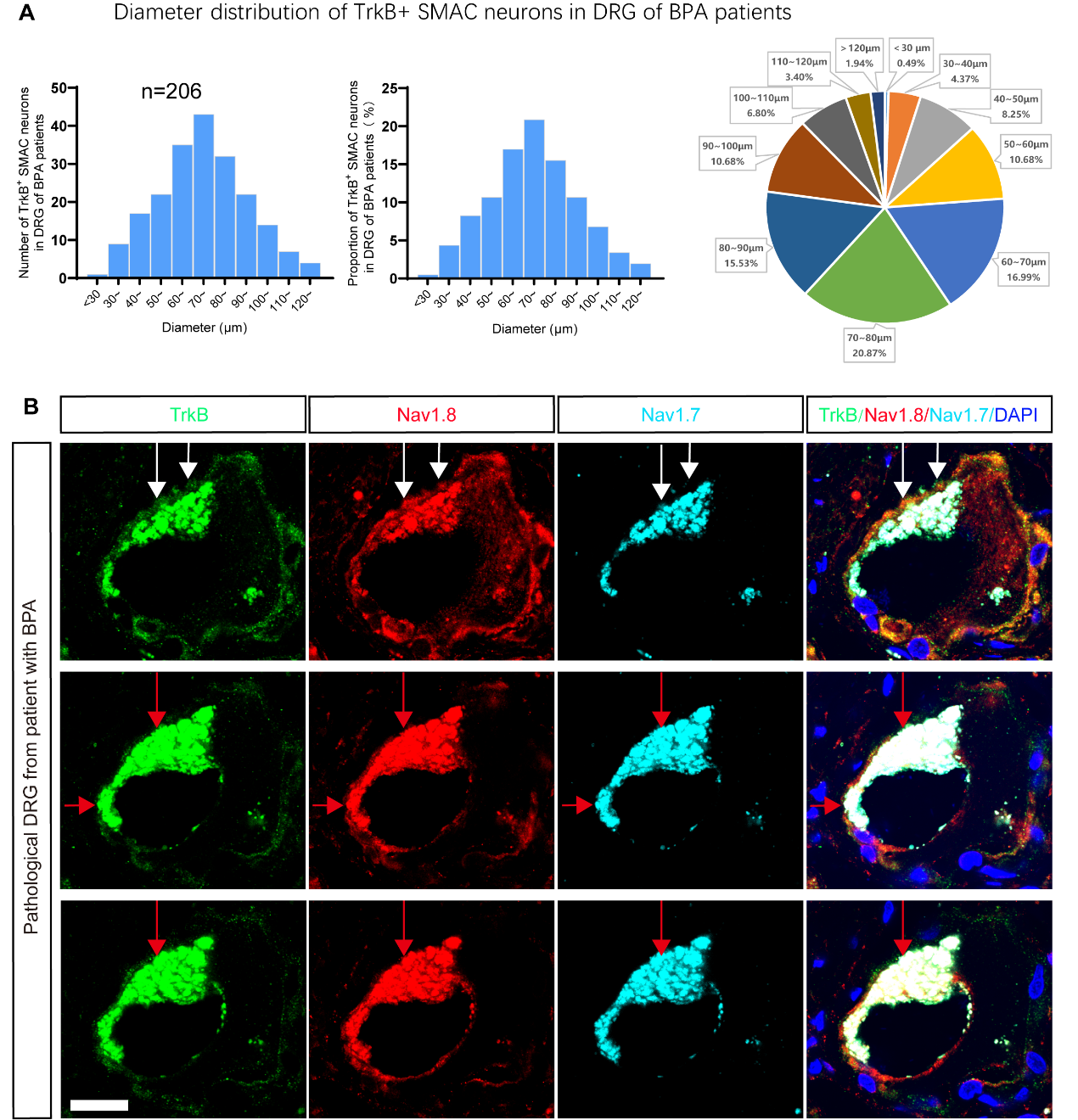
Figure S9. Size of TRKB SMAC neurons, and location of Na_v_1.7/Na_v_1.8/TrkB SMAC in DRG neurons from patients with NP due to brachial plexus avulsion. (A) Quantification of DRG neurons in (Figure S5) revealed that TRKB/Na_v_1.7 SMAC neurons spanned a diameter range of 30–120 μm, with over 73% of these neurons having diameters > 60 μm. (B) Images of three layers of the same DRG neuron with immunostaining for Na_v_1.7 (light blue), Na_v_1.8 (red) and TRKB (green) showed that portions of Na_v_1.7/Na_v_1.8/TRKB SMAC were located on the plasma membrane (pointed by red arrows), suggesting a direct influence on membrane potential. While white arrows pointed to the plasma membrane on which TRKB and Na_v_1.8 were locating diffusely.


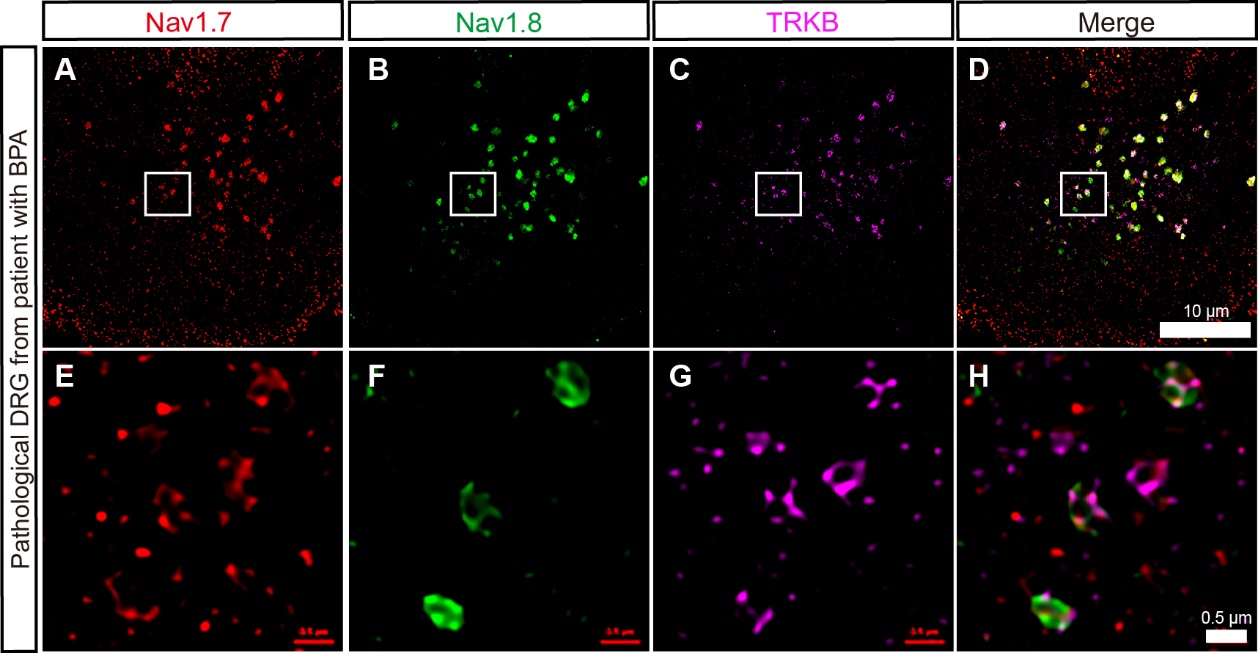
Figure S10. Super resolution images of Na_v_1.7/Na_v_1.8/TrkB SMAC in DRG neuron from patient with brachial plexus avulsion. (A-D) Super resolution images of immunostained SMAC of Na_v_1.7 (A, Red), Na_v_1.8 (B, Green) and TrkB (C, Magenta) in DRG neurons from patient with brachial plexus avulsion (60 nm resolution, Eryla), merged channels (D). (E-H) High magnification view of the boxed area in A-D.


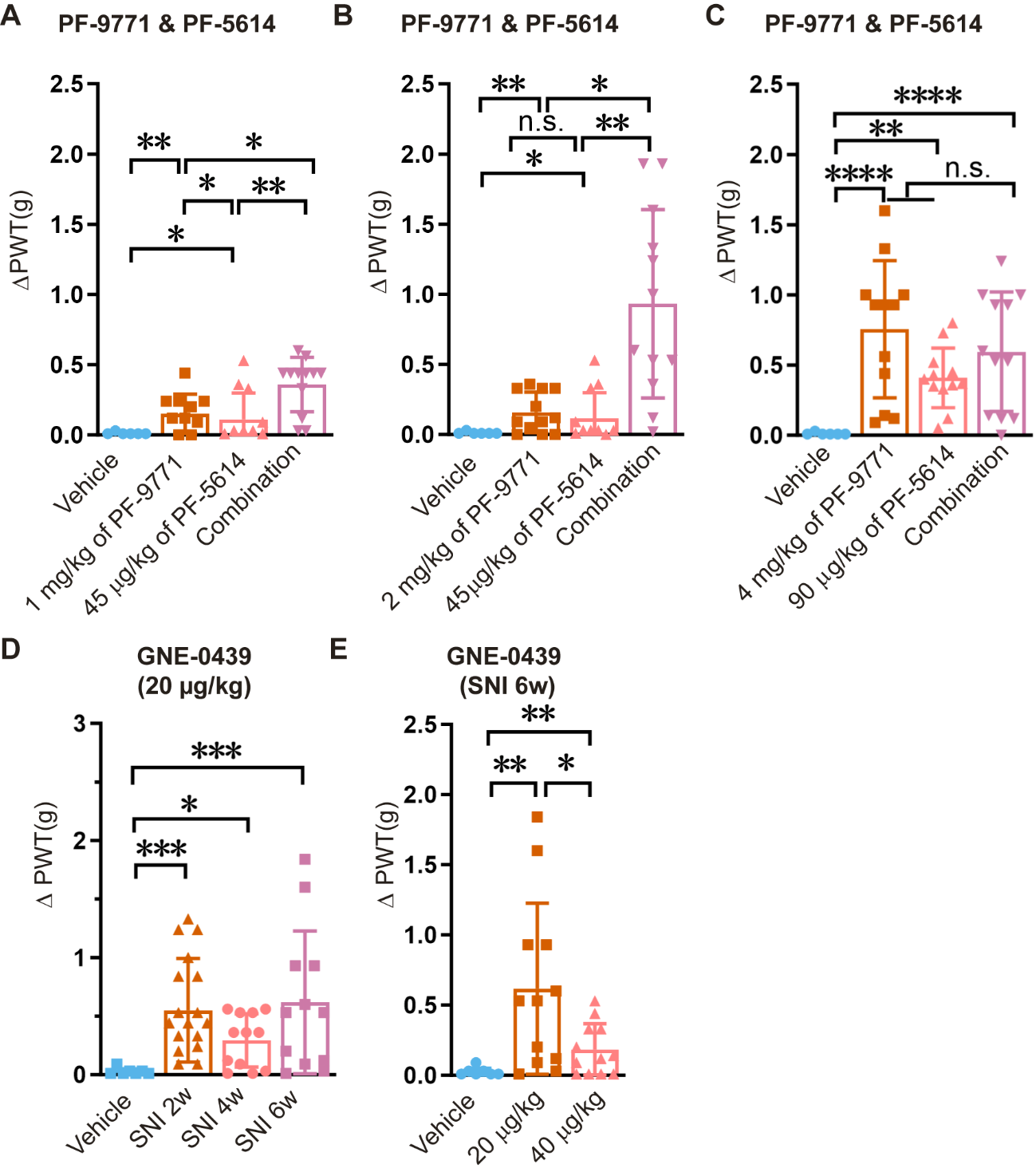


Figure S11. Relief of NP in SNI mice by specific Na_v_1.7/Na_v_1.8 blockers. (A, B) The combination of PF-05089771 and PF-04885614 alleviated pain in SNI mice at 6 weeks post-injury better than the same dose of PF-05089771 and the same dose of PF-04885614. (C) The effects of the combination at a dose of 4 mg/kg PF-05089771 and 90 μg/kg PF-04885614 in SNI mice at 6 weeks post-injury was not better than the same dose of PF-05089771 and the same dose of PF-04885614. (D) Na_v_1.7 blocker GNE-0439 at 20 μg/kg significantly relieved NP in SNI mice at 2, 4, and 6 weeks post-injury, and the effects of GNE-0439 did not weaken from 2 to 6 weeks post-injury. (E) The pain relief efficacy of GNE-0439 decreased when the dose was increased from 20 μg/kg to 40 μg/kg. * *p*<0.05, ** *p*<0.01, *** *p*<0.001, **** *p*<0.0001, Kruskal-Wallis test with Uncorrected Dunn's test, n=12 (A, B and C), n=9-18 animals per condition (D), and n=9-12 animals per condition (E).


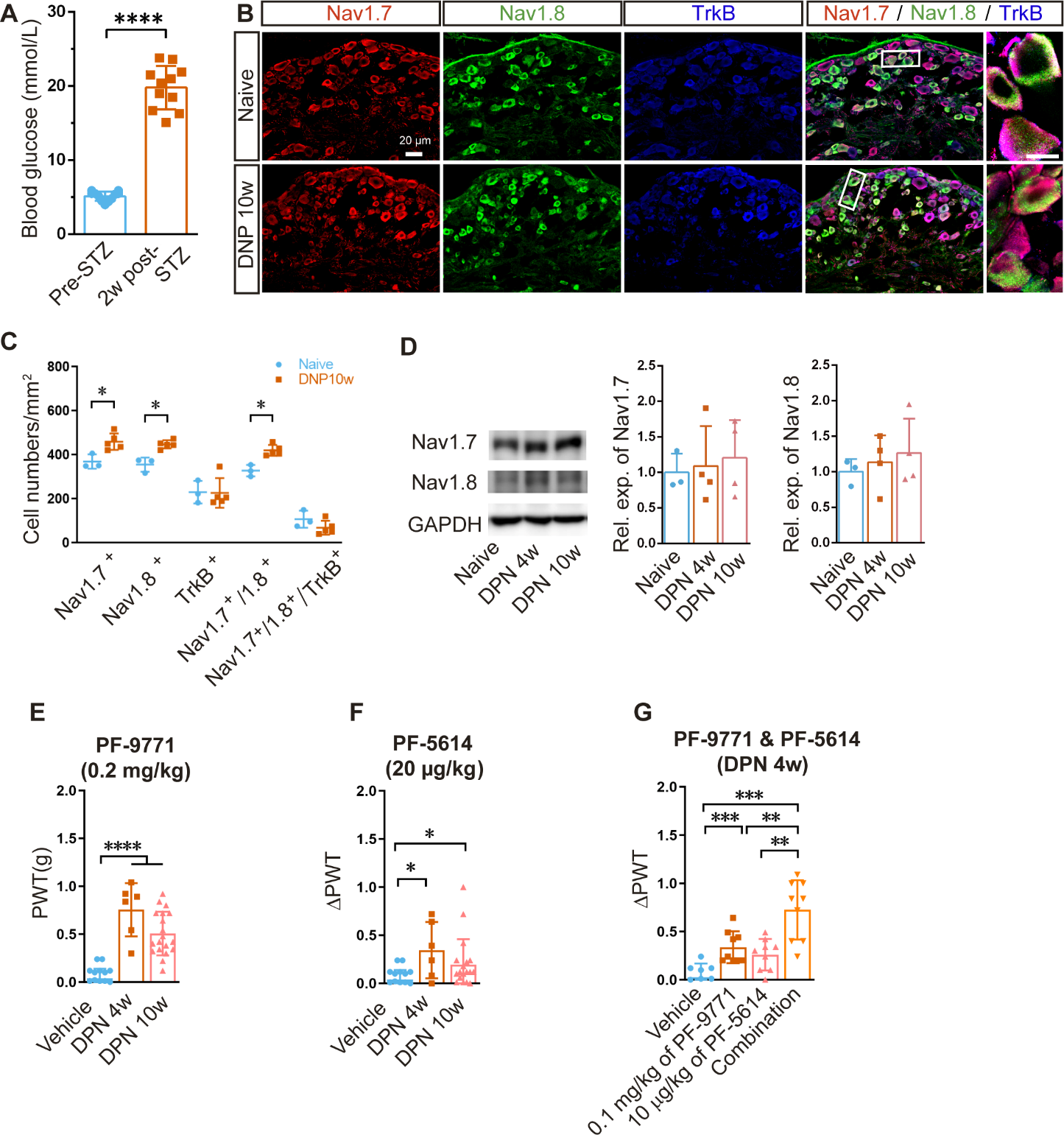


Figure S12. Relief of NP in diabetic mice by Na_v_ blockers. (A) Fasting blood glucose concentration in mice before and after streptozocin (STZ) treatment (n=11). (B, C) Immunostaining against Na_v_1.7, Na_v_1.8 and TrkB in DRG sections from naïve and diabetic mice at 10 weeks after STZ treatment (B), with quantitation of the numbers of different classes of neurons (C). (D) Expression levels of Na_v_1.7 and Na_v_1.8 in DRG of naïve and diabetic mice at the indicated weeks after injury. (E) PF-05089771 at a dose of 0.2 mg/kg significantly alleviated pain in mice at 4 weeks and 10 weeks after the final injection of Streptozocin (STZ), but pain relief efficacy tended to decrease over time. (F) PF-04885614 at a dose of 20 μg/kg significantly alleviated pain in mice at 4 weeks and 10 weeks after the final injection of STZ, but pain relief efficacy tended to decrease over time. (G) The combination of PF-05089771 and PF-04885614 alleviated pain in diabetic mice at 4 weeks after the final injection of STZ better than the same dose of PF-05089771 and the same dose of PF-04885614. * *p*<0.05, ** *p*<0.01, *** *p*<0.001, **** *p*<0.0001, by paired *t* test, or unpaired *t* test with Welch's correction, or one-way ANOVA with Tukey’s test, or Kruskal-Wallis test with Uncorrected Dunn's test, n=5 animals per group (C, D), n=6-19 (F, G), n=9 animals per condition (H). DPN: diabetic peripheral neuropathy.


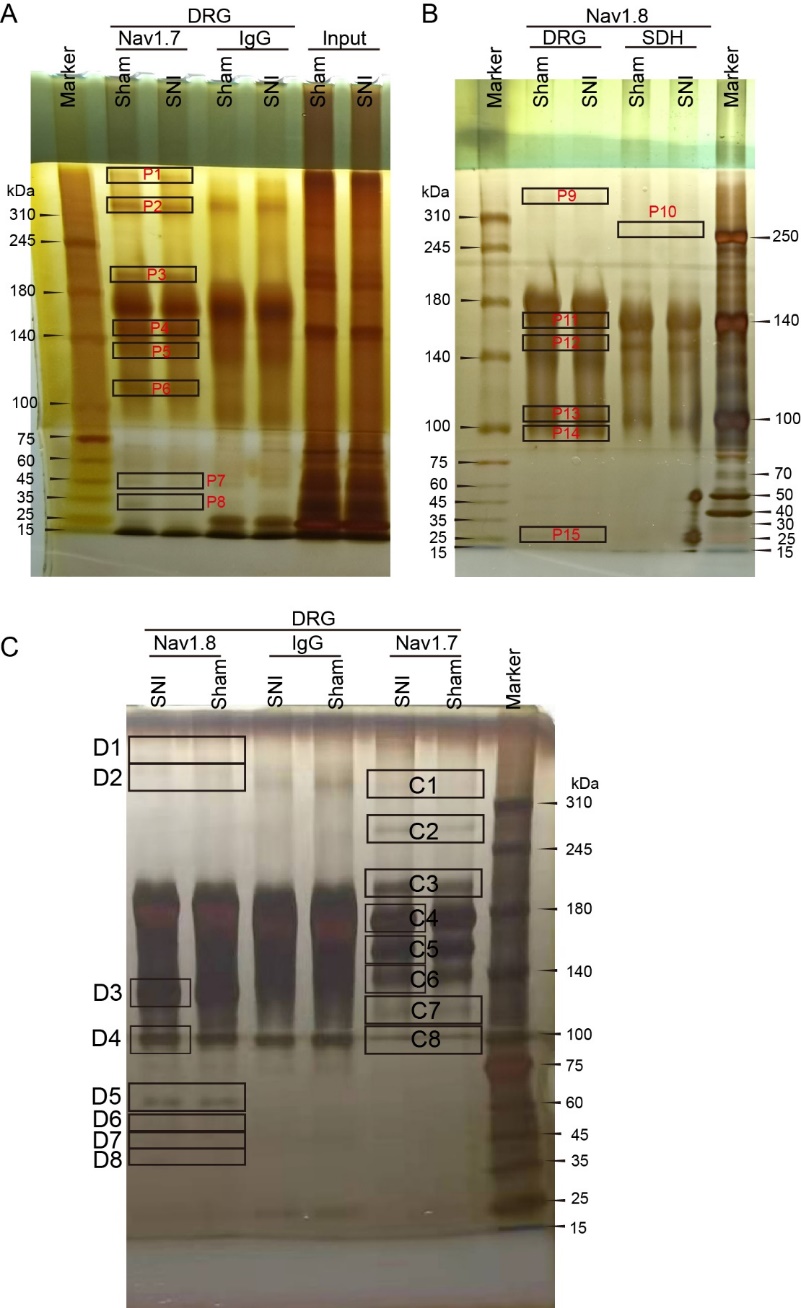
Figure S13. Proteins pulled down by immunoprecipitation using antibodies against Na_v_1.7 or Na_v_1.8. (A) Proteins pulled down by the antibody against Na_v_1.7 or by IgG from DRG lysate of sham and SNI mice at 6 weeks post-injury. Silver-stained gels are shown. The boxed bands (P1-P8) were excised, and the proteins were extracted from gel bands, digested with protease, and identified using mass spectrometry. (B) Proteins pulled down by antibody against Na_v_1.8 from lysate of DRG and spinal dorsal horn (SDH) from sham and SNI mice at 6 weeks post-injury. The boxed bands (P9-P15) were excised and identified by mass spectrometry. (C) Second Pull-down-MS experiment. Proteins pulled down by the antibody against Na_v_1.7 or Na_v_1.8 from DRG lysate of sham and SNI mice at 6 weeks post-injury. The boxed bands (C1-C8, D1-D8) were excised and identified by mass spectrometry.


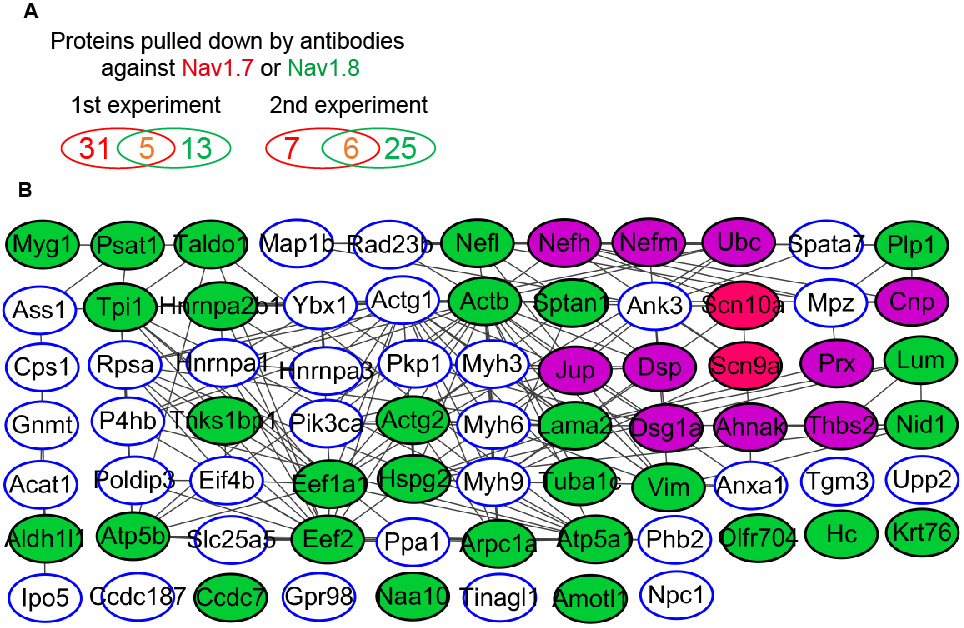
Figure S14. Interaction network of the proteins pulled down by the antibody against Na_v_1.7 and the antibody against Na_v_1.8. (A) Mass spectrometry identified 830 and 301 proteins that were co-immunoprecipitated by the antibodies against Na_v_1.7 or Na_v_1.8 from L4-6 DRG of SNI mice at 6 weeks post-injury in two independent experiments. Among them, 36 and 13 proteins that were pulled down by anti-Na_v_1.7 antibody and 18 and 31 proteins that were pulled down by anti-Na_v_1.8 antibody had molecular weights consistent with the position of bands contained them in SDS-polyacrylamide gels in two experiments, respectively. (B) Interaction network of the proteins was analyzed using STRING APP in Cytoscape [6-8]: 10 proteins in purple (DSP, PRX, DSG1A, AHNAK, THBS2, NEFH, NEFM, UBC, CNP, JUP) were pulled down by both anti-Na_v_1.7 and anti-Na_v_1.8 antibodies. Proteins pulled down only by anti-Na_v_1.7 antibody are circled in blue; those pulled down only by anti- Na_v_1.8 antibody, in green.


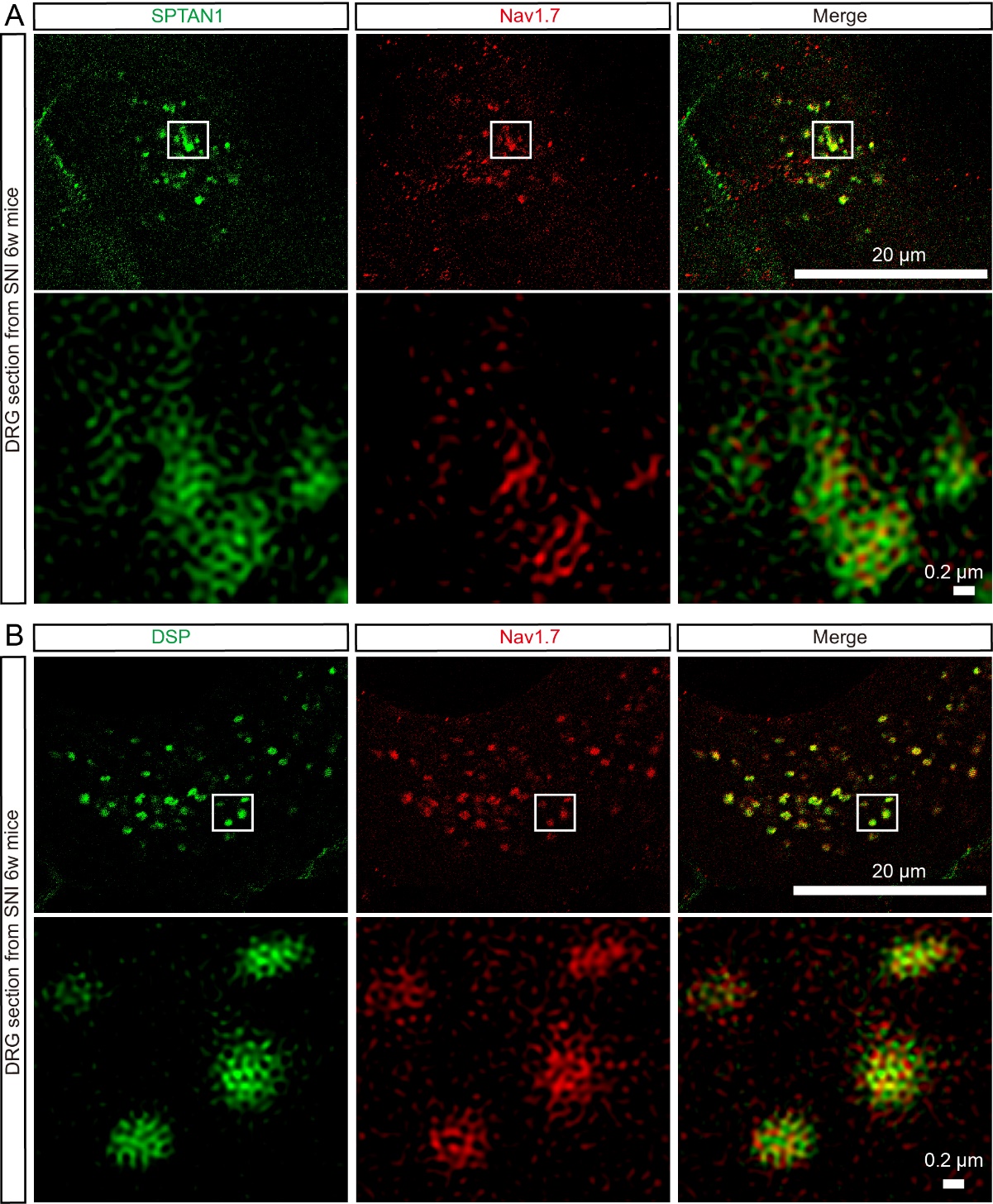
Figure S15. Super resolution images of SMAC in injured mouse DRG neurons. (A) Immunostaining against SPTAN1 (Green) and Na_v_1.7 (Red) in DRG section from SNI mice at 6 weeks post-injury, lower panel is magnification view of the boxed area in upper panel. (B) Immunostaining against DSP (Green) and Na_v_1.7 (Red) in DRG section from SNI mice at 6 weeks post-injury, lower panel is high magnification view of the boxed area in upper panel. Images were photographed by Zeiss Eryla 7 Super resolution microscope.


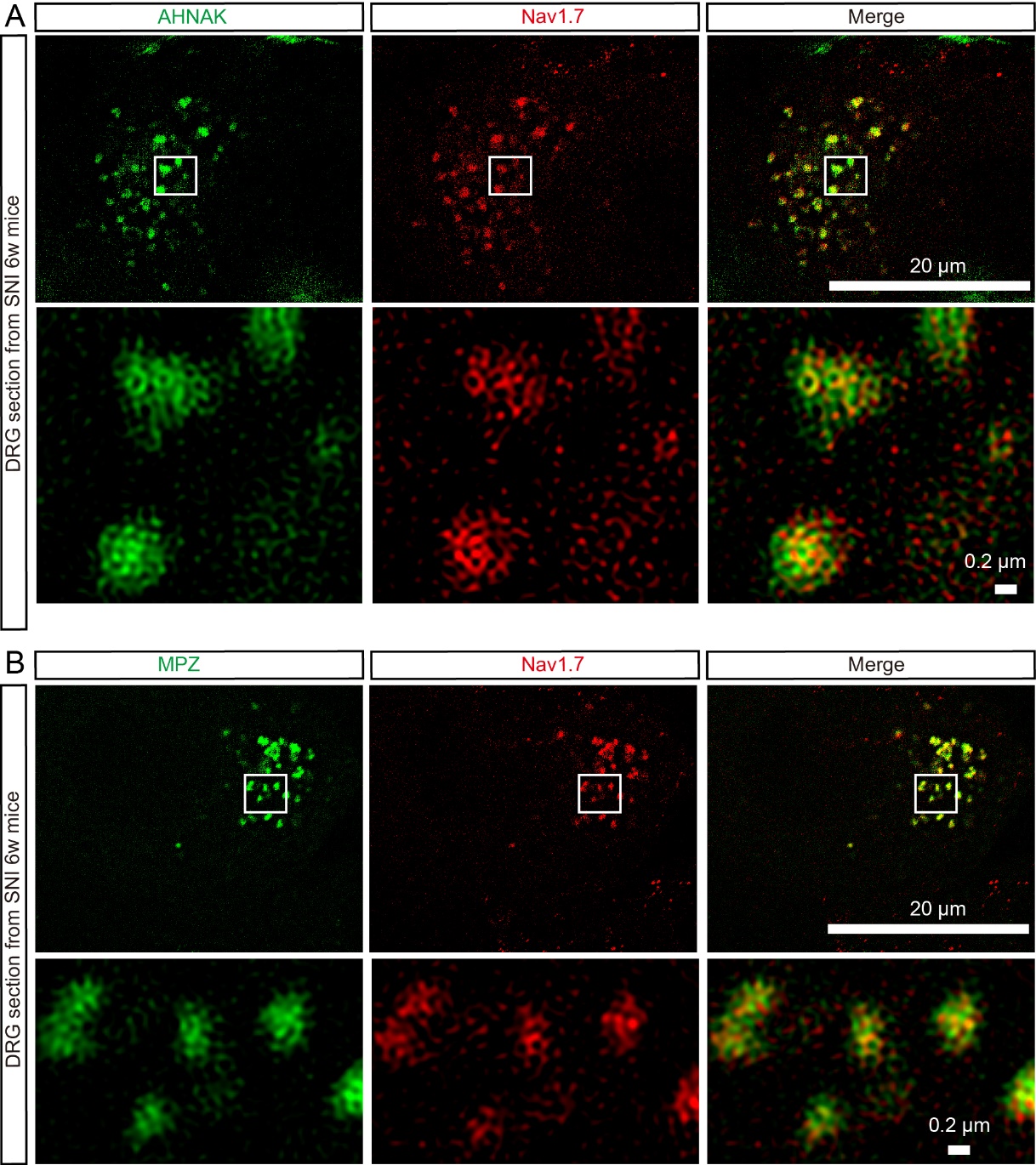
Figure S16. Super resolution images of SMAC in injured mouse DRG neurons. (A) Immunostaining against AHNAK (Green) and Na_v_1.7 (Red) in DRG section from SNI mice at 6 weeks post-injury, lower panel is high magnification view of the boxed area in upper panel. (B) Immunostaining against MPZ (Green) and Na_v_1.7 (Red) in DRG section from SNI mice at 6 weeks post-injury, lower panel is high magnification view of the boxed area in upper panel. Images were photographed by Zeiss Eryla 7 Super resolution microscope.


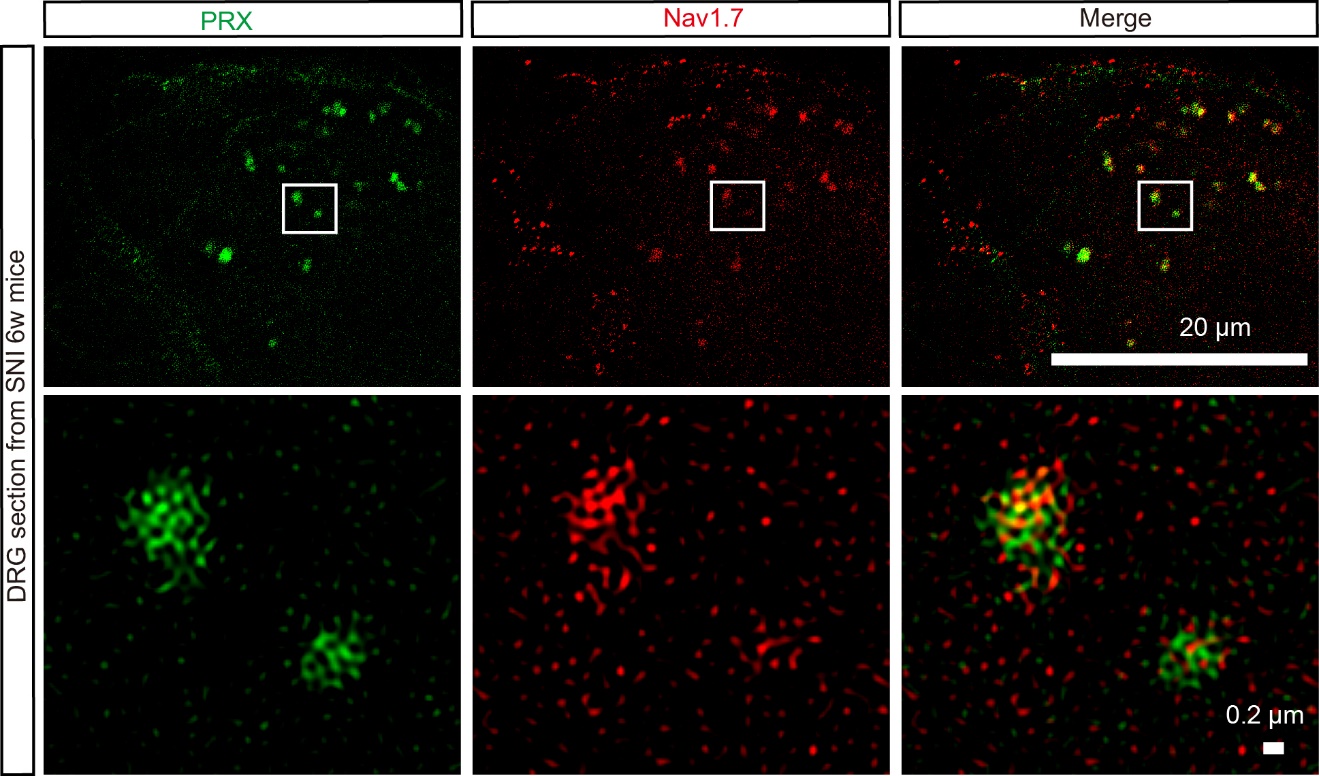
Figure S17. Super resolution images of SMAC in injured mouse DRG neurons. Immunostaining against PRX (Green) and Na_v_1.7 (Red) in DRG section from SNI mice at 6 weeks post-injury, lower panel is high magnification view of the boxed area in upper panel. Images were photographed by Zeiss Eryla 7 Super resolution microscope.


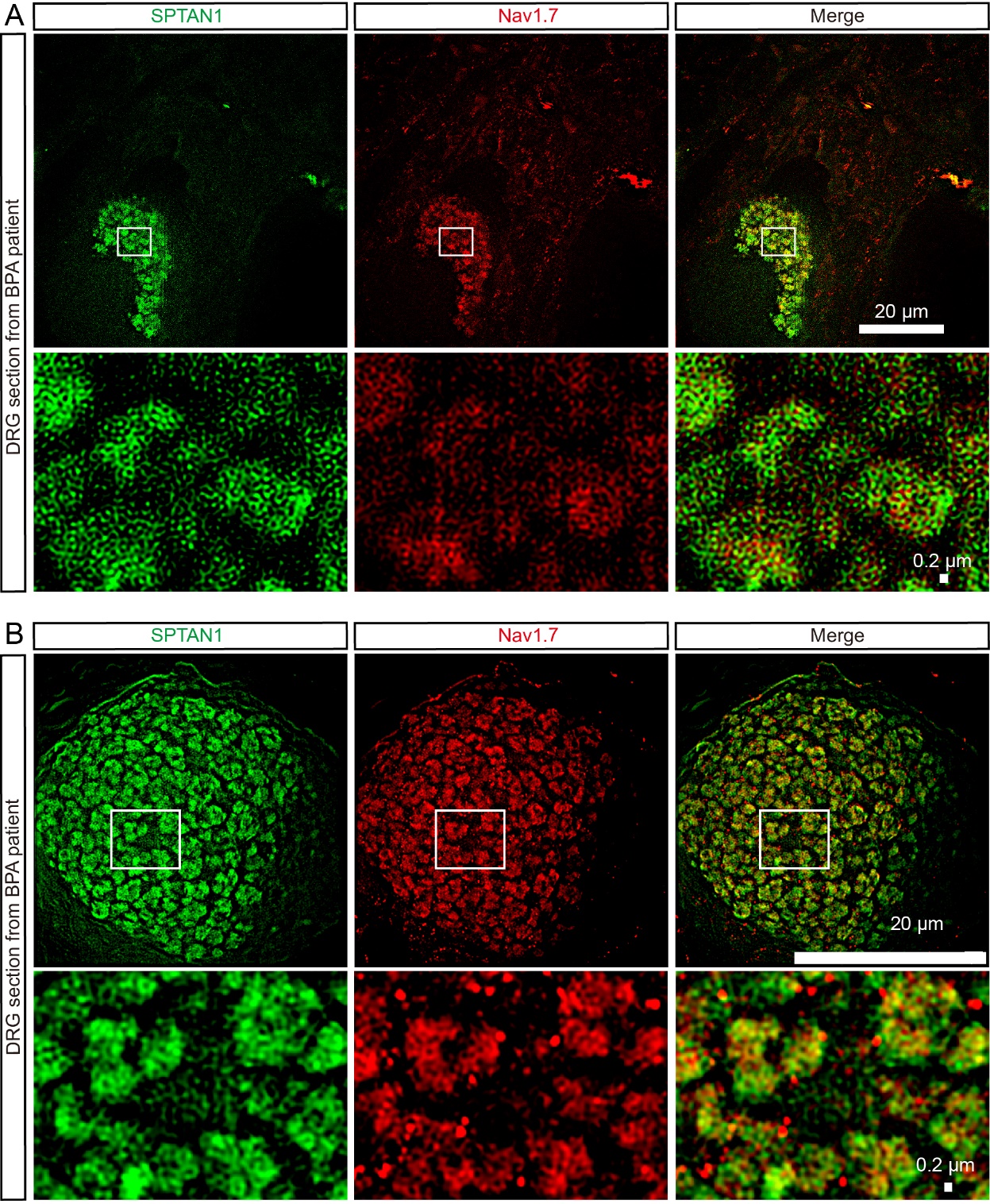
Figure S18. Super resolution images of SMAC in injured human DRG neurons. (A, B) Immunostaining against SPTAN1 (Green) and Na_v_1.7 (Red) in DRG section from BPA patient with NP, images were photographed by Zeiss Eryla 7 Super resolution microscope (A) or HIS-SIM microscope (B). Lower panels in A and B are high magnification views of the boxed area in upper panel of A and B, respectively. Notice: Clusters in SMAC were linked by SPTAN1, see images in lower panel in B.


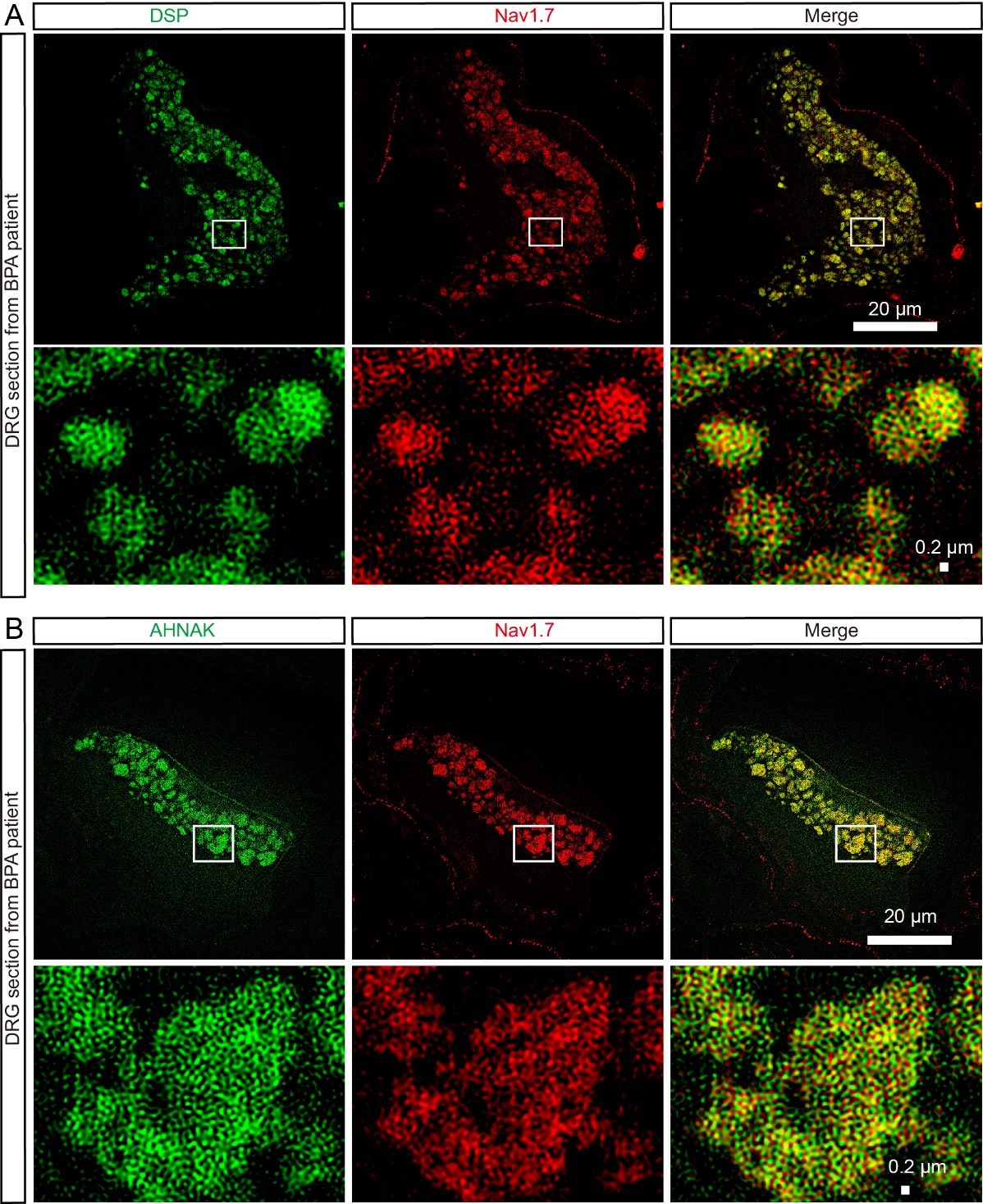
Figure S19. Super resolution images of SMAC in injured human DRG neurons. (A) Immunostaining against DSP (Green) and Na_v_1.7 (Red) in DRG section from BPA patient, lower panel is magnification view of the boxed area in upper panel. (B) Immunostaining against AHNAK (Green) and Na_v_1.7 (Red) in DRG section from BPA patient, lower panel is high magnification view of the boxed area in upper panel. Images were photographed by Zeiss Eryla 7 Super resolution microscope. Notice: Clusters in SMAC were linked by DSP and AHNAK.


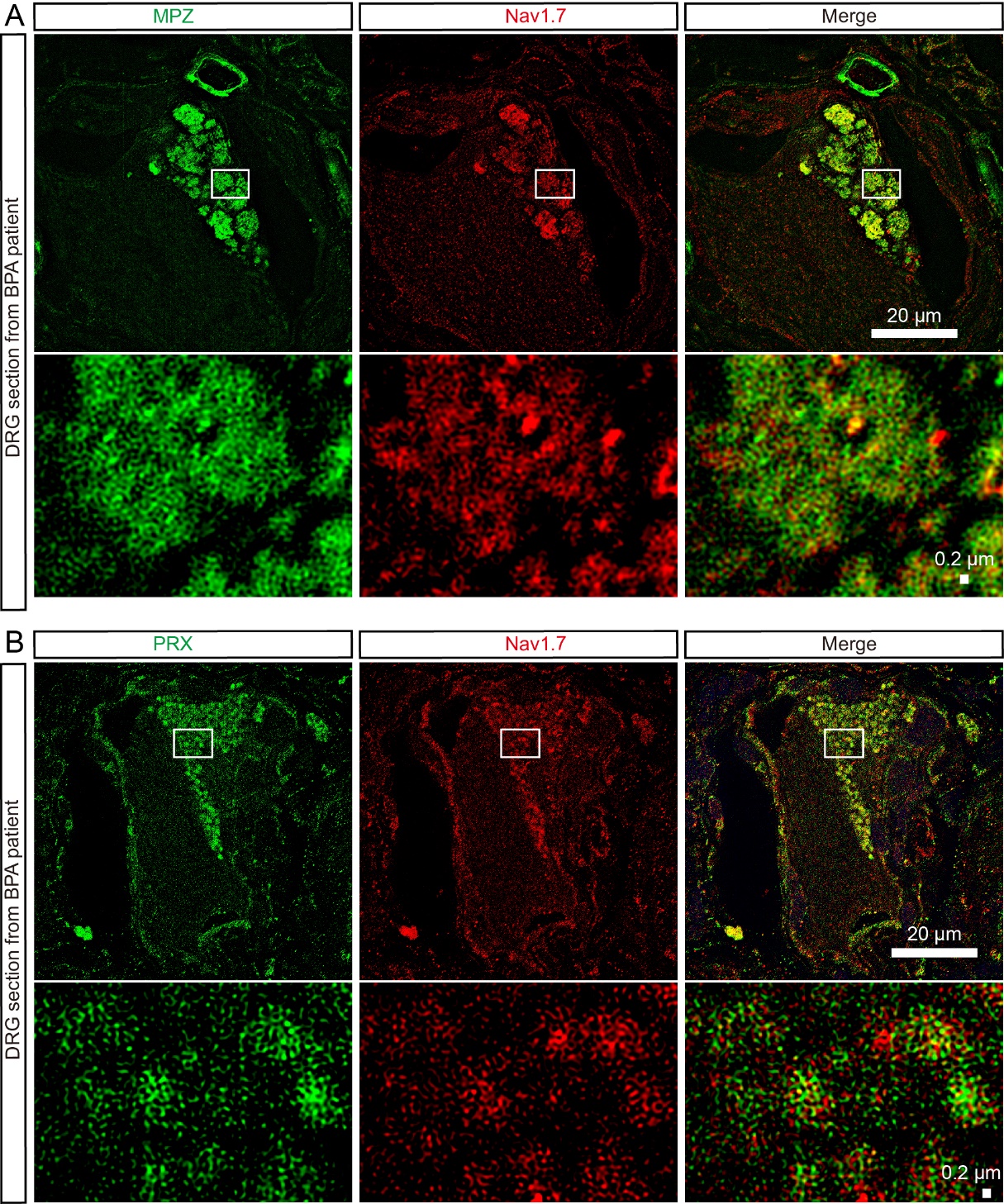
Figure S20. Super resolution images of SMAC in injured human DRG neurons. (A) Immunostaining against MPZ (Green) and Na_v_1.7 (Red) in DRG section from BPA patient, lower panel is magnification view of the boxed area in upper panel. (B) Immunostaining against PRX (Green) and Na_v_1.7 (Red) in DRG section from BPA patient, lower panel is high magnification view of the boxed area in upper panel. Images were photographed by Zeiss Eryla 7 Super resolution microscope. Notice: Clusters in SMAC were linked by MPZ and PRX.


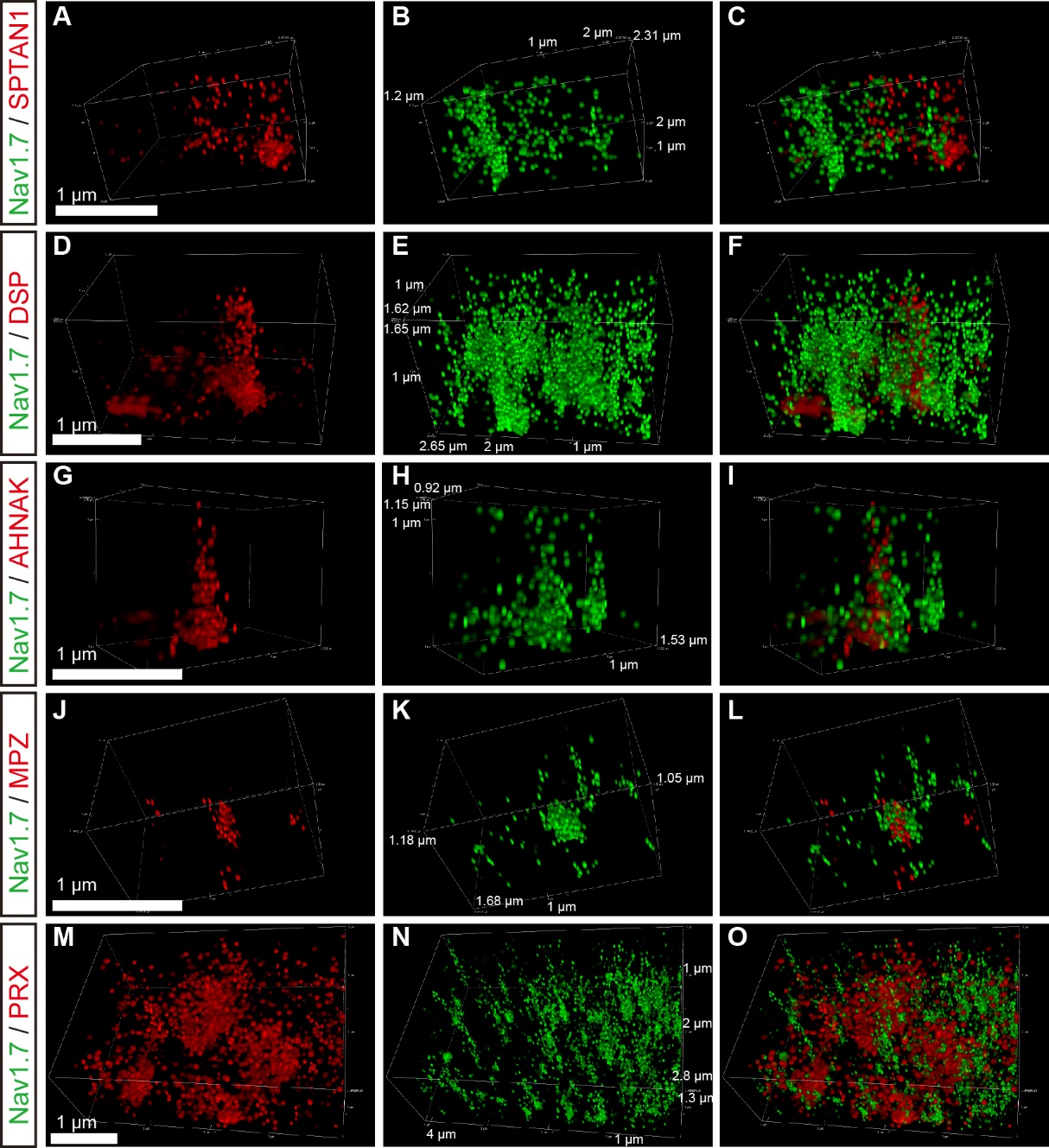


**Figure S21. 3D STORM images of clusters of Na_v_1.7. (A-C)** 3D STORM images of immunostaining against Na_v_1.7 (Green) and SPTAN1 (Red) in primary culture of DRG neurons from SNI mice at 6 weeks post-injury. **(D-F)** 3D STORM images of immunostaining against Na_v_1.7 (Green) and DSP (Red) in primary culture of DRG neurons from SNI mice at 6 weeks post-injury. **(G-I)** 3D STORM images of immunostaining against Na_v_1.7 (Green) and AHNAK (Red) in primary culture of DRG neurons from SNI mice at 6 weeks post-injury. **(J-L)** 3D STORM images of immunostaining against Na_v_1.7 (Green) and MPZ (Red) in primary culture of DRG neurons from SNI mice at 6 weeks post-injury. **(M-O)** 3D STORM images of immunostaining against Na_v_1.7 (Green) and PRX (Red) in primary culture of DRG neurons from SNI mice at 6 weeks post-injury.


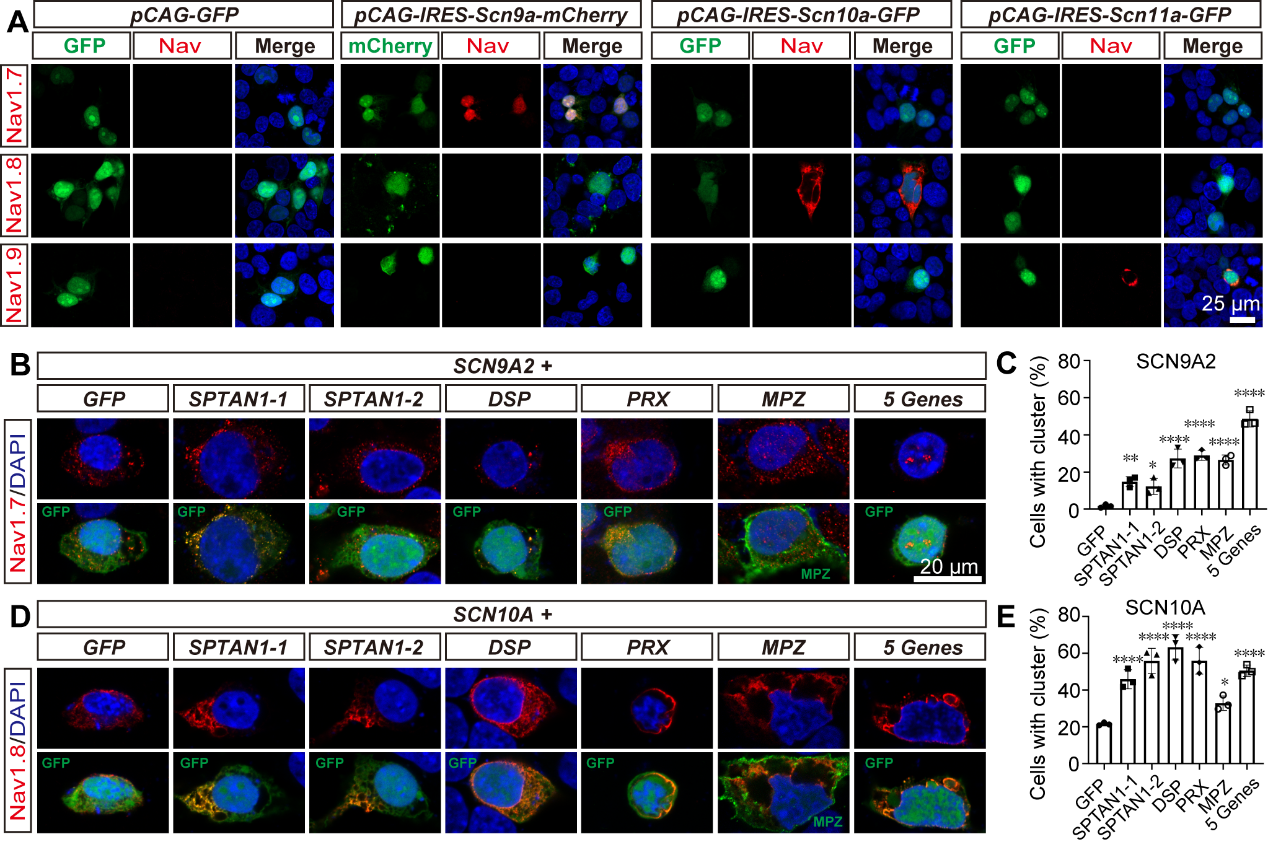
Figure S22. SPTAN1, DSP, PRX and MPZ promoted formation of clusters of Na_v_1.7 and Na_v_1.8. (A) Validation of Na_v_1.7, Na_v_1.8, and Na_v_1.9 antibodies in transfected HEK293 cells. The Na_v_1.7 antibody specifically stained cells transfected with *pCAG-Scn9a-IRES-mCherry* but not those with *pCAG-GFP*, *pCAG-Scn10a-IRES-GFP*, or *pCAG-Scn11a-IRES-GFP*. The Na_v_1.8 antibody exclusively stained cells transfected with *pCAG-Scn10a-IRES-GFP* but not those with *pCAG-GFP*, *pCAG-Scn9a-IRES-mCherry*, or *pCAG-Scn11a-IRES-GFP*. The Na_v_1.9 antibody selectively stained cells transfected with *pCAG-Scn11a-IRES-GFP* but not those with *pCAG-GFP*, *pCAG-Scn9a-IRES-mCherry*, or *pCAG-Scn10a-IRES-GFP*. (B, C) Immunostaining against Na_v_1.7 (red) and GFP (green) or MPZ (green) showed that co-transfection of *SCN9A* with *SPTAN1-1*, *SPTAN1-2*, *DSP*, *Prx,* or *MPZ* individually or all five together promoted formation of Na_v_1.7 clusters in HEK293 cells. Control cells were transfected with *SCN9A* and only green fluorescent protein (GFP). (D, E) Immunostaining against Na_v_1.8 (red), GFP (green) or MPZ (green) showed that co-transfection of *SCN10A* with *SPTAN1-1*, *SPTAN1-2*, *DSP*, *Prx,* or *MPZ*, individually or all five together enhanced cluster formation of Na_v_1.8 in HEK293 cells when compared to the group with co-transfection of *SCN10A* with *GFP*. Cells were counterstained with DAPI. Data are mean ± SD. * *p*<0.05, ** *p*<0.01, *** *p*<0.001, **** *p*<0.0001, Ordinary one-way ANOVA with Tukey's multiple comparisons test or with Uncorrected FishER LSD, n=3 independent technical repeats.

Figure S23. Knockdown efficiency of ShRNAs. (A-E) Quantitative Real-time PCR (qRT-PCR) results showing the knockdown efficiency of ShRNAs targeting *Sptan1*, *Dsp*, *Mpz*, *Prx* or *Ahnak* in HEK293 cells transiently co-transfected with a truncated target gene cDNA and the respective shRNA constructs. Data represent three biological replicates. (F-J) qRT-PCR results demonstrating the knockdown efficiency of ShRNAs targeting *Sptan1*, *Dsp*, *Mpz*, *Prx* or *Ahnak* in DRG following intra-DRG injection of AAV virions. The cDNA was prepared from pooled L4 and L5 DRGs of 3 *WT* mice per group, injected with the indicated ShRNA-expressing virions or left untreated (control). (K) qRT-PCR analysis of *Gfp* expression level in naïve and ShRNA-expressing virion-injected DRGs from three *WT* mice, the AAV vectors used express GFP as a reporter gene. qRT-PCR was performed in three technical replicates. Data are mean ± SD. ** *p*<0.01, **** *p*<0.0001, unpaired *t* test.


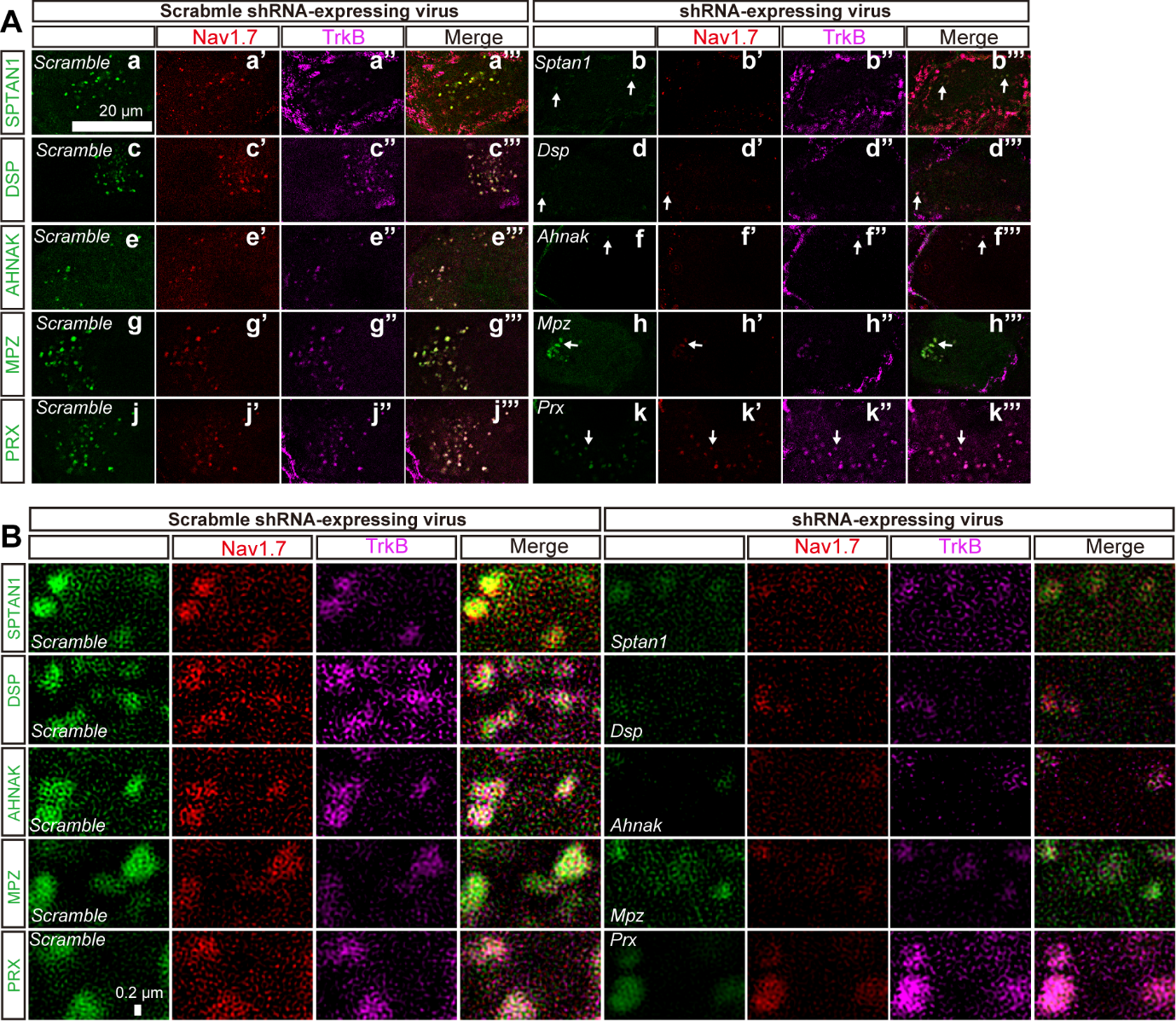
 Figure S24. Knock-down of cytoskeletal proteins reduced the number of SMAC-containing neurons. (A) Immunostaining against SPTAN1 (a, b, Green), or DSP (c, d, Green), or AHNAK (e, f, Green), or MPZ (g, h, Green), or PRX (j, k, Green) and Na_v_1.7 (Red) as well as TrkB (Magenta) and in L5 DRG section from SNI mice with DRG injection of scrambled shRNA-expressing (a, c, e, g, j), shRNA *Sptan1*-expressing (b), shRNA *Dsp*-expressing (d), shRNA *Ahnak*-expressing (f), shRNA *Mpz*-expressing (h), or shRNA *Prx*-expressing (k) AAV viruses. (B) SIM images of immunostaining against Na_v_1.7 (red), TrkB (magenta) and SPTAN1 (green), DSP (green), AHNAK (green), MPZ (green) or PRZ (green) in sections from SNI mice with DRG injection of AAV viruses expressing either scrambled shRNA (left pannel), or shRNA targeting *Sptan1*, *Dsp*, *Ahnak*, *Mpz*, or *Prx* (right pannel).


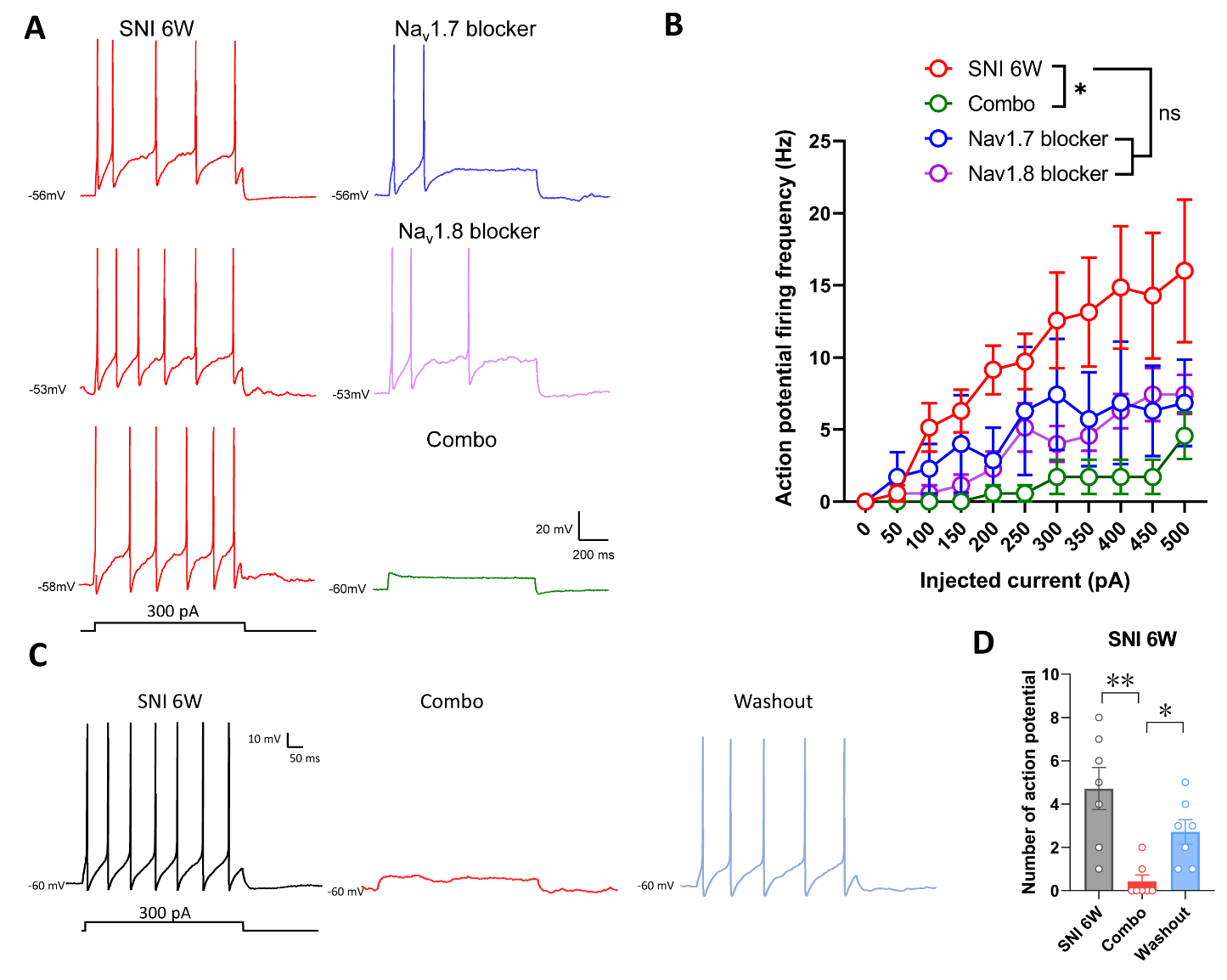


**Figure S25.** **Combined inhibition of Na****_v_1.7 and Na_v_1.8 blocks action potentials in hyperexcitable DRG neurons, with recovery upon washout.**

**(A-B)** Comparison of the Na**_v_**1.7 blocker PF-05089771 (10 μM) and Na**_v_**1.8 blocker PF-04885614 (2.5 μM), alone or together, for inhibiting the multiple firing of neurons on 6 weeks post-injury (n=11 neurons from 2-3 animals per condition). Typical firing diagrams of neurons in whole-mount DRG (A) and quantification of frequency-current relationships (B). **(C)** Typical firing diagrams of neurons in the absence (left) and presence of combined blockers at the indicated doses (middle) and washout (right). **(D)** The combination of Na**_v_**1.7 blocker PF-05089771 (10 μM) and Na**_v_**1.8 blocker PF-04885614 (2.5 μM) significantly inhibited the multiple firing of DRG neurons from SNI mice at 6 weeks post-injury, and recovery upon washout. Data are shown in mean ± SEM from 7 neurons recorded from 2–3 mice. * p<0.05, **** p<0.0001, Kruskal-Wallis test with Uncorrected Dunn's multiple comparisons test.


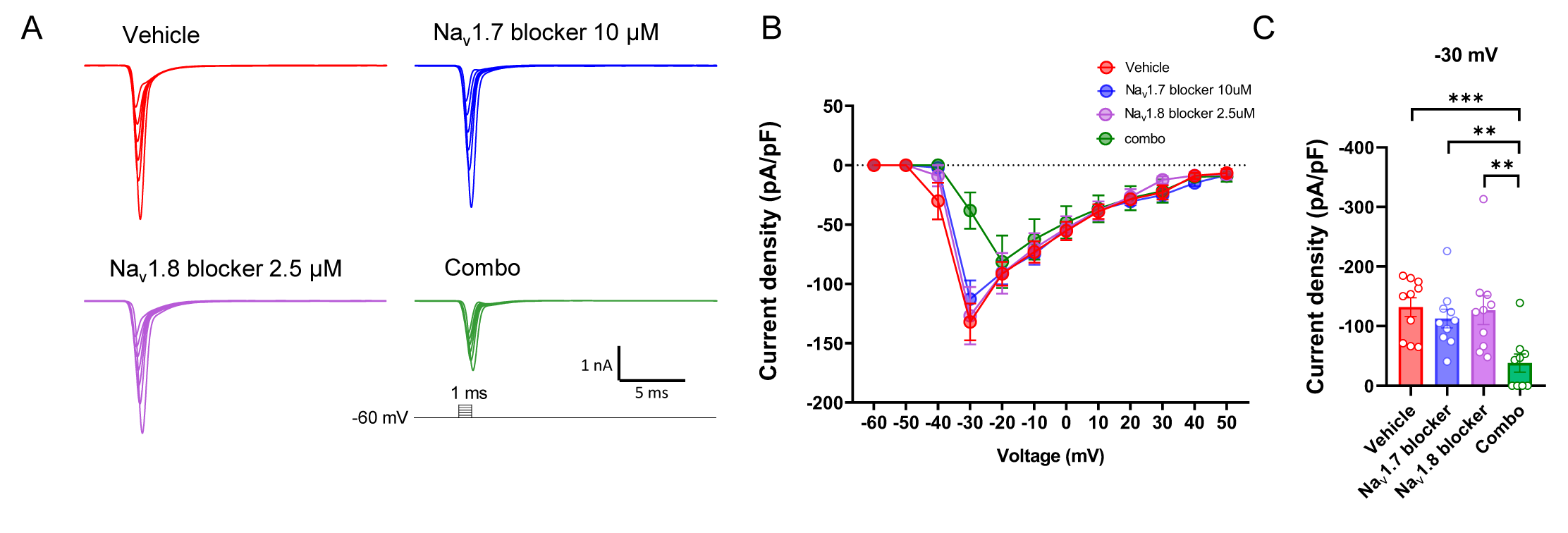
Figure S26. Contribution of Na_v_1.7 and Na_v_1.8 to total sodium current in pathological TrkB^High^ DRG neurons. (A) Typical diagram of total sodium current of TrkB^High^ DRG neurons from *TrkB^2A-Tomato/+^* mice subjected to SNI and treated with vehicle, Na_v_1.7 blocker alone, Na_v_1.8 blocker alone, or their combination at 6 weeks post-injury. (B) Current-voltage relationships for total sodium current in TrkB^High^ DRG neurons from SNI *TrkB^2A-Tomato/+^* mice at 6 weeks post-injury treated with vehicle, Na_v_1.7 blocker alone, Na_v_1.8 blocker alone, or their combination. (C) Neither Na_v_1.7 blocker (10 μM) nor Na_v_1.8 blocker (2.5 μM) on their own significantly inhibited total sodium current in TrkB^High^ DRG neurons at -30 mV, but the combination of both did (n=9-10 neurons from 2 to 3 animals per condition). Na_v_1.7 blocker, Na_v_1.8 blocker and combination reduced sodium current by 15%, 4% and 71%, respectively. Data are shown in mean ± SEM. * *p*<0.05, *** *p*<0.001 by Kruskal-Wallis test.


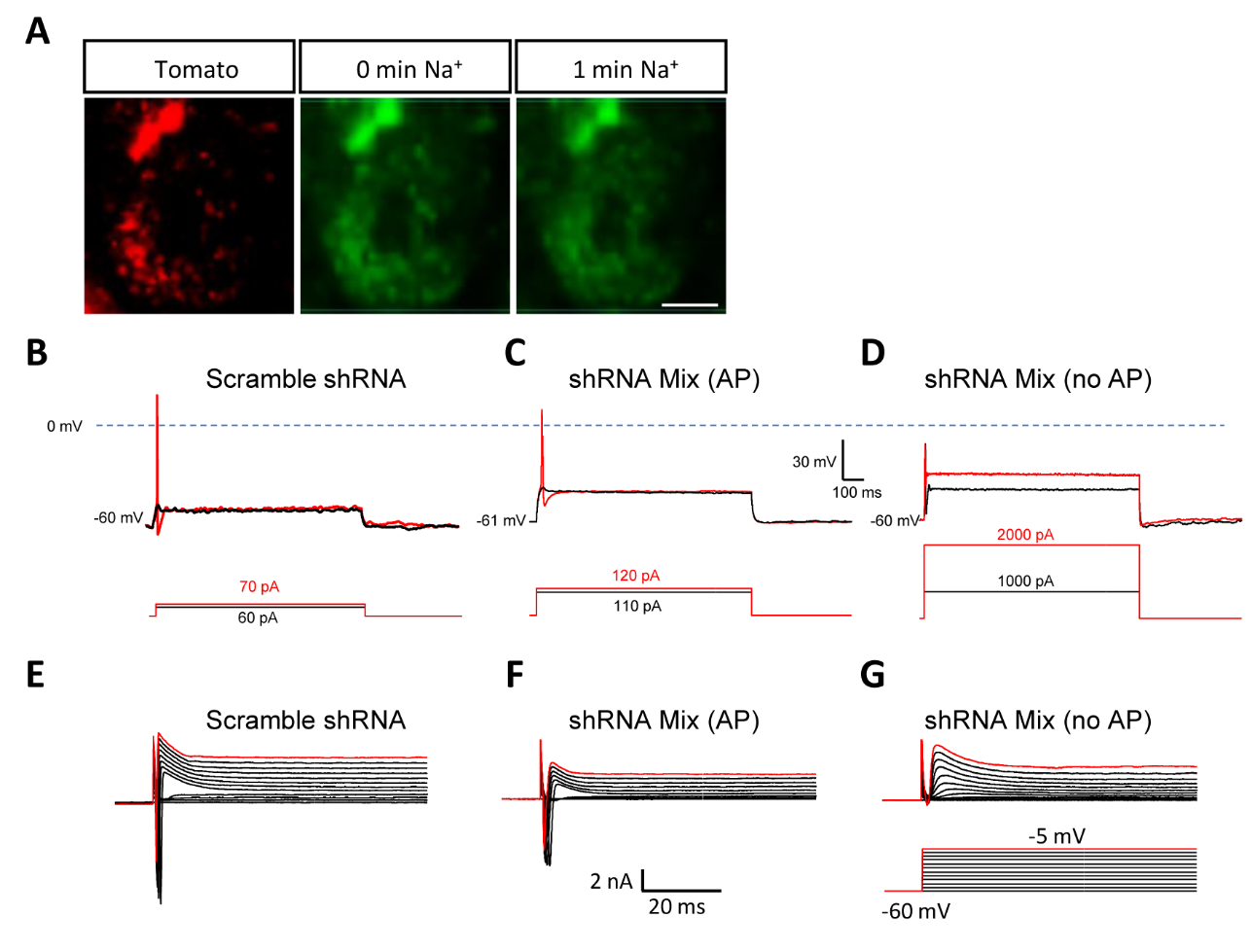


Figure S27. Na⁺ enrichment in SMACs and action potential failure following multi-component SMAC knockdown. (A) Patch-clamp recording of a Tomato⁺ DRG neuron (red) from a *TrkB^2A-Tomato/+^* SNI mouse at 6 weeks post-injury. The neuron was loaded with a sodium indicator (green). Na⁺ signals remained enriched within SAMC regions for several minutes. The right image was captured at 1 minute of continuous recording; later time points are not shown due to slight morphological changes during recording, scale bar=10 µm. (B-D) Typical curves of action potentials triggered by injection of 70 pA currents in scrambled shRNA treated DRG neurons (B), or by injection of 120 pA currents (C) or 2000 pA currents (D) in mixed shRNA (shRNA *Sptan1*, shRNA *Dsp*, shRNA *Ahank*, shRNA *Mpz* and shRNA *Prx*) treated DRG neurons, in SNI mice at 6 weeks post-injury. (E-G) Typical curves of total sodium currents by holding potential from -60 mV to -5 mV in scrambled shRNA (E), or mixed shRNA treated DRG neurons (F, G), in SNI mice at 6 weeks post-injury.

**Table S1. Density of Na_v_1.7 and Na_v_1.8 along long axis in SMAC estimated from STROM image**

| **Region of SMAC** | **Na_v_1.7** **molecule** | **Na_v_1.8 molecule** | **Volume [nm^3^]** | **Na_v_1.7 Density [/μm^3^]** | **Na_v_1.8 Density [/μm^3^]** | **Total Na_v_ Density**  **[/μm^3^]** |
| --- | --- | --- | --- | --- | --- | --- |
| 1 | 7 | 39 | 35354250.36 | 198.00 | 1103.12 | 1301.12 |
| 2 | 26 | 71 | 84627400 | 307.23 | 838.97 | 1146.20 |
| 3 | 32 | 73 | 25165496.07 | 1271.58 | 2900.80 | 4172.38 |
| 4 | 6 | 18 | 66337495.51 | 90.45 | 271.34 | 361.79 |
| 5 | 23 | 48 | 64611096.55 | 355.98 | 742.91 | 1098.88 |
| Mean | 18.8 | 49.8 | 55219147.7 | 340.46 | 901.86 | 1242.32 |
|  |  |  |  |  |  |  |
| Cells | **Estimated Volume of SMAC [μm^3^]** | **Estimated Volume of soma [μm^3^]** | **Na_v_1.7/SMAC** | **Na_v_1.8/SMAC** | **Total Na_v_/SMAC** |  |
| 1 | 679.743 | 22066.6 | 231425.30 | 613033.02 | 844458.32 |  |
| 2 | 1246.239 | 36617.63 | 424294.53 | 1123933.10 | 1548227.63 |  |
| 3 | 385.569 | 20218.5 | 131270.82 | 347729.26 | 479000.08 |  |
| 4 | 444.522 | 7668.2087 | 151341.96 | 400896.61 | 552238.57 |  |
| 5 | 403.344 | 19946.8738 | 137322.50 | 363759.82 | 501082.32 |  |
| Mean | 631.8834 | 21303.56 | 215131.02 | 569870.36 | 785001.39 |  |

**Table S6. Promoter sequences cloned into pGL4.17 vector, primer sequences and truncated mouse gene cDNA sequences cloned into AAV-vector**

| **Gene** | **Sequence 5’ to 3’** |
| --- | --- |
| ***D******sp* promoter WT** | **GGTACC**ATGGTTTCCTGGGATAAGCAAAAAGTCAACATTTCACTAGAGTCGTCCCATGGAGTGACCCTCCCAAGCCACTTTATTTCCGCCTCTTGGGGGAGTAGGAAATCCTCCAAAGGTGTGCCACTGTGGCTCCTAGAGCCTGAAGCAAGTCCCGCGGGGTCGCAGCCCTCCGACGGGGATCCAGTCGCCAGGTCCCCGCCCCTCGCCTCTGCTCAGGCTTCGGTGGCGCAGGGCGGAGCCGCAGGTTGGGGAGGGCGGCCCCAGGCGTCGGTGACACGCGGGGGGGAGGGCGGGGGAGGCGAGGCAGCCGCACCTCGCCCTGGGAAGAAGCC**AAGCTT** |
| ***Dsp* promoter mutant** | **GGTACC**ATGGTTTCCTGGGATAAGCAAAAAGaaAACATTTCACTAGAGTCGTCCCATGGAGTGAaaCTCCCAAGCCACTTTATTTCCGCCTCTTGGGGGAGTAGGAAATCCTCCAAAGGTGTGCCACTGTGGCTCCTAGAGCCTGAAGCAAGTCCCGCGGGGTCGCAGCCCTCCGACGGGGATCCAGTCGCCAGGTCCCCGCCCCTCGCCTCTGCTCAGGCTTCGGTGGCGCAGGGCGGAGCCGCAGGTTGGGGAGGGCGGCCCCAGGCGTCGGTGaaACGCGGGGGGGAGGGCGGGGGAGGCGAGGCAGCCGCACCTCGCCCTGGGAAGAAGCC**AAGCTT** |
| ***Sptan1* promoter WT** | **GGTACC**AAAAGGGTTGTGTATAAGAAAAAAATTACAGAAATTGTGTTTTCCTTTTCTCAGAAGTCTAGCACCCACTTGCTTAACGCTTGTGTTCAAATCTTAGGTTCTCTGATTGCTGCTTCGTGCTTGACCTGGGGAGAGAGCTACCTTCTATGTCATCAGGATGCTAATTTGTAAAATGGGAAAGGCAACATCTACCTTGTAAAATAAGAGCAGAGATTAAAAGCCCAAAGCACGTGCGGTGTCAGCTAAACAGCAAAGTTCAATTAATACTGGGGAAAGAAATAGTAATGTCGTTTATCAACTGGGACGAATACACTCTGTCTACTGGCAACAGGAAGATTGCTGAAGAATTCTCCATATCCTGAAATAAAGTCTTACGCCACAAATCAGT**AAGCTT** |
| ***Sptan1* promoter mutant** | **GGTACC**AAAAGGGTTGTGTATAAGAAAAAAATTaaaagAAATTGTGTTTTCCTTTTCTCAGAAGTCTAGCACCCACTTGCTTAACGCTTGTGTTCAAATCTTAGGTTCTCTGATTGCTGCTTCGTGCTTGACCTGGGGAGAGAGCTACCTTCTATGTCATCAGGATGCTAATTTGTAAAATGGGAAAGGCAACATCTACCTTGTAAAATAAGAGCAGAGATTAAAAGCCCAAAGCACGTGCGGTGTCAGCTAAACAGCAAAGTTCAATTAATACTGGGGAAAGAAATAGTAATGTCGTTTATCAACTGGGACGAATACACTCTGTCTaaaTAaaAACAGGAAGATTGCTGAAGAATTCTCCATATCCTGAAATAAAGTCTTACGCCACAAATCAGT**AAGCTT** |
| ***Prx***  **promoter WT** | **GGTACC**CCTGCCACCCTGTCCCTGACCCCATACCCTCAGCAGAGGTCCAGATCCCTCAATATGGGGTGCCAGGAGGGTCCACAGAGAGGCCACCCAAGCCAGTGTGAGGGGCCAGCGTGCAGCCCTTGGTCCTGGCCATCCAGGACAATAGAGCAGAAGCTGCTGACTATAGCTTTGGGGGTAATCCCCCAGCCTCAGCCTGGCCCCAGCTCTAGGCCTTCGCTCAGAGAGCTGAGAGCATCTGAGGCAGCGGCCTTGTGGGTGACAGAGGC**AAGCTT** |
| ***Prx***  **promoter mutant** | **GGTACC**CCTGCCACCCTGTCCCTGAaaCCATACCCTCAGCAGAGGTCCAGATCCCTCAATATGGGGTGCCAGGAGGGTCCACAGAGAGGCCACCCAAGCCAGTGTGAGGGGCCAGCGTGCAGCCCTTGGTCCTGGCCATCCAGGACAATAGAGCAGAAGCTGCTGACTATAGCTTTGGGGGTAATCCCCCAGCCTCAGCCTGGCCCCAGCTCTAGGCCTTCGCTCAGAGAGCTGAGAGCATCTGAGGCAGCGGCCTTGTGGGTGAaAagaGGC**AAGCTT** |
| ***Ahnak***  **promoter WT** | **GGTACC**TGCATAGGTGACTCCATTCCTAGGGGTCGGATCTAAGTCTTAGAGTCAGTGTGTTTTGTAGAAGAGGAGAGAGCCTGAAGGCGAGGAACGGGGCGAGGCTGCAAGAAGCCTGGGAACTGGGAGAGGAGGCTGGGGCCTGGGATTAATGTATCTGAGCTTGAGAGAAAGCTCAAAGGTCTGTGTGGAGGCTGGAGGTCGAGGTTGAATCATCTGAGCTTCTTGGCTCTTCTGCAGACTAGGTTGACTCTGGATTCCGACTGTGAATCTGTGCCCCTGCGTGCTTCCAGGAATTTCTTGCCGGTGAACACACACACACTGTTTAAAATGGGGTGGGAAGATCCAGAGCCGGGACAGGAGCAATAGATGGGATAGGGGAGGGGAAAAGGCGACGCCTCCTCGTCCCAAGCTT |
| ***Ahnak***  **promoter mutant** | **GGTACC**TGCATAGGTGAaaCCATTCCTAGGGGTCGGATCTAAGTCTTAGAGTCAGTGTGTTTTGTAGAAGAGGAGAGAGCCTGAAGGCGAGGAACGGGGCGAGGCTGCAAGAAGCCTGGGAACTGGGAGAGGAGGCTGGGGCCTGGGATTAATGTATCTGAaCTTGAGAGAAAGCTCAAAGGTCTGTGTGGAGGCTGGAGGTCGAGGTTGAATCATCTGAGCTTCTTGGCTCTTCTGCAGACTAGGTTGACTCTGGATTCCGACTGTGAATCTGTGCCCCTGCGTGCTTCCAGGAATTTCTTGCCGGTaAAaACACACACACTGTTTAAAATGGGGTGGGAAGATCCAGAGCCGGGACAGGAGCAATAGATGGGATAGGGGAGGGGAAAAGGCGACGCCTCCTCGTCCC**AAGCTT** |
| ***Mpz***  **promoter WT** | **GGTACC**CGGCCACACACAGTGTGACCTCTAAGCCCTTCCGAGCCTCTCCGCCGGGGGAGAAGAGGGGGTGCTAAGATCCCAGGGGCGCGGCCTGCACACCCGCAGGTCCTTTCCCTGTCCCCGCAAGGCACACACCGCTGTCACCCCCTCAGGGACTAATTCAGGTTCCCCCAACGGTCGCGGGCGTGGACTCCTAGCCCCAGGCTAAAGTCACCTCAGCAAGAACGCAGCCATCTTGAGTTCCGGCCTGGACGGAAATGACGCATCGCTCCTCCCCCCGTGTGGCCCTGCCTTT**AAGCTT** |
| ***Dsp cDNA fragment for SiRNA screening*** | **GCTAGC**TGCTGCAAAATTGCTCAGACTGTCTGATGCGGGCGGAGCTGATCGCGCAGCCGGAACTGAAATTCGGAGAAGGGATGCAGCTGGCATGGAACCGAGAGCTGGATGAGTATTTTACACAAGCAGAGGATGGACCACCTGCGCCAGCTGCAGAACATCATCCAGGCCACCTCTCGAGAGATCATGTGGATCAATGACTGCGAGGAGGAGGAGCTGCTCTATGACTGGAGCGACAAGAACACCAAGACCACCATCAAGGAGATATCCATGCAGAAGGAAGACGATTCCAAGAATCTCAGAAACCAGATGGACAGACTCTCCCGGGAGAACCGAGATCTTAAGGATGAGATAGTCAGGCTCAATGACAGCATCTTGCAGGCCACGGAGCAGCGAAGGAGAGCCGAGGAGAACGCCCTGCAGCAGAAAGCCTGTGGCTCGGAAACCATGCAGAAGAAGCAGCGCCTGGAGATTGAACTGAAGCAGGTCATCCAGCAGCGCTCAGAGGACAACGCCAGGCACAAGCAGTCCCTGGGGTGGTCATAGTTGACCCGGAAACCAATAAGGAGATGTCTGTTCAGGAGGCCTACAAGAAAGGTCTCATTGATTATGACACCTTCAAGGAGCTATGTGAACAAGAGTGCGAATGGGAAGA**GAATTC** |
| ***Sptan1 cDNA fragment for SiRNA screening*** | GCTAGC**CTGGCCATTTTGATGCCGAAAATATTAAGAAGAAGCAAGAGGCCCTTGTGGCTCGCTATGAGGCTCTTAAGGAACCCATGGTGGCCCGGAAACAGAAGCTGGCAGATTCTCTTCGTCTGCAGTCCAGAATCTGCTAAAGAAACACCAAGCTTTGCAAGCAGAGATTGCTGGGCATGAACCTCGAATCAAAGCAGTGACACAAAAGGGAAATGCCATGGTGGAGGAAGGCCATTTTGCTGCAGCTCCATCAAGGAGCTGAATGAGCGTTGGCGGTCCCTGCAACAGCTGGCTGAGGAACGTAGCCAGCTCTTGGGCAGTGCACACGAAGTACAGAGGTTTCACAGAGATGCTGATGAAACTAAAGAATGGATTGAAGAGAAGAATCAGGCTCTGAACACAGACAACTATGGCCATGATTTAGCTAGTGTCCAGGCCCTGCAGCGCAAACATGAAGGCTTTGAGAGGGACCTTGCAGCTCTTGGTGACAAGGTGAACTCCCTTGGGGAAATTTGACAAAGCTATCAATGTCCAGGAAGAAAAGATAGCTGCCCTGCAGGCCTTTGCCGACCAGCTCATTGCCGTTGACCACTATGCCAAGGGAGACATTGCAAACCGACGCAATGAGGTCCTTGACCGGTGGCGCCGCCTAAAAGCCCAGATGATTGAAAAAAGGTCAAAACTTGGAGAATCTCAAACACTTCAGCAGTTCAGCACTTCACTGTCCACAAGGATCGGGTGAATGATGTCTGTACTAATGGACAAGACCTCATTAAGAAGAACAATCACCATGAGGAGAACATCTCTTCAAAGATGAAGGGTCTGAATGGAAAGGT**GAATTC |
| ***Prx cDNA fragment for SiRNA screening*** | GCTAGC**ATGGAGGCCAGGAGCCGCAGCGCTGAGGAGCTGAGACGGGCGGAGTTGGTGGAGATTATCGTGGAGACCGAGGCACAGACCGGGGTCAGCGGCTTCAACGTAGCAGGCGGCGGCAAAGAAGGAATCTTTGTCCGTGAGCTGCGAGAGGACTCACCGGCAGCTAAGAGCCTCAGCTTGCAAGAAGGGGACCAGCTGCTGAGTGCCCGTGTGTTCTTTGAGAACTTCAAATATGAGGATGCACTTCGCCTGCTGCAATGCGCAGAGCCCTACAAGGTCTCCTTCTGCTTGAAGCGCACTGTGCCCACCGGGGATCTGGCACTGAGGCCCGGGACGGTGT**GAATTC |
| ***Ahnak cDNA fragment for SiRNA screening*** | GCTAGC**GTGATGCAGAACTCCCCTGCGGCCCGCACTGGGGTGGTCAAGGAGGGGGACCAGATTGTGGGTGCCACCATCTACTTTGACAACCTGCAGTCTGGTGAGGTGACCCAGTTGCTGAATACCATGGGGCATCACACTGTTGGCTTGAAGTTGCACCGTAAAGGGGACCGTTCCCCTGAGCCTGGACAGACCTGGACCCATGAAGTCTTCAGTTCCCGTAGCTCTGAAGTGGTTCTGA**GAATTC |
| ***Mpz cDNA fragment for SiRNA screening*** | GCTAGC**GGTGCTCTCTCCAGCCCTGGCCATTGTGGTTTACACGGACAGGGAAATCTATGGTGCCGTGGGCTCCCAGGTGACCCTGCACTGCTCCTTCTGGTCCAGTGAATGGGTCTCAGATGACATCTCTTTTACCTGGCGCTACCAGCCTGAAGGGGGCCGAGATGCCATTTCGATCTTCCACTATGCCAAGGGACAACCTTACATCGATGAGGTGGGGACCTTCAAAGAGCGCATCCAGTGGGTAGGGGACCCTCGCTGGAAGGATGGCTCCATTGTCATACACAACCTAGACTACAGTGACAACGGCACTTTCACATGTGATGTCAAAAACCCACCGGACATAGTGGGCAAGACCTCTCAGGTCACGCTCTATGTCTTTGAAAAAGTGCCCACTAGGTATGGGGTGGTGTTGGGAGCAGTGATCGGGGGCATCCTCGGGGTGGTGCTGTTGCTGCTGTTGCTCTTCTACCTGATTCGGTACTGCTGGCTGCGCAGGCAGGCTGCCCTGCAGAGAAGGCTCAGTGCCATGGA**GAATTC |
| ***Dsp*** | **Forward: 5’-AGAAACCAGATGGACAGACTC- 3’**  **Reverse: 5’-GCTTCAGTTCAATCTCCAGGC- 3’** |
| ***Sptan1*** | **Forward: 5’-CTTTGCCGACCAGCTCATTG- 3’**  **Reverse: 5’-TCATCTGGGCTTTTAGGCGG- 3’** |
| ***Prx*** | **Forward: 5’-GGTCAGCGGCTTCAACGTA- 3’**  **Reverse: 5’-AGAACACACGGGCACTCAG- 3’** |
| ***Ahnak*** | **Forward: 5’-AGTTGCACCGTAAAGGGGAC- 3’**  **Reverse: 5’-CCACTTCAGAGCTACGGGAAC- 3’** |
| ***Mpz*** | **Forward: 5’-AAATCTATGGTGCCGTGGGC- 3’**  **Reverse: 5’-ATGCGCTCTTTGAAGGTCCC- 3’** |
| ***GFP*** | **Forward****: 5'-CTGGTCGAGCTGGACGGCGACG-3'**  **Reverse: 5'-CACGAACTCCAGCAGGACCATG-3'** |
| ***18 S*** | **Forward: 5'-GCTTAATTTGACTCAACACGGGA -3'**  **Reverse: 5'-AGCTATCAATCTGTCAATCCTGTC -3'** |

**Table S7: Key resources table**

| **REAGENT or RESOURCE** | **SOURCE** | **IDENTIFIER** |
| --- | --- | --- |
| **Antibodies** | | |
| Mouse anti-Nav1.8 | NeuroMab | 75-166, RRID: AB_2183861 |
| Mouse anti-PRX | Santa Cruz | sc-137222, RRID: AB_2168215 |
| Rabbit anti-PRX | Abcam | ab211292 |
| Mouse anti-SPTAN1 (spectrin α II) | Santa Cruz | sc-46696, RRID: AB_671135 |
| Rabbit anti-SPTAN1 | ProteinTech Group | 31676-1-AP, RRID: AB_3670071 |
| Mouse anti-DSP (Desmoplakin) | Abcam | ab16434, RRID: AB_443375 |
| Rabbit anti-DSP | ProteinTech Group | 25318-1-AP, RRID: AB_2880028 |
| Mouse anti-NeuN | Merck | MAB377, RRID: AB_2298772 |
| Rabbit anti-Nav1.7 | ProteinTech Group | 20257-1-AP, RRID: AB_11182704 |
| Rabbit anti-Cleaved Caspase-3 | Cell Signaling Technology | #9661, RRID: AB_2341188 |
| Rabbit anti-HA | Cell Signaling Technology | #3724S, RRID: AB_1549585 |
| Rabbit anti-Dsred (Tomato) | Clontech | #632496, |
| Rabbit anti-NeuN | Cell Signaling Technology | #24307, RRID: AB_2651140 |
| Chicken anti-MPZ (Myelin Protein zero) | Abcam | ab39375, RRID: AB_881430 |
| Rabbit anti-MPZ | Abcam | ab183868, RRID: AB_2895675 |
| Mouse anti-AHNAK | Santa Cruz | sc-390743, RRID: AB_3101882 |
| Goat anti-TrkB | R&D system | AF1494, RRID: AB_2155264 |
| Mouse anti-Nav1.7 | Abcam | ab85015, RRID: AB_2184346 |
| Mouse anti-GAPDH | ProteinTech Group | 60004-1-Ig, RRID: AB_2107436 |
| Mouse anti-beta-ACTIN | Sigma-Aldrich | A5441, RRID: AB_476744 |
|  |  |  |
| **Biological samples** |  |  |
| Adult DRG tissue from BPA patients | Xijing Hospital | N/A |
| Embryonic DRG | Dali University | N/A |
|  |  |  |
| **Chemicals, peptides, and recombinant proteins** | | |
| PF-05089771 | Tocris Bioscience | #5931/50 |
| PF-04885614 | Tocris Bioscience | #4916/10 |
| PF-04531083 | Sigma-Aldrich | PZ0273 |
| GNE-0439 | ProbeChem | PC-62325 |
|  |  |  |
| **Critical commercial assays** | | |
| the Revert Aid First strand cDNA synthesis Kit | Thermo Fisher Scientific | K1621 |
| Dynabeads™ Protein A Immunoprecipitation Kit | Invitrogen | 10006D |
| silver staining kit | Beyotime, China | P0017S |
| Deposited data | | |
| Proteome Xchange | This study |  |
| Duolink® In Situ Red Starter Kit Mouse/Rabbit | Merk | DUO92101 |
| Anti-goat MINUS proble | Merk | DUO92006 |
| Experimental models: Cell lines | | |
| HEK293 | ATCC | RRID:CVCL_0045 |
|  |  |  |
| **Experimental models: Organisms/strains** | | |
| *Scn9a^HA^* tag mouse | This study |  |
| *TrkB^2A-Tomato/+^* mouse | This study |  |
|  |  |  |
| **Recombinant DNA** | | |
| pCAG-SPTAN1-1-IRES-GFP | This study |  |
| pCAG-SPTAN1-2-IRES-GFP | This study |  |
| pCAG-Prx-IRES-GFP | This study |  |
| pCAG-DSP-IRES-GFP | This study |  |
| pCAG-MPZ-IRES-GFP | This study |  |
| pCAG-SCN9A1-IRES-mCherry | This study |  |
| pCAG-SCN10A-IRES-GFP | Pengekiphen company Suzhou, China | #P201810 |
| pCAG-SCN9A2-IRES-mCherry | Pengekiphen company Suzhou, China | #P202105 |
|  |  |  |
| **Software and algorithms** | | |
| MaxQuant software version 1.5.3.17 | J. Cox, M. Mann | http://www.maxquant.org |
| STRING database | von Mering, C. et al. | https://cn.string-db.org/ |
| Prism 9.0 | GraphPad | https://www.graphpad.com/ |
| ImageJ | Schneider et al.^7^ | https://imagej.nih.gov/ij/ |
|  |  |  |

**Description of Table S2. (Separate excel file)**

This Microsoft Excel file includes three datasheets. The first is a list of peptides identified by mass spectrometry. The second is the list of proteins determined from unique peptides. The third is the gene name and Gene Ontology term of the proteins identified.

**Description of Table S3. (Separate excel file)**

Identified proteins whose molecular weights were consistent with their band position in SDS-polyacrylamide gels.

**Description of Table S4. (Separate excel file)**

This Microsoft Excel file includes seven datasheets. The first is a list of peptides pulled down by Na_v_1.7 antibody and identified by mass spectrometry. The second is the list of proteins pulled down by Na_v_1.7 antibody and determined from unique peptides. The third is the gene name and Gene Ontology term of the proteins pulled down by Na_v_1.7 antibody. The fourth is a list of peptides pulled down by Na_v_1.8 antibody and identified by mass spectrometry. The fifth is the list of proteins pulled down by Na_v_1.8 antibody and determined from unique peptides. The sixth is the gene name and Gene Ontology term of the proteins pulled down by Na_v_1.8 antibody. The seventh is the proteins identified in two repeated experiments.

**Description of Table S5. (Separate excel file)**

Identified proteins whose molecular weights were consistent with their band position in SDS-polyacrylamide gels.

**Description of Supplementary Video 1.**

Three-dimension movie of **STORM** images of SMAC of Na_v_1.7 (Green) / Na_v_1.8 (Red) in DRG neurons from SNI mice at 6 weeks post-injury.

**Description of Supplementary Video 2.**

Three-dimension movie of **STORM** images of SMAC of Na_v_1.8 (Red) / TrkB (Green) in DRG neurons from SNI mice at 6 weeks post-injury.

**Description of Supplementary Video 3.**

Three-dimension movie of **STORM** images of SMAC of Na_v_1.7 (Red) / TrkB (Green) in DRG neurons from SNI mice at 6 weeks post-injury.

**Description of Video S4.**

Three dimensions movie of confocal images of SMAC of Na_v_1.7 (Red)/Na_v_1.8 (Green)/TrkB (Blue) in DRG neurons from BPA patient with NP.

**Description of Video S5.**

SNI mice at 6 weeks post-injury had a response to stimulus from 0.16 g von Frey hair.

**Description of Video S6.**

SNI mice at 6 weeks post-injury had no response to stimulus from 2 g von Frey hair 1 hour after administration of the combination of Na_v_1.7 blocker and Na_v_1.8 blocker.

**Description of Supplementary Video 7.**

Three-dimension movie of **STORM** images of SMAC of Na_v_1.7 (Green) / SPTAN1 (Red) in DRG neurons from SNI mice at 6 weeks post-injury.

**Description of Supplementary Video 8.**

Three-dimension movie of **STORM** images of SMAC of Na_v_1.7 (Green) / DSP (Red) in DRG neurons from SNI mice at 6 weeks post-injury.

**Description of Supplementary Video 9.**

Three-dimension movie of **STORM** images of SMAC of Na_v_1.7 (Green) / AHNAK (Red) in DRG neurons from SNI mice at 6 weeks post-injury.

**Description of Supplementary Video 10.**

Three-dimension movie of **STORM** images of SMAC of Na_v_1.7 (Red) / MPZ (Green) in DRG neurons from SNI mice at 6 weeks post-injury.

**Description of Supplementary Video 11.**

Three-dimension movie of **STORM** images of SMAC of Na_v_1.7 (Red) / PRX (Green) in DRG neurons from SNI mice at 6 weeks post-injury.

**Description of Video S12.**

SNI mice at 6 weeks post-injury which were injected with AAV-mixed shRNAs in DRG had no response to stimulus from 1 g von Frey hair.

**Description of Video S13.**

SNI mice at 6 weeks post-injury which were injected with AAV-scrambled shRNA in DRG had a response to stimulus from 0.16 g von Frey hair.

**Description of Supplementary Video 14.**

SNI mice which were injected with AAV-scrambled shRNA (left) or AAV- mixed shRNA (right) in DRG had same response to brush stimulation at 8 weeks post-injury.

**Description of Supplementary Video 15.**

SNI mice which were injected with AAV-scrambled shRNA (left) or AAV- mixed shRNA (right) in DRG had same response to pinprick at 8 weeks post-injury.

Supplementary references:

[1] S. Bang, C. Jiang, J. Xu, et al., *Satellite glial GPR37L1 and its ligand maresin 1 regulate potassium channel signaling and pain homeostasis*, *J Clin Invest*, *134* (9), (2024**)**, <https://doi.org/10.1172/jci173537>.

[2] A. McGinnis, R. R. Ji, *The Similar and Distinct Roles of Satellite Glial Cells and Spinal Astrocytes in Neuropathic Pain*, *Cells*, *12* (6), (2023**)**, <https://doi.org/10.3390/cells12060965>.

[3] C. Peng, A. Furlan, M. D. Zhang, et al., *Termination of cell-type specification gene programs by the miR-183 cluster determines the population sizes of low-threshold mechanosensitive neurons*, *Development*, *145* (18), (2018**)**, <https://doi.org/10.1242/dev.165613>.

[4] J. Kupari, D. Usoskin, M. Parisien, et al., *Single cell transcriptomics of primate sensory neurons identifies cell types associated with chronic pain*, *Nat Commun*, *12* (1), (2021**)**: 1510, <https://doi.org/10.1038/s41467-021-21725-z>.

[5] H. Yu, S. S. Nagi, D. Usoskin, et al., *Leveraging deep single-soma RNA sequencing to explore the neural basis of human somatosensation*, *Nat Neurosci*, *27* (12), (2024**)**: 2326, <https://doi.org/10.1038/s41593-024-01794-1>.

[6] D. Szklarczyk, R. Kirsch, M. Koutrouli, et al., *The STRING database in 2023: protein-protein association networks and functional enrichment analyses for any sequenced genome of interest*, *Nucleic Acids Res*, *51* (D1), (2023**)**: D638, <https://doi.org/10.1093/nar/gkac1000>.

[7] N. T. Doncheva, J. H. Morris, H. Holze, et al., *Cytoscape stringApp 2.0: Analysis and Visualization of Heterogeneous Biological Networks*, *J Proteome Res*, *22* (2), (2023**)**: 637, <https://doi.org/10.1021/acs.jproteome.2c00651>.

[8] P. Shannon, A. Markiel, O. Ozier, et al., *Cytoscape: a software environment for integrated models of biomolecular interaction networks*, *Genome Res*, *13* (11), (2003**)**: 2498, <https://doi.org/10.1101/gr.1239303>.
